# Identifying molecular pathways and candidate genes associated with knob traits by transcriptome analysis in the goose (*Anser cygnoides*)

**DOI:** 10.1038/s41598-021-91269-1

**Published:** 2021-06-07

**Authors:** Wangyang Ji, li E Hou, Xiaoya Yuan, Tiantian Gu, ZhuoYu Chen, Yu Zhang, Yang Zhang, Guohong Chen, Qi Xu, Wenming Zhao

**Affiliations:** Key Laboratory of Animal Genetics, Breeding and Molecular Design of Jiangsu Province, Yangzhou University, Yangzhou, 225009 China

**Keywords:** Genetics, Zoology, Anatomy

## Abstract

*Anser cygnoides* has a spherical crest on the beak roof, which is described as knob. However, the mechanisms affecting knob morphology are unclear. Here, we investigated the phenotypic characteristics and molecular basis of knob-size differences in Yangzhou geese. Anatomically, the knob was identified as frontal hump in the frontal area of the skull, rather than hump of upper beak. Although the frontal hump length, and height varied greatly in geese with different knob phenotypes, little was changed in the width. Histologically, knob skin in large-size knobs geese have a greater length in the stratum corneum, stratum spinosum, and stratum reticular than that in small-size knobs geese. Moveover, the 415 differentially expressed genes were found between the large knobs and small ones through transcriptome profiling. In addition, GO enrichment and KEGG pathway analysis revealed 455 significant GO terms and 210 KEGG pathways were enriched, respectively. Among these, TGF-β signaling and thyroid hormone synthesis-signaling pathways were identified to determine knob-size phenotype. Furthermore, *BMP5*, *DCN*, *TSHR* and *ADCY3* were recognized to involve in the growth and development of knob. Our data provide comprehensive molecular determinants of knob size phenotype, which can potentially promote the genetic improvement of goose knobs.

## Introduction

Many birds have a distinctive frontal hump on their heads and most birds have a frontal hump on their cranium. Some birds show helmet-like casques on the dorsal surface of the neurocranium (e.g., *Casuarius *spp.), and some birds display inflated bulges in the frontal area, immediately caudal to the frontal-naso hinge (e.g., *Balearica*). Only a few birds present protuberances of the upper beak (e.g., *Pauxi unicornis*)^1^. Both Chinese geese (*Anser cygnoides*) and African geese (*Anser cygnoides*) possess a spherical crest across the beak roof, which is described as a knob. However, whether the knob is a hump of the upper beak or a frontal crest requires further investigation.

Although all domesticated Chinese and African geese have a characteristic knob-like protuberance across the base of the bill near the forehead, a small protuberance is found in wild specie (swan goose, *Anser cygnoides*). The protuberance has been greatly exaggerated by artificial selection in Chinese and African goose breeds^2^, and then forms the knob, which indicates incompletely dominant inheritance^3^. Generally, the knob size is relatively larger in males than that in females, and in adults than in juveniles. In additon, knob sizes vary among different breeds and within same breeds. For example, Shitou geese have an average knob size of 40 mm, with a maximum of 55 mm and a minimum of approximately 30 mm, while the average knob size of Magang geese is approximately 28 mm. However, the genetic basis underlying differences in knob size phenotype is not fully understood.

The knob, as an ornamental trait, is well developed by the time of sexual maturity and provides an identifier of sexual maturity. Moreover, the knobs influence first impressions of customers in China when making purchase decisions, with a large knob phenotypic size generally being preferred. However, the morphological structures of knobs and the mechanisms underlying phenotypic variation remain unclear. In this study, adult Yangzhou geese with large or small knob phenotype were selected, and knob morphologies and histologies were observed. Furthermore, the genetic basis of knob-size differences was investigated by RNA sequencing technology. These results provide an alternative strategy for the genetic improvement of goose knobs to meet consumer preferences.

## Results

### Phenotype, histology and skull observations

**Figure 1:**
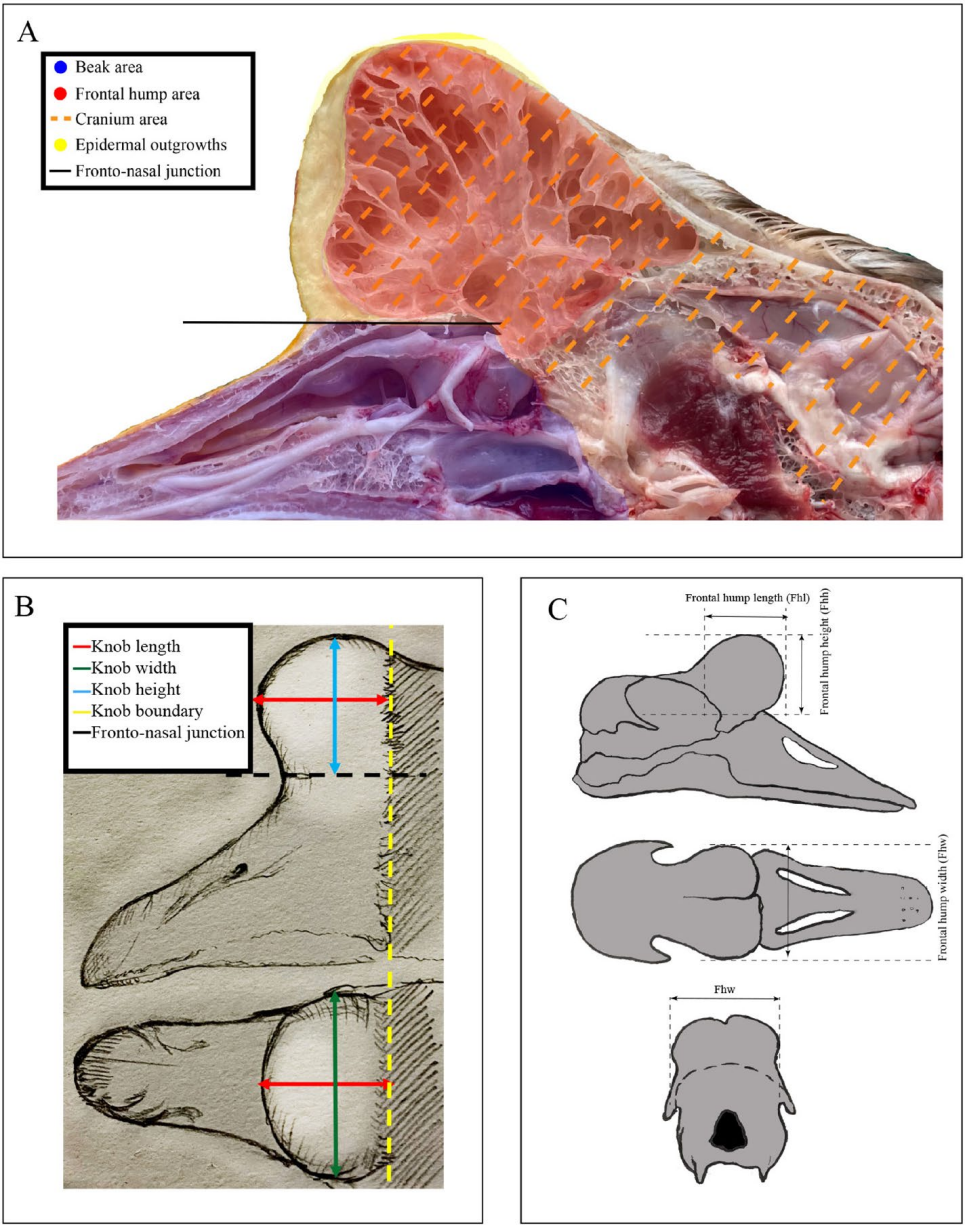
Measurements of the morphometric in goose knob and anatomical measurements made on the goose frontal hump. (**A**) Beak area: blue area; frontal area: red area; cranial area: orange lines; integumentary outgrowth: yellow area; fronto-nasal junction: black line. (**B**) Knob length: red line; knob width: green line; knob height: blue line; the phenotypic boundary of knob: yellow line; fronto-nasal junction: black line. (**C**) From top to bottom: lateral view, dorsal view, and cranial view, respectively.

**Figure 2:**
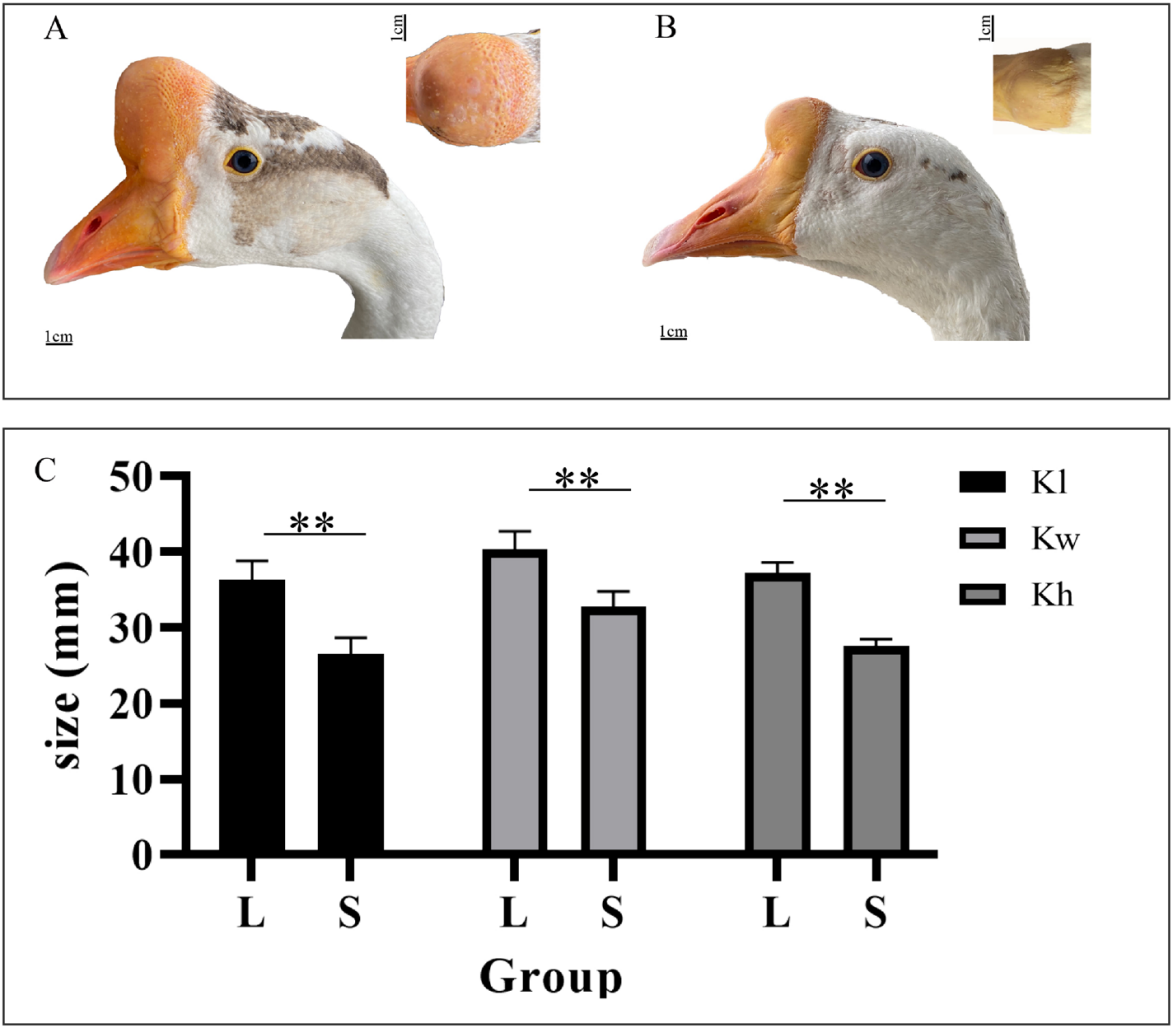
Phenotypes of knobs with different sizes in Yangzhou geese. (**A**,**B**) Differences in knob sizes were observed at 380 days of age between geese with large (L) knobs (**A**) and geese with small (S) knobs (**B**). (**C**) Comparison of knob sizes in the L and S groups. Significant differences are indicated with two asterisks (*P* < 0.01). *Kl* knob length, *Kw* knob width, *Kh* knob height.

Morphological, histological, and anatomical structures of different size knob were observed. The knob in Yangzhou geese accentuated the facial contours (Fig. 1A). Based on the measurement standards (Fig. 1B,C), the knob length, width, and height were dectected (Fig. 2A,B). In Yangzhou geese, the average knob length, width, and height were approximately 31.5 mm, 36.5 mm, 32.4 mm at 380 days of age, respectively, and these parameters varied significantly between large phenotype and small one (*P* < 0.01). The maximum length, width, and height were approximately 36.4 mm, 40.3 mm, and 37.2 mm, and the corresponding minimum values were close to 26.6 mm, 32.8 mm, and 27.6 mm, respectively (Fig. 2C).

**Figure 3:**
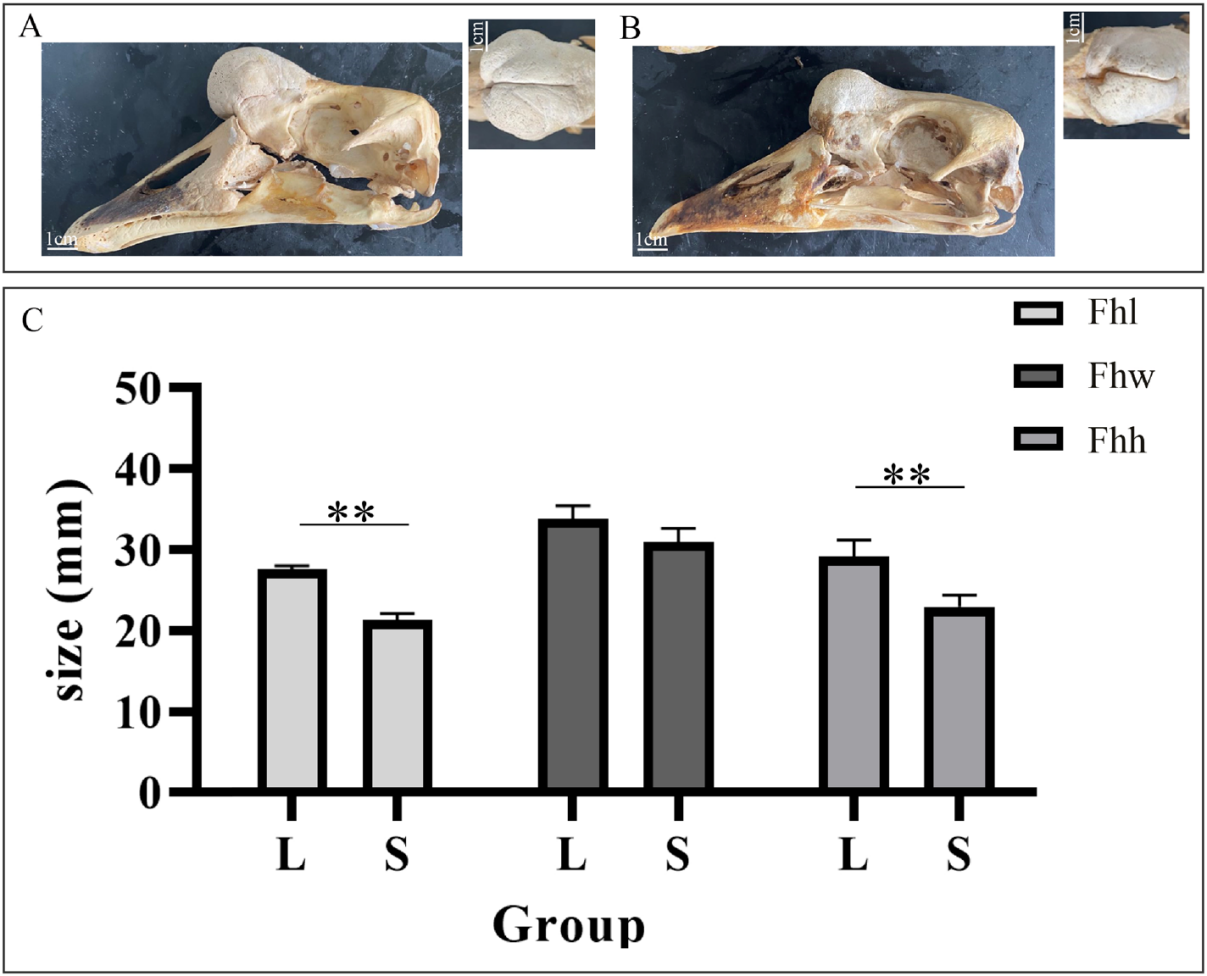
Frontal humps of Yangzhou geese with different anatomical knob sizes. (**A**,**B**) Differences were observed in the frontal hump size at 380 days of age between geese in the L (**A**) and S (**B**) groups. (**C**) Comparison of frontal humps of geese in the L and S groups. Significant differences are indicated with two asterisks (*P* < 0.01). *Fhl* frontal hump length, *Fhw* frontal hump width, *Fhh* frontal hump height.

Anatomically, the knob was found to include the integumentary outgrowth and frontal hump. Although the knob was located at the base of the beak, it was joined to a typically frontal protuberance, rather than on the upper beak (Fig. 1A). Unlike the whole knob phenotype, the length and height rather than the width of the frontal hump were significantly varied between large knob and small one (*P* < 0.01) (Fig. 3A–C). Moreover, The knob length, width, and height were significantly correlated with the corresponding parts in the frontal hump, respectively (Table 1).

**Table 1:** Correlation coefficients between knob and frontal hump dimensions.

Trait	Knob length	Knob width	Knob height
Frontal hump length	0.884**	0.489	0.300
Frontal hump width	0.593	0.862**	0.145
Frontal hump height	0.818**	0.081	0.833**

Significant differences are indicated with two asterisks (*P* < 0.01).

**Figure 4:**
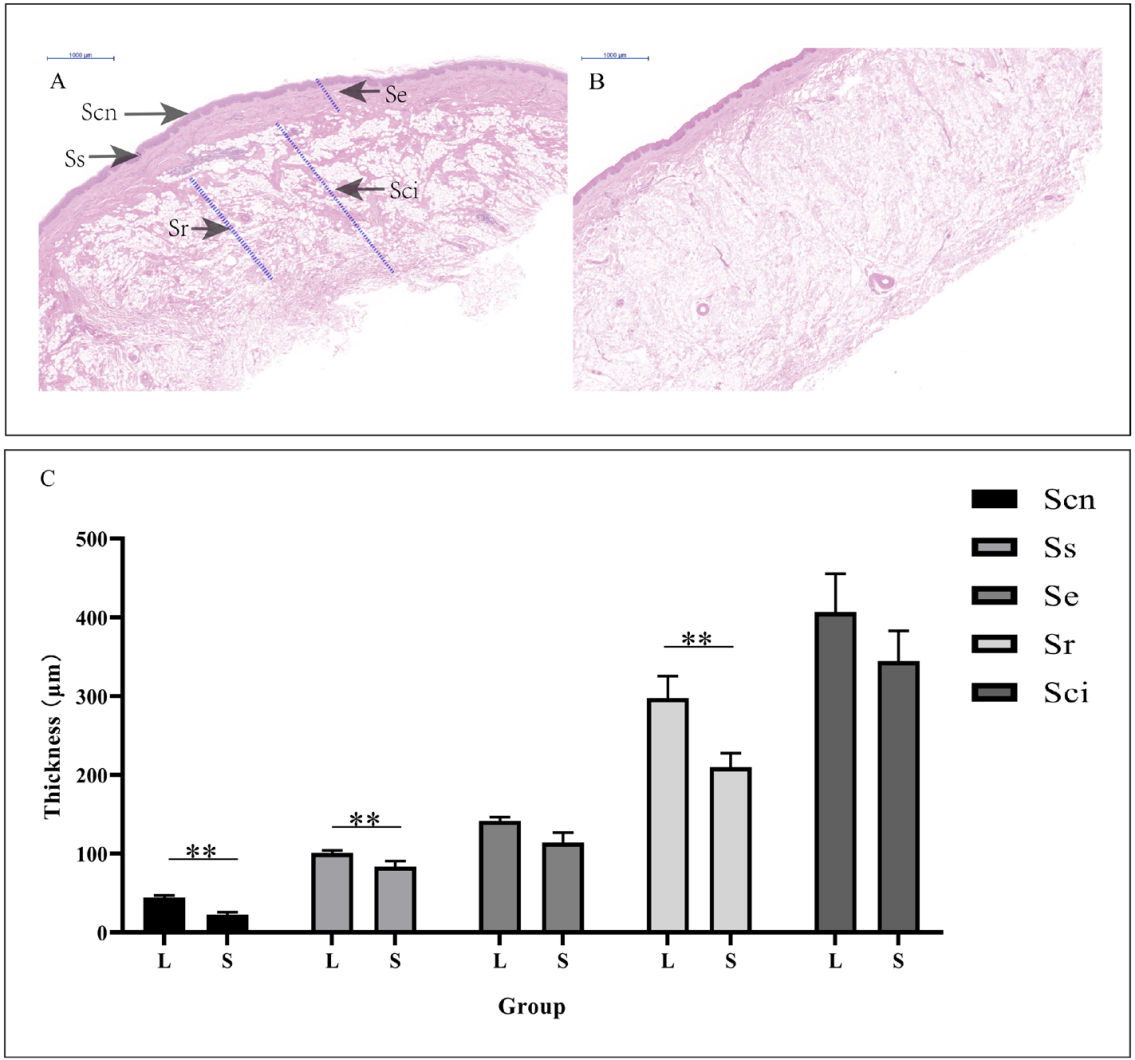
Histological characteristics of knobs with different sizes in Yangzhou geese. (**A**,**B**) Histological analysis of skin from geese with large knobs (**A**) and small knobs (**B**), based on HE staining. *Scn* stratum corneum, *Ss* stratum spinosum, *Se* stratum epidermis, *Sr* stratum reticular, *Sci* stratum corium. (**C**) Comparing the thicknesses of five layers of skin from geese in the L and S groups. Significant differences are indicated with two asterisks (*P* < 0.01).

Histological measurements were performed to determine the thickness of five layers in the skin, including the stratum corneum (Scn), stratum spinosum (Ss), stratum epidermis (Se), stratum reticular (Sr) and stratum corium (Sci). The stratum corneum is composed of flat, dead cells (corneocytes) embedded in a matrix of lipids, which were observed as highly eosinophilic cells with few nuclei following HE staining. Denser structures and significant enrichment for eosinophilic substances were observed in skin tissues from large knobs compared with those from small ones, and subcutaneous tissue shows the same expression. Greater thicknesses of stratum corneum, stratum spinosum, and stratum reticular were also detected in geese with large knobs (*P* < 0.01) (Fig. 4A–C).

### RNA library construction and sequencing

An average of 50,136,474 raw reads was obtained from the large group (L) and small group (S) samples, and the average number of clean reads was 49,500,375. All downstream analyses were based on high-quality, clean sequence data. The error rates were all less than 0.025%. Approximately 92.01–96.37% of the clean reads in the libraries were mapped to the *Anser cygnoides* reference genome and the percentage of phred quality scores of > 20 (Q20) was > 98% in all samples. The sequence read statistics are summarized in Table S2.

### DEG screening in geese with different knob sizes

**Figure 5:**
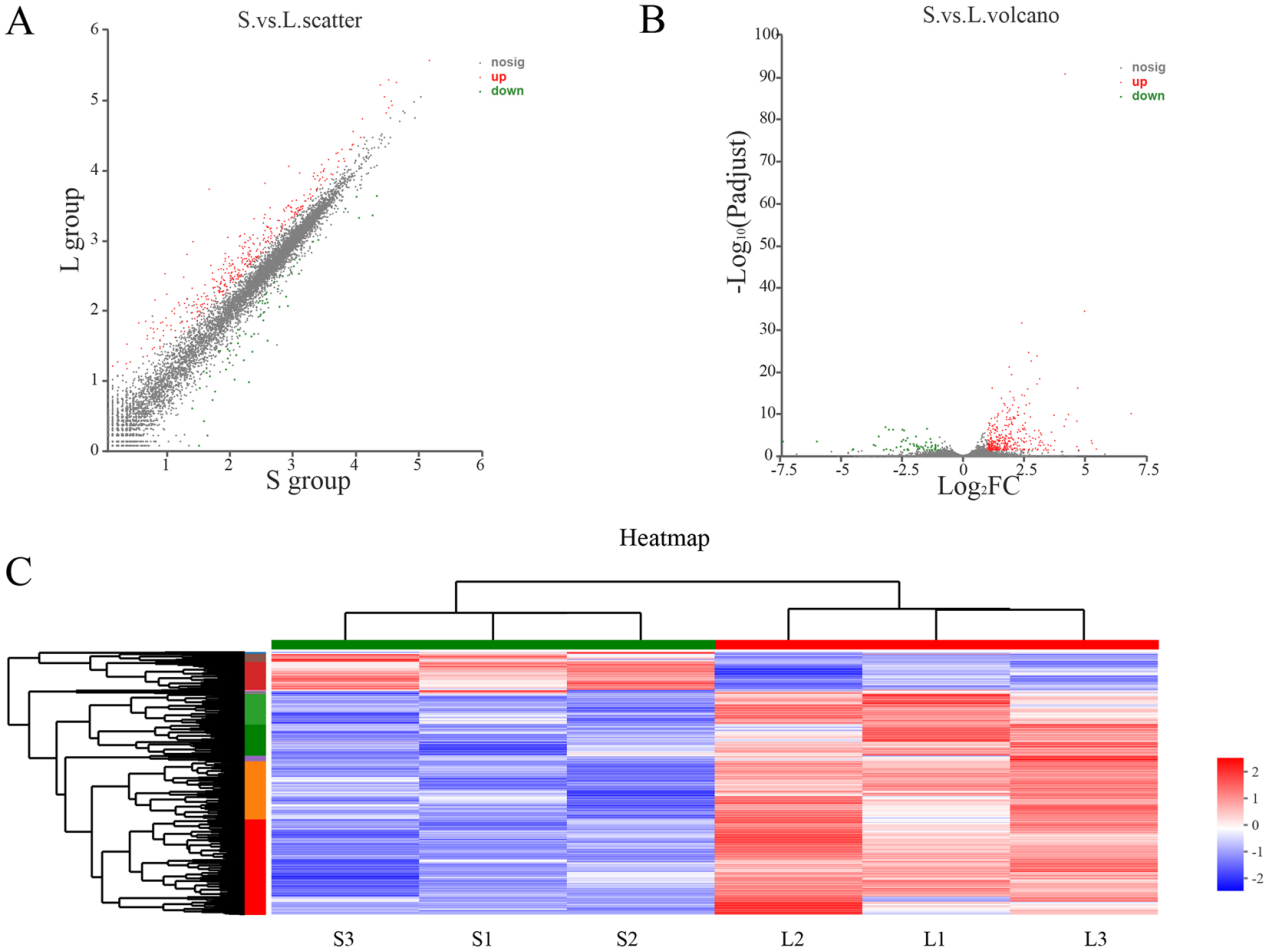
Scatter plot, volcano plot, and heat-map of differentially expressed genes (DEGs). (**A**) The scatter plot was used to assess variations in gene-expression levels between small and large knobs in geese. (**B**) Volcano plot showing DEGs. The X-axis, log_2_FC represents the logarithm of the expression ratio (fold change), and the Y-axis represents adjusted P (P adjust) value. Each dot represents a specific gene. Red dots represent genes expressed at significantly higher levels in geese with large knobs, and green dots represent genes with significantly lower relative expression. Genes showing no significant differences in expression levels are represented with gray dots. (**C**) Hierarchical-clustering analysis for the transcriptome profiles of knobs in the S versus L groups. The heat-map presents the mean relative abundances of genes with a color scale. *FC* fold-change.

DEGs were screened in large and small goose knobs. Scatter and volcano plots showed variations in mRNA-expression levels between geese with small (S group) and large (L group) knobs (Fig. 5A,B). We identified 415 differentially expressed mRNAs (Fig. 5C and detailed information about DEGs is in Table S3; FC ≥ 2), including 357 upregulated genes and 58 downregulated genes in group L comparing with that in group S.

### GO enrichment and KEGG pathway analyses for DEGs

**Figure 6:**
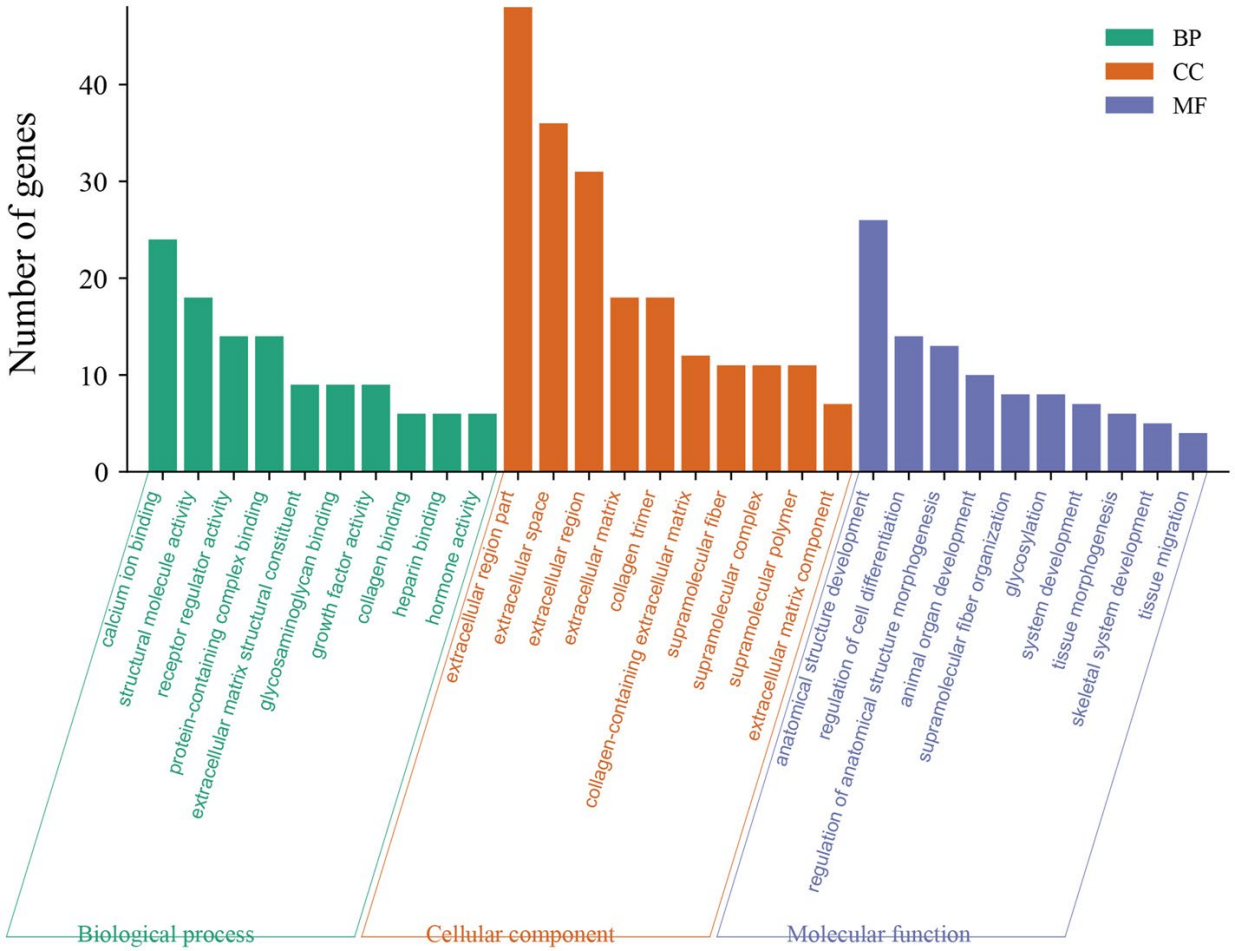
GO analysis of the diferentially expressed genes between the Large knob (L) and small knob (S) groups. The X-axis represents the name of GO terms. The Y-axis represents the numbers of enriched genes in each GO term. The three colors represent the three categories of biological processes (BP), cellular components (CC), and molecular functions (MF), respectively (P adjust < 0.05).

**Figure 7:**
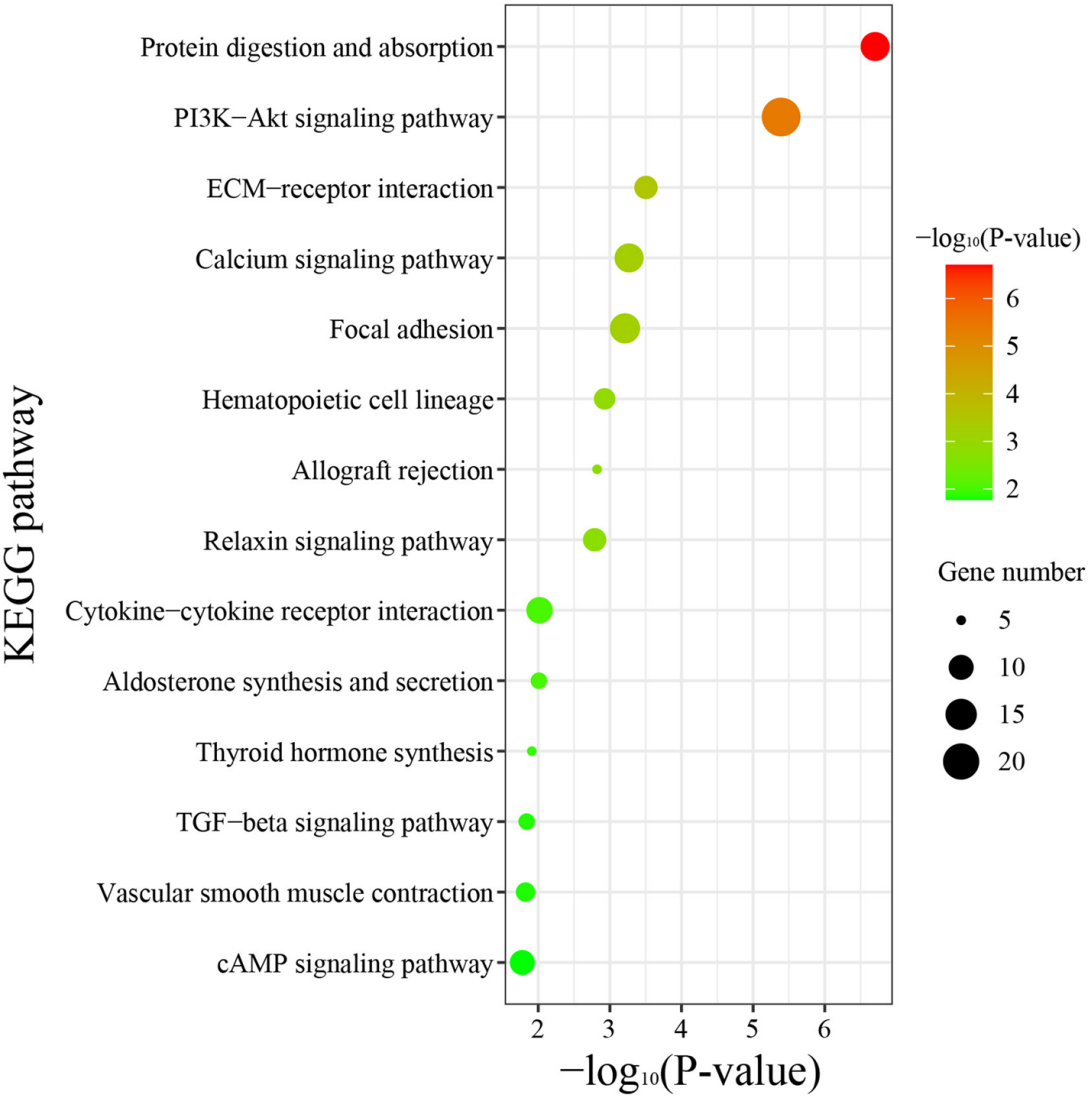
KEGG pathway analysis of the diferentially expressed genes (DEGs) between the large knob (L) and small knob (S) groups. The X-axis represents the larger the − log_10_ P value is, the more significant of enrichment of the DEGs in this pathway is. The Y-axis represents the name of pathway. The size and color of each bubble represent the amount of DEGs enriched in the pathway and enrichment signifcance, respectively (P adjust < 0.05).

To further elucidate the functional roles of DEGs in determining the knob size, we performed GO and KEGG pathway-enrichment analysis for the DEGs using the Goatools and KOBAS programs. The DEGs were categorized into three main GO categories, namely biological process, cellular component, and molecular function. 455 significantly enriched GO terms (Table S4) in 94 molecular functions, 52 cellular components, and 309 biological processes (P value < 0.05) were identified, and 30 overrepresented GO terms (P adjust < 0.05) were showed (Fig. 6). Then, the 415 DEGs were mapped to 210 KEGG pathways, 14 overrepresented pathways (P value < 0.05) were enriched, including thyroid hormone synthesis, TGF-beta signaling pathway, etc. (Fig. 7, Table S5). Among those pathways, *BMP5* and *DCN* were related to skeletal system development, *TSHR* and *ADCY3* were related to thyroid hormone secretion.

### Protein–protein interaction (PPI) analysis

**Figure 8:**
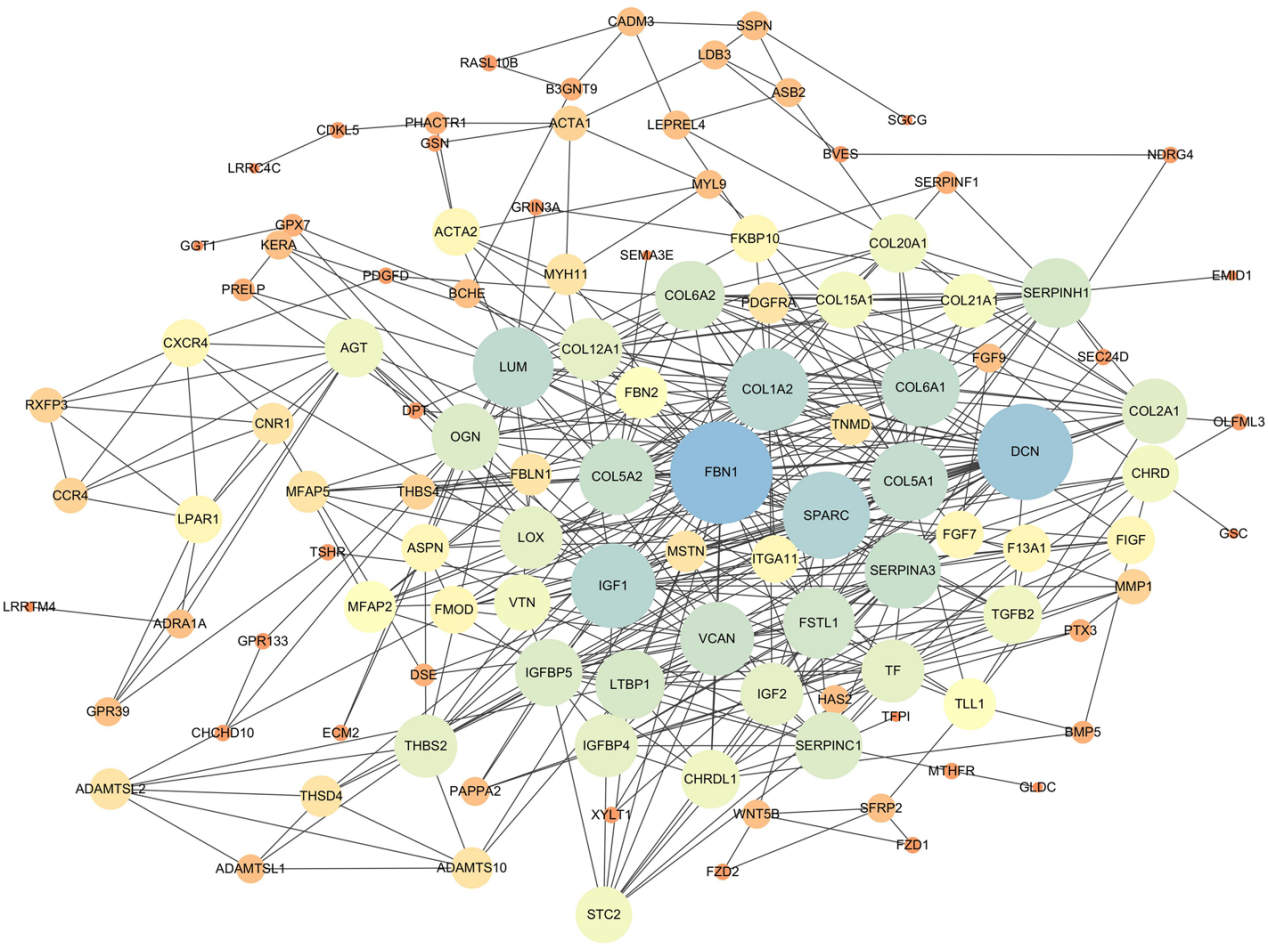
Protein–protein interaction network for the DEGs identified in this study. Nodes represent proteins, and edges represent interaction between proteins. The size of a node is proportional to its degree (degree is defined as the number of proteins that interact with the node). The color of a node is used to distinguish nodes with different degrees.

Fuethermore, based on the DEGs identified in this study, the PPI network was generated using the STRING program in Cytoscape^4^ software (Fig. 8). *FBN1* and *DCN* were in the center of the PPI network, which could be considered as the key gene associated with the knob-size phenotype. Besides, the *SPARC*, *IGF1*, *LUM*, and *COL1A2* genes were highly interconnected within subnet. Combined with the function and pathway of those genes, *DCN* gene was identified as one of hub genes to determine the knob-size phenotype.

### Validation of DEGs in geese with different knob sizes

**Figure 9:**
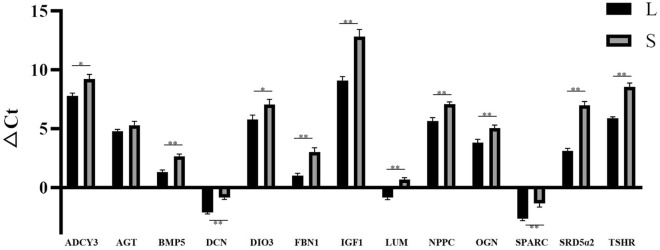
mRNA expression of the key 13 DEGs in large-knob geese and small-knob ones. Gene expression was characterized using RT-qPCR and was represented relative to β-actin. Mean △Ct values of the small-knob group served as calibrators. Vertical bars represent the mean ± SD (n = 3). Significant differences relative to controls are indicated with one (*P* < 0.05) or two (*P* < 0.01) asterisks. The black and gray bar were large-knob geese and small-knob geese, respectively.

To determine candidate genes associated with knob traits, the key DEGs were selected as the most differentially expressed genes and genes from relevant pathways for validation were selected, including 3 skeleton development related genes (*BMP5*, *NPPC*, *OGN*); 5 hormone-related genes (*AGT*, *DIO3*, *IGF1*, *SRD5α2*, *TSHR*); 5 protein synthesis related genes (*DCN*, *ADCY3*, *FBN1*, *LUM*, *SPARC*), and RT-qPCR analysis was conducted furtherly. The results showed their mRNA level was significantly high in the geese with large knob compared the small ones, which were in agreement with the RNA-seq data except *AGT* (Fig. 9).

## Discussion

In our work, the morphology, anatomy and histology of goose knob were observed. The knob included the integumentary outgrowth and frontal hump. Phenotypically, the knob in Yangzhou geese accentuated the facial contours. Many birds in the *Anseriformes* order showed the similar trait, such as *Melanitta*^1^ and *Cygnus olor*^5^. Anatomically, the frontal hump of the goose was formed by a combination of paired humps above the frontal-naso junction, which suggested that the knob from the inflated humps in the frontal area, rather than from the beak. The size of frontal hump was highly correlated with that of the knob, suggested that the knob phenotype was determined by the frontal hump. But not all knob size is closely related to the frontal hump in Chinese goose. Some goose knobs are soft and contain a mound of fleshy fat with tiny humps, such as Magang goose (Chinese goose, *Anser cygnoides*). In this study, we observed that the knobs and its frontal hump had great variations in terms of the different dimensions except the width of the frontal hump. The length and height of the frontal hump are well beyond the range of the frontal bone, but the growth of the frontal hump width is limited by the frontal bone width.

Histologically, our results also confirmed that significant differences occurred in knob skin between the large knobs and small ones. The knob skin contained some sublayer including the stratum corneum, stratum spinosum, and stratum reticular, and a greater thickness of the sublayer was observed in geese with large knobs than those in small knobs. The stratum reticular is rich in elastic fiber bundles and collagen fiber bundles. The thickness of the stratum reticular may directly affect the content of the elastin and collagen components in skin tissue^6,7^. Therefore, that large knobs with thicker stratum reticular might have higher elastin and collagen contents. Moreover, both skin and subcutaneous tissue of large knobs show dense bundles of collagen fibers and collagen fibers is the synthetic basis of bone formation. In birds, when ectopic transplantation of the ectodermal boundary, a series of molecular reactions was initiated, eventually changed the mesenchyma derived from the neural crest to form a repeated upper beak structure in quail^8^. Hence, the skin and subcutaneous tissue of knob is an important tissue, which is related to the ossification of frontal hump.

Also, the 415 differentially expressed genes were found between the large knobs and small ones through transcriptome profiling, and TGF-β signaling and thyroid hormone synthesis-signaling pathways were identified to determine knob-size phenotype. Furthermore, *BMP5*, *DCN*, *TSHR* and *ADCY3* were recognized to involve in the growth and development of knob. TGF-β signaling plays a key role in tissue homeostasis by dynamic regulating cellular processes including cell growth, migration, differentiation, epithelial-mesenchymal transition, and extracellular matrix remodeling. *BMP5*, as a key member of TGF-β signaling pathway, has been implicated in regulating multiple stages of bone development^9^*. BMP5* may serve patterning functions in cranial base development^10^. The ectopic expression of *BMP5* can induce specific craniofacial anomalies^11^. In our work, *BMP5* was upregulated in large knob, indicating that the expression of *BMP5* may promote the development of frontal hump in large knob. *DCN*, as the other memebers in TGF-β signaling pathway, is an essential growth factor for bone formation. *DCN* is a component of connective tissue. It binds to type I collagen fibrils and has a stronger affinity for collagen^12,13^. In our study, greater *DCN* expreesion was observed in large knob and *DCN* was considered as hub gene to determine the knob-size phenotype, which meant binding more collagen to increase osteogenesis. In addition, Thyroid hormone synthesis-signaling pathways is also important for skull bone growth^14^. In this study, thyroid hormone synthesis (including *TSHR* and *ADCY3*) also appeared to be crucial, as these genes showed significantly higher expression levels in the L group than in the S group. *ADCY3* is a membrane-associated enzyme and is induced during osteoclastogenesis^15^. *TSHR* can exert direct actions in cartilage and bone^16^. High expression of *TSHR* also increase the production of hyaluronic acid in skin and connective tissue^17^. Hyaluronic acid (a kind of glycosaminoglycan) have been shown to influence cell behavior and to play an important role in tissue development and repair^18^. Therefore, *ADCY3* and *TSHR* regulate bone development and knob formation by affecting thyroid hormone synthesis. Collectively, TGF-β signaling and thyroid hormone synthesis-signaling pathways, as well as *BMP5*, *DCN*, *TSHR* and *ADCY3* might participate in knob growth an development.

## Materials and methods

### Birds, measurement of knob size and samples collection

Approximately 500 380-day-old, healthy male Yangzhou geese were raised at Yangzhou Tiange Goose Industry Co., Ltd (Yangzhou, China). according to the farm’s standard practice. Measurement of knob adapted from Nicholas Horrocks (2010)^5^. Specifically, The length, height, and width of the knob were measured in millimeters for all the birds. The knob length (Kl) was measured between the most anterior part of the knob and the phenotypic boundary of knob (The junction of feather and integumentary outgrowth) (Fig. 1B). The knob width (Kw) was measured at the widest part of the knob (Fig. 1B). The knob height (Fhh) was measured between the fronto-nasal junction and the most dorsal part of the knob (Fig. 1B). Knob size was assessed by the product of knob length, width and height. The average product was about 38,000 ± 10,000 (mm^3^) and large knob size was defined as the product > 50,000 mm^3^, and small one was < 27,000 mm^3^. Also, six geese with large knob or small knob were selected, respectively, and then the birds were humanely euthanized (electronarcosis followed by bleeding). The apical integumentary outgrowth (including skin and subcutaneous connective tissue) tissue of the knob was collected and immediately placed in liquid nitrogen and stored in freezer (− 80 °C) for subsequent RNA isolation. Besides, some integumentary outgrowth tissues were taken for paraffin sections, and the remaining parts were used for skull taxidermy.

### Skull specimen preparation and anatomical measurements

All skulls were put in a moist and warm plastic bag to accelerate decomposition and skeletonization. Each individual skull was classified with a corresponding label (consistent with phenotype, histology and transcriptome), the skulls were collected after 4 months, and then they were cleaned with running water and ethyl alcohol. A toothbrush was used to remove the rotten muscle residues. Finally, the skulls were dried naturally. The frontal hump anatomical measurements adapted from Delphine Angst (2020)^19^. Specifically, The measurement of frontal hump corresponded to where the knob was measured. The length, height, and width of the frontal hump were measured in millimeters for all the specimens. The frontal hump length (Fhl) was measured between the most anterior part of the suture of the two frontals and the phenotypic boundary of knob (mark here with scalpel in advance) (Fig. 1C). The frontal hump width (Fhw) was measured at the widest part of the hump (Fig. 1C). The frontal hump height (Fhh) was measured between the fronto-nasal junction and the most dorsal part of the frontal hump (Fig. 1C).

### Histological detection

The skin samples were fixed in 4% paraformaldehyde. After 24 h, the samples were placed in an embedding cassette and rinsed with running water (to remove the fixative from the tissue) for 30 min, and the samples were dehydrated in a graded ethanol series. A JB-P5 tissue-embedding machine (Wuhan Junjie Electronics Co., Ltd., Wuhan, China) was used for paraffin embedding at 70 °C. The paraffin blocks were cut (RM2016, Germany) along the horizontal axis into 3 μm-thick sections and stained with hematoxylin and eosin (HE) according to standard protocols. The knob skin was examined under an upright optical microscope (Nikon, Tokyo, Japan), and image acquisition and analysis were performed with a DS-U3 Imaging system (Nikon, Tokyo, Japan).

### RNA sequencing (RNA-seq) and bioinformatics analysis

#### RNA extraction

Total RNA was extracted from integumentary outgrowth of geese knob in the large (L) and small (S) groups. Using the TRIzol^®^ Reagent (Animal RNA Purification Reagent for animal tissues; Invitrogen) following the manufacturer’s recommendations, and genomic DNA was removed using DNase I (TaKaRa). Total RNA purity,concentration and integrity of each samples were estimated by using a Nanodrop 2000 instrument (Thermo Scientific, Wilmington, DE, USA) and an Agilent 2100 Bioanalyzer (Agilent Technologies, Santa Clara, CA, USA), and only high-quality RNA samples were used to construct the sequencing library^20^.

#### Library preparation for sequencing

An RNA sequencing library was prepared using the TruSeq™ RNA Sample Preparation Kit from Illumina (San Diego, CA) following the manufacturer’s recommendations. In brief, mRNA was purified via Poly (A) selection with oligo(dT) cellulose. and then fragmented in fragmentation buffer. Continually, double-stranded cDNA was synthesized using a Super Script Double-Stranded cDNA Synthesis Kit (Invitrogen, CA) with random hexamer primers (Illumina). The synthesized cDNA was subjected to end-repaired, phosphorylation and the A-tailed according to library-construction protocol of Illumina. Libraries were size-selected for cDNA target fragments of 300 base pairs (bp) on a 2% low-range ultra agarose gel, followed by PCR amplification. Finally, the amplified fragments were sequenced with an Illumina HiSeq Xten/NovaSeq 6000 sequencer according to the manufacturer’s instructions^21^. The sequencing data have been deposited in the NCBI Sequence Read Archive (SRA), and are accessible through the accession number PRJNA727442.

#### Read mapping

The raw paired-end reads were trimmed and quality-controlled using the default parameters of the SeqPrep (https://github.com/jstjohn/SeqPrep) and sickle (https://github.com/najoshi/sickle) software programs. Clean reads were then separately aligned to the reference genome (*Anser cygnoides*, GCF_000971095.1, https://www.ncbi.nlm.nih.gov/genome/?term=Anser cygnoides); in orientation mode, using HISAT2 software (http://ccb.jhu.edu/software/hisat2/index.shtml)^22^. The mapped reads of each sample were assembled using a reference-based approach with StringTie software (https://ccb.jhu.edu/software/stringtie/index.shtml?t=example)^23^.

#### Differential expression analysis and functional enrichment

To identify differentially expressed genes (DEGs) between two different samples, the expression levels of each transcript were calculated using the transcripts-per-million reads (TPM) method. RSEM software (http://deweylab.biostat.wisc.edu/rsem/)^24^was used to quantify gene abundances. Essentially, differential-expression analysis was performed using the DESeq2 program^25^, where an adjusted P value (P adjust) of < 0.05, and DEGs with absolute fold-changes (FCs) of > 2, a P adjust value of < 0.05 (DESeq2), and a P adjust value of < 0.001 (DESeq2) were considered DEGs with statistical significance. Moreover, functional-enrichment analysis was performed with the Gene Ontology (GO) and Kyoto Encyclopedia of Genes and Genomes (KEGG) databases^26^ to determine which DEGs were significantly enriched for GO terms and metabolic pathways at a Bonferroni-corrected P value of < 0.05, when compared with the whole-transcriptome background. GO functional enrichment and KEGG-pathway analysis were performed using the Goatools (https://github.com/tanghaibao/Goatools) and KOBAS (http://kobas.cbi.pku.edu.cn/home.do) software programs^27^.

#### Protein–protein interaction network analysis

Based on STRING database (Version: 11.0), the protein–protein interaction network was analyzed for DEGs and further investigated the interaction between DEGs. Cystoscope (Version: 3.6.1) was used to visualize the protein–protein interaction network to find out key genes.

### Real-time quantitative PCR (RT-qPCR)

DEGs from relevant pathways or the most differentially expressed DEGs were selected for validation by RNA-Seq. Total RNA extracted from each integumentary outgrowth of goose knob was subjected to RT-qPCR analysis. Single-strand cDNA was synthesized using approximately 5 mg of total RNA and a Revertaid™ First Strand cDNA Synthesis Kit (Fermentas, Fermentas China Co., Ltd., China), and the resulting cDNA was diluted five-fold. RT-qPCR analysis was performed using SYBR Green Real-time PCR Master Mix (TOYOBO, Osaka, Japan) and an ABI 7500 Real-Time PCR System (Applied Biosystems, Foster City, CA). Each 10 µL reaction contained 5 µL SYBR Green Real time PCR Master Mix, 0.4 μL forward primer (10 μM), 0.4 μL reverse primer (10 μM), 2 µL cDNA, and 2.2 µL distilled water. The RT-qPCR program was 50 °C for 2 min; 95 °C for 2 min; followed by 40 cycles of 95 °C for 15 s and 40 cycles of 60 °C for 15 s; 95 °C for 15 s; 60 °C for 1 min; 95 °C for 15 s. Mean △Ct values of the small-knob group served as calibrators, and β-actin serving as an internal reference gene. The sequences of the primers used to amplify each of the 13 mRNAs are shown in Table S1.

### Ethics approval

Our study was carried out in compliance with the ARRIVE (Animal Research: Reporting of In Vivo Experiments) guidelines. All animal experiments were approved by with the Institutional Animal Care and Use Committee of Yangzhou University (approval number: 151-2018). Procedures were strictly performed in accordance with the Regulations for the Administration of Affairs Concerning Experimental Animals (Yangzhou University, China, 2012) and the Standards for the Administration of Experimental Practices (Jiangsu, China, 2008). We also confirm that we have done all efforts to minimize the suffering of animals.

## Supplementary Information

Supplementary Information 1.
