# Supplementary material for: Identifying molecular pathways and candidate genes associated with knob traits by transcriptome analysis in the goose (Anser cygnoides)
Source: Sci Rep. 2021 Jun 7;11:11978. doi: 10.1038/s41598-021-91269-1 (PMC8184827; doi:10.1038/s41598-021-91269-1)
Supplement: Supplementary file 1 — Supplementary Information 1. [file 41598_2021_91269_MOESM1_ESM.docx]

| Table S1 Primer sequence | |
| --- | --- |
| Primer name | Primer sequence |
| ADCY3-F | CAACGTGGCCATCTACCTGT |
| ADCY3-R | CTTCAGCATCTCATCCGCCA |
| AGT-F | CTTGGCAGAATAACAAACCC |
| AGT-R | GTATTTCAAACACTACCTGCT |
| BMP5-F | AGAGCAGAGGGATACGGA |
| BMP5-R | GCTGGCTAAGGGAGGATT |
| DCN-F | CTAGTGGGCTGGGTGAA |
| DCN-R | GGTGTTGTATCCAAGAGGG |
| DIO3-F | ACAACATCCCCAAGCACCAGT |
| DIO3-R | TGCCTCCCTGGTACATCACCT |
| FBN1-F | GGACAGAAGTGGTGGTAAC |
| FBN1-R | GGACAGAAGTGGTGGTAAC |
| IGF1-F | TTGAAATATATGGCTAACAGT |
| IGF1-R | TCAGTATTCTGTCTAGCCTA |
| LUM-F | ACAGACCTACAGTGGCTGAT |
| LUM-R | CAGAGTTTTGGGGAGTGG |
| NPPC-F | TAGCATATGTTGAGACCCCTG |
| NPPC-R | GTGCCACTTATGTATGTCAGT |
| OGN-F | CAGCCTGACTCTGCTAAGGA |
| OGN-R | TTTACAACAGGAGTTATTGGCAT |
| SPARC-F | GAGGGAACCAAGAAGGG |
| SPARC-R | CGCTCGTACAGGGTGAT |
| SRD5A2-F | CAATAGCTGGTAACAGTACTCC |
| SRD5A2-R | TAAGACAGCTATGATGCCGAA |

| Table S2. The sequence read statistics | | | | | | | | |
| --- | --- | --- | --- | --- | --- | --- | --- | --- |
| Sample | Raw reads | Raw bases | Clean reads | Clean bases | Error rate (%) | Q20(%) | Q30(%) | GC content (%) |
| L1 | 53658592 | 8102447392 | 52943186 | 7758258817 | 0.0244 | 98.17 | 94.8 | 48.98 |
| L2 | 51379352 | 7758282152 | 50742622 | 7490278171 | 0.0244 | 98.21 | 94.88 | 49.51 |
| L3 | 46685966 | 7049580866 | 46164770 | 6806104391 | 0.0244 | 98.2 | 94.83 | 48.74 |
| S1 | 49859158 | 7528732858 | 49263786 | 7208764222 | 0.0245 | 98.16 | 94.74 | 48.38 |
| S2 | 47311094 | 7143975194 | 46696684 | 6890336068 | 0.0246 | 98.11 | 94.62 | 48.61 |
| S3 | 51924682 | 7840626982 | 51191204 | 7411880601 | 0.0245 | 98.16 | 94.77 | 49.37 |

(1) Sample: Name of the Sample; (2) Raw reads: the total number of items in the original sequencing data;(3) Raw bases: The total number of Raw reads multiplied by the length of reads.(4) Clean reads: the total number of items in sequencing data after quality control;(5) Clean bases: the total number of Clean reads multiplied by the length of reads after quality control; (6) Error rate (%) : the average Error rate of sequencing bases corresponding to quality control data is generally less than 0.1%;(7) Q20 (%) and Q30 (%) : the quality of sequencing data after quality control is evaluated. Q20 and Q30 refer to the percentage of bases with sequencing quality above 99% and 99.9% in the total base, respectively. Generally, Q20 is above 85% and Q30 is above 80%. (8) GC Content (%) : The percentage of the sum of G and C bases corresponding to the quality control data in the total base

Table S3. Differentially expressed mRNAs

| Gene_id | Gene name | Gene description | FC(L/S) | Log2FC(L/S) | P-value | P-adjust | Significant | Regulate | S1 | S2 | S3 | L1 | L2 | L3 | S | L |
| --- | --- | --- | --- | --- | --- | --- | --- | --- | --- | --- | --- | --- | --- | --- | --- | --- |
| gene11550 | NPPC | natriuretic peptide C | 119.12 | 6.896277243 | 1.61855E-13 | 7.01441E-11 | yes | up | 0.9 | 1.57 | 4.46 | 350.72 | 326.4 | 65.96 | 2.31 | 247.6933333 |
| gene2546 | ARMC3 | armadillo repeat containing 3, transcript variant X3 | 44.456 | 5.474298673 | 0.000857088 | 0.024814403 | yes | up | 0 | 0 | 0.02 | 0.59 | 0.06 | 0.26 | 0.006666667 | 0.303333333 |
| gene11568 | LOC106040101 | somatostatin-2-like | 39.703 | 5.311194117 | 1.67806E-05 | 0.001003066 | yes | up | 1.66 | 2.31 | 1.59 | 110.93 | 2.71 | 91.42 | 1.853333333 | 68.35333333 |
| gene8039 | C1QTNF3 | C1q and tumor necrosis factor related protein 3, transcript variant X1 | 38.024 | 5.248853901 | 2.92103E-06 | 0.000232809 | yes | up | 0.15 | 0.11 | 1.58 | 21.8 | 15.85 | 27.24 | 0.613333333 | 21.63 |
| gene8537 | KERA | keratocan, transcript variant X2 | 31.534 | 4.978852563 | 5.2208E-39 | 3.6201E-35 | yes | up | 0.5 | 0.49 | 0.39 | 12.61 | 22.86 | 9.83 | 0.46 | 15.1 |
| gene17018 | ADRA1A | adrenoceptor alpha 1A, transcript variant X2 | 27.007 | 4.755273855 | 0.001495424 | 0.03784404 | yes | up | 0 | 0.03 | 0.02 | 0.07 | 0.82 | 0.32 | 0.016666667 | 0.403333333 |
| gene10326 | NGB | neuroglobin | 25.9 | 4.694875029 | 5.83836E-20 | 7.36058E-17 | yes | up | 0.14 | 0.12 | 0.2 | 3.46 | 2.82 | 5.24 | 0.153333333 | 3.84 |
| gene5050 | LOC106033090 | uncharacterized LOC106033090, transcript variant X2 | 25.606 | 4.6784066 | 2.30679E-11 | 5.92418E-09 | yes | up | 0.04 | 0.05 | 0.03 | 1.3 | 0.64 | 0.85 | 0.04 | 0.93 |
| gene12347 | GPR12 | G protein-coupled receptor 12 | 25.375 | 4.665343788 | 0.000136312 | 0.005626097 | yes | up | 0.01 | 0.01 | 0.01 | 0.19 | 0.45 | 0.02 | 0.01 | 0.22 |
| gene6376 | LOC106034513 | uncharacterized LOC106034513, transcript variant X2 | 19.957 | 4.31883622 | 2.8674E-13 | 1.13614E-10 | yes | up | 0.02 | 0.03 | 0.03 | 0.35 | 0.37 | 0.45 | 0.026666667 | 0.39 |
| gene17073 | LOC106046313 | chromosome unknown open reading frame, human C16orf89, transcript variant X1 | 18.719 | 4.226447498 | 1.01239E-11 | 2.69997E-09 | yes | up | 0.3 | 0.3 | 0.13 | 2.58 | 7.74 | 2.11 | 0.243333333 | 4.143333333 |
| gene1942 | SRD5A2 | steroid-5-alpha-reductase, alpha polypeptide 2 (3-oxo-5 alpha-steroid delta 4-dehydrogenase alpha 2), transcript variant X1 | 18.245 | 4.18938991 | 1.16171E-95 | 1.61106E-91 | yes | up | 4.64 | 4.17 | 5.63 | 90.81 | 68.58 | 71.29 | 4.813333333 | 76.89333333 |
| gene7691 | GABBR2 | gamma-aminobutyric acid (GABA) B receptor, 2, transcript variant X1 | 17.541 | 4.132645874 | 4.11503E-10 | 8.15246E-08 | yes | up | 8.48 | 0.19 | 0.08 | 2.94 | 5.7 | 0.9 | 2.916666667 | 3.18 |
| gene17025 | LRRTM4 | leucine rich repeat transmembrane neuronal 4, transcript variant X4 | 13.631 | 3.768866797 | 2.91359E-06 | 0.000232809 | yes | up | 16.03 | 0 | 4.76 | 28.98 | 1.16 | 1 | 6.93 | 10.38 |
| gene4351 | SFRP2 | secreted frizzled-related protein 2 | 13.37 | 3.740955679 | 6.52836E-13 | 2.32142E-10 | yes | up | 33.42 | 62.86 | 50.96 | 976.85 | 173.17 | 661.68 | 49.08 | 603.9 |
| gene359 | LOC106031687 | protein NDNF-like | 13.146 | 3.716528594 | 0.001874438 | 0.044895859 | yes | up | 0.06 | 0 | 0.06 | 0.54 | 0.14 | 0.71 | 0.04 | 0.463333333 |
| gene13579 | BRINP3 | bone morphogenetic protein/retinoic acid inducible neural-specific 3, transcript variant X1 | 12.31 | 3.621761779 | 1.71164E-08 | 2.32716E-06 | yes | up | 0.07 | 0.15 | 0.16 | 1.7 | 1.43 | 0.75 | 0.126666667 | 1.293333333 |
| gene4262 | C1QTNF8 | C1q and tumor necrosis factor related protein 8, transcript variant X2 | 12.196 | 3.608278878 | 7.79725E-05 | 0.003604408 | yes | up | 0.99 | 1.1 | 6.82 | 26.25 | 42.84 | 32.1 | 2.97 | 33.73 |
| gene5769 | LUZP2 | leucine zipper protein 2, transcript variant X1 | 11.275 | 3.495065051 | 4.08104E-05 | 0.002118764 | yes | up | 0.16 | 0.14 | 0.1 | 2.18 | 1.53 | 0.31 | 0.133333333 | 1.34 |
| gene1911 | LOC106049170 | uncharacterized LOC106049170, transcript variant X3 | 11.129 | 3.476275367 | 0.001807386 | 0.043667759 | yes | up | 0.11 | 0 | 0.16 | 1.52 | 0.53 | 0.42 | 0.09 | 0.823333333 |
| gene13200 | LOC106042047 | uncharacterized LOC106042047 | 10.69 | 3.418148467 | 0.002079367 | 0.047742828 | yes | up | 0.02 | 0.04 | 0.02 | 0.21 | 0.39 | 0.14 | 0.026666667 | 0.246666667 |
| gene18709 | MYO3A | myosin IIIA | 10.53 | 3.396415583 | 0.000606198 | 0.018934138 | yes | up | 0.08 | 0.04 | 0.03 | 0.28 | 0.06 | 1.07 | 0.05 | 0.47 |
| gene8959 | TNMD | tenomodulin | 10.081 | 3.333628075 | 6.25988E-05 | 0.003021333 | yes | up | 0.07 | 0.17 | 0.67 | 2.81 | 1.71 | 4.01 | 0.303333333 | 2.843333333 |
| gene11699 | FGF9 | fibroblast growth factor 9 | 9.852 | 3.300387925 | 0.001364097 | 0.035293468 | yes | up | 0.08 | 0.04 | 0.24 | 1.26 | 1.89 | 0.22 | 0.12 | 1.123333333 |
| gene6163 | CNTNAP5 | contactin associated protein-like 5 | 9.472 | 3.24374334 | 8.87413E-06 | 0.000567126 | yes | up | 0.07 | 0.01 | 0.04 | 0.24 | 0.64 | 0.19 | 0.04 | 0.356666667 |
| gene4061 | SLITRK3 | SLIT and NTRK-like family, member 3 | 9.367 | 3.227566002 | 8.12656E-08 | 9.71545E-06 | yes | up | 0.21 | 0.24 | 0.14 | 1.8 | 2.1 | 1.12 | 0.196666667 | 1.673333333 |
| gene7366 | ESM1 | endothelial cell-specific molecule 1 | 9.029 | 3.174617823 | 8.33195E-05 | 0.003780562 | yes | up | 0.21 | 0.29 | 0.24 | 0.59 | 4.56 | 0.91 | 0.246666667 | 2.02 |
| gene20 | KCNIP2 | Kv channel interacting protein 2, transcript variant X1 | 8.995 | 3.169141294 | 2.56172E-08 | 3.38342E-06 | yes | up | 0.45 | 0.44 | 0.09 | 3.58 | 4.34 | 1.87 | 0.326666667 | 3.263333333 |
| gene19160 | LOC106048599 | homeobox protein unc-4 homolog | 8.986 | 3.16764121 | 3.5422E-06 | 0.000269908 | yes | up | 0.11 | 0.03 | 0.19 | 0.51 | 0.83 | 1.34 | 0.11 | 0.893333333 |
| gene3935 | NALCN | sodium leak channel, non selective, transcript variant X1 | 8.901 | 3.15396995 | 8.39658E-06 | 0.000541599 | yes | up | 0.06 | 0.07 | 0.06 | 0.6 | 0.72 | 0.16 | 0.063333333 | 0.493333333 |
| gene7043 | NIPAL2 | NIPA-like domain containing 2 | 8.891 | 3.152376961 | 3.36034E-22 | 5.17791E-19 | yes | up | 0.49 | 1.31 | 1.12 | 8.19 | 4.08 | 3.63 | 0.973333333 | 5.3 |
| gene7326 | SEMA3E | sema domain, immunoglobulin domain (Ig), short basic domain, secreted, (semaphorin) 3E, transcript variant X1 | 8.722 | 3.124690015 | 5.08068E-05 | 0.002495284 | yes | up | 0.11 | 0.07 | 0.25 | 0.33 | 2.37 | 0.76 | 0.143333333 | 1.153333333 |
| gene63 | LOC106037964 | uncharacterized LOC106037964, transcript variant X1 | 8.706 | 3.12199349 | 6.51866E-09 | 9.51587E-07 | yes | up | 0.21 | 0.12 | 0.4 | 1.62 | 1.96 | 0.86 | 0.243333333 | 1.48 |
| gene8042 | RXFP3 | relaxin/insulin-like family peptide receptor 3 | 8.596 | 3.103660376 | 2.90512E-07 | 3.12311E-05 | yes | up | 0.33 | 0.91 | 0.22 | 6.53 | 2.15 | 2.83 | 0.486666667 | 3.836666667 |
| gene14572 | PTX3 | pentraxin 3, long | 8.462 | 3.080928135 | 0.000828129 | 0.024331548 | yes | up | 8.35 | 9.06 | 2.15 | 19.02 | 27.27 | 103.62 | 6.52 | 49.97 |
| gene18194 | SLN | sarcolipin | 8.188 | 3.033490894 | 0.002037049 | 0.047404387 | yes | up | 0.55 | 0.88 | 3.81 | 0.96 | 21.82 | 17.47 | 1.746666667 | 13.41666667 |
| gene17364 | FAM198A | family with sequence similarity 198, member A | 8.139 | 3.024821497 | 7.27364E-28 | 2.01742E-24 | yes | up | 5.21 | 3.41 | 2.78 | 27.48 | 32.69 | 24.52 | 3.8 | 28.23 |
| gene11263 | TENM4 | teneurin transmembrane protein 4, transcript variant X4 | 8.124 | 3.022272426 | 5.45315E-21 | 7.56243E-18 | yes | up | 0.77 | 1.05 | 0.71 | 7.2 | 7.72 | 3.91 | 0.843333333 | 6.276666667 |
| gene10044 | TRPC5 | transient receptor potential cation channel, subfamily C, member 5 | 7.8 | 2.963407576 | 0.000380603 | 0.012905128 | yes | up | 0.06 | 0.03 | 0.03 | 0.09 | 0.56 | 0.22 | 0.04 | 0.29 |
| gene5718 | CRABP1 | cellular retinoic acid binding protein 1, transcript variant X2 | 7.785 | 2.960754958 | 9.60502E-07 | 8.5937E-05 | yes | up | 3.32 | 13.18 | 5.4 | 98.33 | 24.71 | 34.18 | 7.3 | 52.40666667 |
| gene15495 | TSHR | thyroid stimulating hormone receptor | 7.61 | 2.927949152 | 7.35427E-09 | 1.06239E-06 | yes | up | 0.25 | 0.12 | 0.47 | 2.22 | 1.34 | 2.28 | 0.28 | 1.946666667 |
| gene12661 | HSPB1 | heat shock 27kDa protein 1 | 7.341 | 2.875986972 | 0.001149532 | 0.030835035 | yes | up | 3.39 | 0.67 | 7.15 | 4.99 | 44.06 | 32.01 | 3.736666667 | 27.02 |
| gene6373 | UNC80 | unc-80 homolog (C. elegans) | 7.223 | 2.852517963 | 5.03883E-07 | 4.95592E-05 | yes | up | 1.67 | 2.36 | 0.74 | 5.87 | 20.47 | 4.91 | 1.59 | 10.41666667 |
| gene15134 | SLC24A2 | solute carrier family 24 (sodium/potassium/calcium exchanger), member 2, transcript variant X1 | 6.924 | 2.791663974 | 2.50528E-05 | 0.001406607 | yes | up | 0.2 | 0.17 | 0.03 | 0.5 | 1.46 | 0.58 | 0.133333333 | 0.846666667 |
| gene6378 | FRZB | frizzled-related protein | 6.883 | 2.782986709 | 1.45763E-26 | 3.36907E-23 | yes | up | 12.06 | 8.49 | 13.8 | 80.05 | 54.47 | 80.83 | 11.45 | 71.78333333 |
| gene10626 | TF | transferrin, transcript variant X1 | 6.84 | 2.773980335 | 1.29319E-12 | 4.48349E-10 | yes | up | 408.02 | 698.59 | 927.06 | 3733.89 | 2726.74 | 6264.54 | 677.89 | 4241.723333 |
| gene7519 | IGF1 | insulin-like growth factor 1 (somatomedin C) | 6.786 | 2.762515693 | 3.03164E-06 | 0.000238879 | yes | up | 2.35 | 6.76 | 1.05 | 31.95 | 19.53 | 12.41 | 3.386666667 | 21.29666667 |
| gene14736 | LOC106043734 | uncharacterized protein K02A2.6-like | 6.669 | 2.737479777 | 0.000906847 | 0.0257708 | yes | up | 0.61 | 0.06 | 0.63 | 4.61 | 1.05 | 2.54 | 0.433333333 | 2.733333333 |
| gene10047 | DCX | doublecortin, transcript variant X5 | 6.586 | 2.719295244 | 2.1736E-07 | 2.3735E-05 | yes | up | 0.48 | 2.1 | 0.41 | 3.45 | 2.64 | 4.31 | 0.996666667 | 3.466666667 |
| gene12680 | COL26A1 | collagen, type XXVI, alpha 1, transcript variant X1 | 6.585 | 2.71918146 | 4.47768E-16 | 3.10482E-13 | yes | up | 0.9 | 0.74 | 1.15 | 3.5 | 6.78 | 6.3 | 0.93 | 5.526666667 |
| gene10693 | LOC106039183 | glypican-5-like, transcript variant X1 | 6.502 | 2.700893831 | 9.23451E-29 | 3.20161E-25 | yes | up | 2.33 | 2.59 | 2.43 | 10.24 | 17.17 | 17.47 | 2.45 | 14.96 |
| gene3084 | RERGL | RERG/RAS-like | 6.492 | 2.698773417 | 0.001527065 | 0.038364741 | yes | up | 0.09 | 0.24 | 0.06 | 0.4 | 1.61 | 0.28 | 0.13 | 0.763333333 |
| gene6377 | LOC106034516 | uncharacterized LOC106034516 | 6.246 | 2.642845845 | 1.09766E-19 | 1.17095E-16 | yes | up | 3.14 | 3.01 | 3.98 | 23.77 | 13.6 | 20.52 | 3.376666667 | 19.29666667 |
| gene11556 | DRAXIN | dorsal inhibitory axon guidance protein, transcript variant X8 | 6.037 | 2.593753025 | 5.54393E-08 | 6.86457E-06 | yes | up | 1.89 | 4.17 | 4.88 | 35.28 | 14.29 | 14.37 | 3.646666667 | 21.31333333 |
| gene7329 | SEMA3D | sema domain, immunoglobulin domain (Ig), short basic domain, secreted, (semaphorin) 3D, transcript variant X2 | 6.009 | 2.587183397 | 4.61489E-06 | 0.00033862 | yes | up | 5.14 | 1.93 | 2.57 | 8.21 | 34.99 | 9.38 | 3.213333333 | 17.52666667 |
| gene639 | C1QTNF7 | C1q and tumor necrosis factor related protein 7, transcript variant X1 | 5.975 | 2.578976383 | 1.59803E-15 | 9.23396E-13 | yes | up | 2.05 | 1.35 | 1.49 | 10.53 | 6.63 | 9.8 | 1.63 | 8.986666667 |
| gene3518 | LRRC75B | leucine rich repeat containing 75B | 5.933 | 2.568654845 | 3.91141E-09 | 6.09476E-07 | yes | up | 3.12 | 5.65 | 2.35 | 29.19 | 12.27 | 19.07 | 3.706666667 | 20.17666667 |
| gene6992 | ENPP6 | ectonucleotide pyrophosphatase/phosphodiesterase 6 | 5.889 | 2.557966545 | 5.94121E-11 | 1.42056E-08 | yes | up | 1.82 | 2.54 | 2.12 | 18.25 | 10.58 | 6.19 | 2.16 | 11.67333333 |
| gene6408 | COL3A1 | collagen, type III, alpha 1 | 5.797 | 2.53533015 | 5.48721E-18 | 4.75604E-15 | yes | up | 592.67 | 371.17 | 292.19 | 1861.43 | 2593.71 | 2117.19 | 418.6766667 | 2190.776667 |
| gene3089 | LMO3 | LIM domain only 3 (rhombotin-like 2), transcript variant X1 | 5.766 | 2.527536319 | 7.86602E-07 | 7.17671E-05 | yes | up | 15.13 | 0.47 | 0.38 | 2.08 | 3.55 | 1.74 | 5.326666667 | 2.456666667 |
| gene12906 | CAP2 | CAP, adenylate cyclase-associated protein, 2 (yeast) | 5.71 | 2.513532463 | 8.12131E-06 | 0.000528762 | yes | up | 1.29 | 1.54 | 2.9 | 3.6 | 18.02 | 8.31 | 1.91 | 9.976666667 |
| gene16140 | ADAMTS19 | ADAM metallopeptidase with thrombospondin type 1 motif, 19 | 5.689 | 2.508194729 | 1.97506E-13 | 8.30006E-11 | yes | up | 0.49 | 0.41 | 0.41 | 2.25 | 1.97 | 1.91 | 0.436666667 | 2.043333333 |
| gene3517 | GGT1 | gamma-glutamyltransferase 1, transcript variant X1 | 5.67 | 2.503375733 | 1.21032E-09 | 2.20851E-07 | yes | up | 0.71 | 0.69 | 0.35 | 4.02 | 2.57 | 6.95 | 0.583333333 | 4.513333333 |
| gene10893 | BRDT | bromodomain, testis-specific, transcript variant X1 | 5.667 | 2.502507903 | 0.002002129 | 0.046980582 | yes | up | 0.11 | 0.04 | 0.08 | 0.53 | 0.21 | 0.33 | 0.076666667 | 0.356666667 |
| gene5031 | LOC106033075 | uncharacterized LOC106033075, transcript variant X1 | 5.663 | 2.50152553 | 0.000801999 | 0.023765222 | yes | up | 0.35 | 0.21 | 0.38 | 1.43 | 3.29 | 1.09 | 0.313333333 | 1.936666667 |
| gene19081 | MUC1 | mucin 1, cell surface associated | 5.559 | 2.474714911 | 6.22955E-06 | 0.000425573 | yes | up | 0.37 | 0.42 | 0.54 | 2.45 | 1.66 | 2.72 | 0.443333333 | 2.276666667 |
| gene16635 | OPTC | opticin, transcript variant X1 | 5.548 | 2.471932079 | 0.001016664 | 0.028198201 | yes | up | 0.11 | 0.44 | 0.22 | 1.12 | 0.95 | 1.25 | 0.256666667 | 1.106666667 |
| gene7608 | C1QTNF6 | C1q and tumor necrosis factor related protein 6 | 5.539 | 2.469534389 | 4.10112E-09 | 6.31937E-07 | yes | up | 3.88 | 3.23 | 3.35 | 12.13 | 30.55 | 10.07 | 3.486666667 | 17.58333333 |
| gene12317 | CALN1 | calneuron 1, transcript variant X1 | 5.516 | 2.463695952 | 0.001708954 | 0.041748298 | yes | up | 0.1 | 0.37 | 0.25 | 0.63 | 1.47 | 1.42 | 0.24 | 1.173333333 |
| gene10022 | LOC106038521 | wiskott-Aldrich syndrome protein family member 3-like | 5.476 | 2.453065047 | 2.4987E-10 | 5.17194E-08 | yes | up | 0.66 | 0.61 | 0.24 | 2.45 | 2.79 | 2.27 | 0.503333333 | 2.503333333 |
| gene4663 | LOC106032622 | blood vessel epicardial substance-like | 5.415 | 2.43695647 | 2.61214E-09 | 4.47224E-07 | yes | up | 1.33 | 1.43 | 1.98 | 3.27 | 6.12 | 7.25 | 1.58 | 5.546666667 |
| gene9326 | FIGF | c-fos induced growth factor (vascular endothelial growth factor D) | 5.325 | 2.412716534 | 3.59341E-06 | 0.000272314 | yes | up | 7.66 | 10.92 | 3.51 | 26.5 | 63.28 | 17.84 | 7.363333333 | 35.87333333 |
| gene19080 | THBS3 | thrombospondin 3 | 5.298 | 2.405380223 | 4.78489E-36 | 2.21189E-32 | yes | up | 4.94 | 4.29 | 5.28 | 21.88 | 26.21 | 22.24 | 4.836666667 | 23.44333333 |
| gene7164 | CNR1 | cannabinoid receptor 1 (brain), transcript variant X1 | 5.295 | 2.404527536 | 3.86681E-18 | 3.575E-15 | yes | up | 2.42 | 2.45 | 1.9 | 11.83 | 13.4 | 7.54 | 2.256666667 | 10.92333333 |
| gene8478 | VILL | villin-like, transcript variant X1 | 5.188 | 2.375049064 | 1.15403E-06 | 0.000100026 | yes | up | 0.42 | 0.28 | 0.79 | 2.07 | 3.45 | 1.72 | 0.496666667 | 2.413333333 |
| gene4062 | BCHE | butyrylcholinesterase, transcript variant X1 | 5.075 | 2.343316832 | 1.01872E-08 | 1.42703E-06 | yes | up | 3.95 | 6.45 | 2.55 | 15.23 | 31.07 | 14.58 | 4.316666667 | 20.29333333 |
| gene1971 | LOC106029463 | cytochrome P450 1B1 | 5.048 | 2.335700593 | 1.16274E-08 | 1.61249E-06 | yes | up | 4.27 | 5.87 | 2.66 | 17.38 | 11.8 | 29.62 | 4.266666667 | 19.6 |
| gene538 | NAT8L | N-acetyltransferase 8-like (GCN5-related, putative) | 5.024 | 2.328921792 | 0.000862284 | 0.024912829 | yes | up | 0.22 | 0.12 | 0.02 | 0.57 | 0.65 | 0.42 | 0.12 | 0.546666667 |
| gene20121 | LOC106049626 | histone H2B 5 | 4.927 | 2.300591957 | 8.88191E-05 | 0.003947894 | yes | up | 7.41 | 7.27 | 25.76 | 50.77 | 33.91 | 103.22 | 13.48 | 62.63333333 |
| gene7753 | FAM69C | family with sequence similarity 69, member C | 4.898 | 2.292263487 | 6.50649E-15 | 3.60928E-12 | yes | up | 2.72 | 1.83 | 2.02 | 7.82 | 11.3 | 9.64 | 2.19 | 9.586666667 |
| gene19690 | VTN | vitronectin | 4.871 | 2.284357355 | 4.83655E-13 | 1.86315E-10 | yes | up | 20.57 | 12.28 | 20.9 | 94.23 | 78.54 | 74.07 | 17.91666667 | 82.28 |
| gene11397 | ATP2B2 | ATPase, Ca++ transporting, plasma membrane 2, transcript variant X2 | 4.865 | 2.282344174 | 5.48893E-07 | 5.28614E-05 | yes | up | 0.27 | 0.55 | 0.72 | 1.42 | 2.84 | 2.59 | 0.513333333 | 2.283333333 |
| gene12831 | LOC106041631 | chromosome unknown open reading frame, human C2orf40 | 4.834 | 2.273091585 | 3.81071E-09 | 6.00534E-07 | yes | up | 22.41 | 11.21 | 13.12 | 97.12 | 68.03 | 45.56 | 15.58 | 70.23666667 |
| gene7081 | CTHRC1 | collagen triple helix repeat containing 1 | 4.82 | 2.268997861 | 5.44002E-07 | 5.27568E-05 | yes | up | 5.69 | 12.34 | 3.43 | 32.45 | 39.11 | 23.42 | 7.153333333 | 31.66 |
| gene45 | PYROXD2 | pyridine nucleotide-disulphide oxidoreductase domain 2 | 4.77 | 2.254012777 | 0.000105858 | 0.004573311 | yes | up | 0.4 | 0.9 | 0.37 | 4.9 | 0.97 | 3.21 | 0.556666667 | 3.026666667 |
| gene6257 | C1QTNF2 | C1q and tumor necrosis factor related protein 2, transcript variant X1 | 4.758 | 2.250334105 | 5.52149E-13 | 2.01505E-10 | yes | up | 3.67 | 2.81 | 5.12 | 12.59 | 19.94 | 18.4 | 3.866666667 | 16.97666667 |
| gene5882 | ST6GALNAC5 | ST6 (alpha-N-acetyl-neuraminyl-2,3-beta-galactosyl-1,3)-N-acetylgalactosaminide alpha-2,6-sialyltransferase 5, transcript variant X1 | 4.755 | 2.249530021 | 0.001803505 | 0.043667759 | yes | up | 0.55 | 0.75 | 0.94 | 6.19 | 0.55 | 3.7 | 0.746666667 | 3.48 |
| gene19872 | LOC106049352 | soluble scavenger receptor cysteine-rich domain-containing protein SSC5D-like | 4.704 | 2.233976696 | 8.24791E-06 | 0.000534495 | yes | up | 2.22 | 0.84 | 0.7 | 3.95 | 7.84 | 4.7 | 1.253333333 | 5.496666667 |
| gene18926 | ADAMTSL1 | ADAMTS-like 1, transcript variant X1 | 4.701 | 2.232972153 | 6.69487E-14 | 3.09481E-11 | yes | up | 1.26 | 1.62 | 1.32 | 4.77 | 8.39 | 4.84 | 1.4 | 6 |
| gene2645 | COL21A1 | collagen, type XXI, alpha 1 | 4.697 | 2.231779732 | 1.83852E-08 | 2.4754E-06 | yes | up | 4.99 | 11.07 | 16.93 | 56.17 | 31.82 | 59.26 | 10.99666667 | 49.08333333 |
| gene18440 | DIO3 | deiodinase, iodothyronine, type III | 4.584 | 2.19672289 | 0.000528842 | 0.017175608 | yes | up | 1.9 | 4.67 | 0.52 | 11.98 | 7.65 | 10.15 | 2.363333333 | 9.926666667 |
| gene345 | TMEM26 | transmembrane protein 26, transcript variant X1 | 4.454 | 2.154958243 | 2.20884E-05 | 0.001255418 | yes | up | 0.47 | 0.68 | 0.81 | 1.71 | 1.98 | 4.92 | 0.653333333 | 2.87 |
| gene1447 | FAM180B | family with sequence similarity 180, member B | 4.428 | 2.146639802 | 4.60126E-12 | 1.32938E-09 | yes | up | 1.5 | 1.88 | 1.16 | 7.37 | 6.64 | 4.34 | 1.513333333 | 6.116666667 |
| gene13013 | ARPP21 | cAMP-regulated phosphoprotein, 21kDa, transcript variant X2 | 4.386 | 2.132965601 | 0.000359108 | 0.01235759 | yes | up | 0.16 | 0.09 | 0.18 | 0.49 | 0.58 | 0.55 | 0.143333333 | 0.54 |
| gene376 | LDB3 | LIM domain binding 3, transcript variant X1 | 4.345 | 2.119358945 | 9.61345E-06 | 0.000605997 | yes | up | 0.72 | 0.92 | 1.4 | 1.59 | 5.67 | 4.78 | 1.013333333 | 4.013333333 |
| gene12085 | COL20A1 | collagen, type XX, alpha 1, transcript variant X2 | 4.333 | 2.115414642 | 3.09583E-05 | 0.001696954 | yes | up | 0.35 | 0.34 | 0.33 | 0.64 | 2.28 | 1.1 | 0.34 | 1.34 |
| gene20331 | LOC106049842 | collagen alpha-1(I) chain-like | 4.324 | 2.112352353 | 3.27662E-12 | 1.00978E-09 | yes | up | 624.36 | 551.78 | 337.21 | 1564.19 | 2529.74 | 1670.44 | 504.45 | 1921.456667 |
| gene14577 | KCNAB1 | potassium channel, voltage gated subfamily A regulatory beta subunit 1 | 4.312 | 2.108495981 | 0.000542369 | 0.017492016 | yes | up | 1.02 | 0.82 | 2.41 | 1.73 | 8.23 | 6.78 | 1.416666667 | 5.58 |
| gene7134 | MRAP2 | melanocortin 2 receptor accessory protein 2, transcript variant X1 | 4.31 | 2.107598841 | 0.000449756 | 0.014961163 | yes | up | 0.24 | 0.62 | 0.18 | 0.73 | 1.88 | 0.91 | 0.346666667 | 1.173333333 |
| gene11471 | MFAP2 | microfibrillar-associated protein 2 | 4.251 | 2.087916533 | 8.93828E-17 | 6.524E-14 | yes | up | 8.74 | 9.5 | 12.87 | 38.21 | 46.25 | 38.85 | 10.37 | 41.10333333 |
| gene5015 | GPX7 | glutathione peroxidase 7, transcript variant X2 | 4.206 | 2.072459744 | 1.48983E-12 | 4.81738E-10 | yes | up | 4.03 | 2.44 | 3.51 | 9.7 | 13.11 | 15.47 | 3.326666667 | 12.76 |
| gene5049 | TRABD2B | TraB domain containing 2B | 4.195 | 2.068524908 | 1.46371E-17 | 1.19404E-14 | yes | up | 1.21 | 0.9 | 1.39 | 4.39 | 4.1 | 4.93 | 1.166666667 | 4.473333333 |
| gene13809 | VCAN | versican | 4.158 | 2.055908261 | 5.38752E-12 | 1.52478E-09 | yes | up | 23.06 | 19.09 | 22.47 | 55.11 | 73.23 | 112.92 | 21.54 | 80.42 |
| gene1574 | LOC106045879 | uncharacterized LOC106045879 | 4.151 | 2.053441069 | 4.14707E-05 | 0.002130057 | yes | up | 0.49 | 2.83 | 6.9 | 7.14 | 8.2 | 7.37 | 3.406666667 | 7.57 |
| gene13928 | LOC106042805 | semaphorin-3B-like | 4.142 | 2.050156068 | 2.06222E-06 | 0.00017125 | yes | up | 1.8 | 6.02 | 5.19 | 18.36 | 14.17 | 16.86 | 4.336666667 | 16.46333333 |
| gene954 | ABI3BP | ABI family, member 3 (NESH) binding protein, transcript variant X1 | 4.134 | 2.047580282 | 2.07658E-10 | 4.49969E-08 | yes | up | 25.04 | 24.12 | 44.77 | 125.29 | 87.82 | 147.3 | 31.31 | 120.1366667 |
| gene536 | LOC106034387 | uncharacterized LOC106034387, transcript variant X2 | 4.129 | 2.045835568 | 0.00204877 | 0.047432956 | yes | up | 0.41 | 0.07 | 0.27 | 0.63 | 1.38 | 0.86 | 0.25 | 0.956666667 |
| gene2324 | GPR1 | G protein-coupled receptor 1, transcript variant X2 | 4.109 | 2.038912606 | 1.10533E-06 | 9.64069E-05 | yes | up | 12.44 | 4.86 | 10.58 | 19.3 | 43.05 | 42.52 | 9.293333333 | 34.95666667 |
| gene4052 | ATRNL1 | attractin-like 1, transcript variant X1 | 4.107 | 2.038003753 | 1.36532E-15 | 8.33975E-13 | yes | up | 1.36 | 1.86 | 1.08 | 5.51 | 5.9 | 4.86 | 1.433333333 | 5.423333333 |
| gene18108 | LOC106047460 | sodium/nucleoside cotransporter 1-like | 4.085 | 2.030395768 | 0.001009605 | 0.028114864 | yes | up | 0.14 | 0.18 | 0.19 | 0.55 | 0.51 | 0.84 | 0.17 | 0.633333333 |
| gene13614 | PAPPA2 | pappalysin 2 | 4.024 | 2.008641956 | 4.04762E-07 | 4.15796E-05 | yes | up | 1.27 | 2.56 | 2.05 | 10.45 | 4.44 | 6.77 | 1.96 | 7.22 |
| gene1222 | KCNG1 | potassium channel, voltage gated modifier subfamily G, member 1, transcript variant X2 | 4.021 | 2.007580372 | 3.93446E-11 | 9.74341E-09 | yes | up | 1.59 | 0.94 | 1.2 | 3.78 | 4.89 | 4.9 | 1.243333333 | 4.523333333 |
| gene10435 | ADAM12 | ADAM metallopeptidase domain 12 | 4.017 | 2.006061446 | 2.94784E-23 | 5.11008E-20 | yes | up | 2.48 | 2.68 | 2.05 | 8.39 | 9.93 | 7.8 | 2.403333333 | 8.706666667 |
| gene4257 | CACNA1H | calcium channel, voltage-dependent, T type, alpha 1H subunit, transcript variant X1 | 4.006 | 2.002052245 | 9.30124E-11 | 2.18626E-08 | yes | up | 0.62 | 0.61 | 0.96 | 3.36 | 2.59 | 2.1 | 0.73 | 2.683333333 |
| gene13326 | ADGRB1 | adhesion G protein-coupled receptor B1 | 3.984 | 1.994129828 | 0.001025329 | 0.028320653 | yes | up | 0.2 | 0.08 | 0.08 | 0.66 | 0.29 | 0.36 | 0.12 | 0.436666667 |
| gene11103 | COL1A2 | collagen, type I, alpha 2 | 3.971 | 1.989495228 | 1.08817E-10 | 2.51513E-08 | yes | up | 667.13 | 791.73 | 370.82 | 1884.87 | 2697.75 | 2021.49 | 609.8933333 | 2201.37 |
| gene19388 | NEFM | neurofilament, medium polypeptide | 3.96 | 1.985350036 | 0.001114125 | 0.030001329 | yes | up | 2.49 | 0.31 | 0.94 | 4.43 | 5.51 | 3.53 | 1.246666667 | 4.49 |
| gene15519 | THBS2 | thrombospondin 2, transcript variant X1 | 3.93 | 1.974672582 | 9.44962E-12 | 2.56955E-09 | yes | up | 102.07 | 160.07 | 82.08 | 366.88 | 381.32 | 485.12 | 114.74 | 411.1066667 |
| gene15122 | LOC106044084 | collagen alpha-1(V) chain-like | 3.922 | 1.971509488 | 2.63867E-08 | 3.38824E-06 | yes | up | 3.88 | 5.67 | 7.92 | 14.22 | 26.99 | 21.55 | 5.823333333 | 20.92 |
| gene18276 | ACTG2 | actin, gamma 2, smooth muscle, enteric | 3.917 | 1.969698336 | 0.000209567 | 0.0081181 | yes | up | 19.93 | 20.09 | 21.93 | 24.28 | 145.56 | 52.96 | 20.65 | 74.26666667 |
| gene11690 | SGCG | sarcoglycan, gamma (35kDa dystrophin-associated glycoprotein), transcript variant X2 | 3.911 | 1.967459392 | 9.78374E-05 | 0.004293699 | yes | up | 2.11 | 1.11 | 5.31 | 10.37 | 7.73 | 12.37 | 2.843333333 | 10.15666667 |
| gene172 | SCD | stearoyl-CoA desaturase (delta-9-desaturase) | 3.891 | 1.960131816 | 1.26551E-08 | 1.73763E-06 | yes | up | 621.2 | 542.48 | 322.46 | 2524.2 | 1153.77 | 1676.35 | 495.38 | 1784.773333 |
| gene6322 | IQCA1 | IQ motif containing with AAA domain 1, transcript variant X1 | 3.879 | 1.955526356 | 6.88802E-08 | 8.45337E-06 | yes | up | 1.32 | 0.97 | 1.28 | 4.99 | 4.55 | 2.82 | 1.19 | 4.12 |
| gene15989 | LOC106045070 | uncharacterized LOC106045070, transcript variant X1 | 3.87 | 1.952242428 | 6.467E-07 | 6.05975E-05 | yes | up | 0.31 | 0.47 | 0.44 | 1 | 1.57 | 1.35 | 0.406666667 | 1.306666667 |
| gene9834 | COL15A1 | collagen, type XV, alpha 1 | 3.866 | 1.950664175 | 7.20137E-05 | 0.00339689 | yes | up | 2.68 | 2.77 | 3.88 | 6.28 | 21.17 | 5.7 | 3.11 | 11.05 |
| gene582 | SORCS2 | sortilin-related VPS10 domain containing receptor 2, transcript variant X2 | 3.814 | 1.931336311 | 4.7105E-19 | 4.66609E-16 | yes | up | 12.87 | 10.84 | 8.9 | 39.67 | 43.12 | 30.85 | 10.87 | 37.88 |
| gene15858 | CPAMD8 | C3 and PZP-like, alpha-2-macroglobulin domain containing 8 | 3.807 | 1.928689387 | 4.56455E-06 | 0.000336708 | yes | up | 3.16 | 5.05 | 1.8 | 6.7 | 14.83 | 13.44 | 3.336666667 | 11.65666667 |
| gene18606 | FKBP10 | FK506 binding protein 10, 65 kDa, transcript variant X2 | 3.803 | 1.927097778 | 3.44242E-12 | 1.03781E-09 | yes | up | 41.87 | 33.49 | 19.23 | 103.46 | 142.1 | 110.53 | 31.53 | 118.6966667 |
| gene18686 | MFAP5 | microfibrillar associated protein 5, transcript variant X2 | 3.795 | 1.924227217 | 1.75678E-14 | 9.02336E-12 | yes | up | 109.18 | 57.76 | 82.21 | 278.03 | 306.8 | 282.38 | 83.05 | 289.07 |
| gene11947 | LOC106040733 | collagen alpha-1(V) chain | 3.734 | 1.900819052 | 1.0607E-11 | 2.77544E-09 | yes | up | 10.98 | 12.12 | 15.16 | 31.87 | 60.68 | 41.66 | 12.75333333 | 44.73666667 |
| gene14669 | CORO2B | coronin, actin binding protein, 2B | 3.732 | 1.899847043 | 5.76144E-07 | 5.43535E-05 | yes | up | 2.6 | 1.66 | 2.08 | 5.45 | 10.65 | 5.59 | 2.113333333 | 7.23 |
| gene2867 | LOC106030608 | chromosome unknown open reading frame, human C8orf34, transcript variant X1 | 3.714 | 1.892944241 | 0.000163752 | 0.006544416 | yes | up | 0.71 | 2.01 | 0.71 | 4.99 | 3.77 | 2.88 | 1.143333333 | 3.88 |
| gene1416 | CREB3L1 | cAMP responsive element binding protein 3-like 1, transcript variant X1 | 3.712 | 1.892351523 | 3.73852E-14 | 1.85163E-11 | yes | up | 15.27 | 19.11 | 15.29 | 40.64 | 66.81 | 58.7 | 16.55666667 | 55.38333333 |
| gene16636 | PRELP | proline/arginine-rich end leucine-rich repeat protein | 3.708 | 1.890795646 | 3.77353E-25 | 7.47591E-22 | yes | up | 26.33 | 30.48 | 30.91 | 89.88 | 113.53 | 93.52 | 29.24 | 98.97666667 |
| gene1575 | LOC106045840 | histone H2A.J | 3.703 | 1.888668333 | 3.14448E-06 | 0.000246371 | yes | up | 2.26 | 1.95 | 3.63 | 8.95 | 11.34 | 6.8 | 2.613333333 | 9.03 |
| gene11408 | TIMP4 | TIMP metallopeptidase inhibitor 4, transcript variant X1 | 3.7 | 1.887527343 | 1.71868E-10 | 3.78327E-08 | yes | up | 20.49 | 23.29 | 27.84 | 116.93 | 73.06 | 68.09 | 23.87333333 | 86.02666667 |
| gene7958 | KCNE4 | potassium channel, voltage gated subfamily E regulatory beta subunit 4 | 3.687 | 1.882258923 | 5.34489E-08 | 6.67775E-06 | yes | up | 9.67 | 11 | 11.62 | 19.25 | 42.14 | 47.15 | 10.76333333 | 36.18 |
| gene12651 | RASL10B | RAS-like, family 10, member B | 3.646 | 1.866500063 | 5.30832E-10 | 1.02244E-07 | yes | up | 8.17 | 9.77 | 13.1 | 42.59 | 25.86 | 38.21 | 10.34666667 | 35.55333333 |
| gene15538 | AGT | angiotensinogen (serpin peptidase inhibitor, clade A, member 8), transcript variant X1 | 3.646 | 1.866411254 | 7.15974E-07 | 6.61942E-05 | yes | up | 6.59 | 3.28 | 2.76 | 14.66 | 10.27 | 17.02 | 4.21 | 13.98333333 |
| gene12646 | LOC106041493 | schlafen family member 13-like, transcript variant X4 | 3.603 | 1.849041299 | 0.000633717 | 0.019529738 | yes | up | 0.34 | 0.47 | 0.79 | 1.75 | 0.83 | 2.69 | 0.533333333 | 1.756666667 |
| gene10243 | LOC106038813 | synaptotagmin-5-like | 3.586 | 1.842325129 | 6.42605E-06 | 0.000435888 | yes | up | 2.5 | 0.97 | 1.23 | 6.47 | 4.24 | 4.99 | 1.566666667 | 5.233333333 |
| gene6420 | MSTN | myostatin | 3.566 | 1.834252503 | 0.000347555 | 0.012079935 | yes | up | 7.29 | 6.94 | 1.59 | 13.79 | 22.5 | 15.26 | 5.273333333 | 17.18333333 |
| gene1571 | LOC106045128 | uncharacterized LOC106045128 | 3.547 | 1.826537214 | 0.000866535 | 0.024983583 | yes | up | 1.12 | 3.87 | 2.81 | 16.97 | 3.96 | 22.2 | 2.6 | 14.37666667 |
| gene922 | DPT | dermatopontin | 3.535 | 1.821565047 | 7.97706E-15 | 4.25484E-12 | yes | up | 33.48 | 39.71 | 24.19 | 108.23 | 92.56 | 114.2 | 32.46 | 104.9966667 |
| gene15320 | PHACTR1 | phosphatase and actin regulator 1, transcript variant X2 | 3.529 | 1.819282 | 1.11074E-13 | 4.96894E-11 | yes | up | 1.29 | 1.78 | 1.52 | 4.84 | 5.7 | 4.21 | 1.53 | 4.916666667 |
| gene17418 | LOC106046723 | uncharacterized protein C16orf45-like, transcript variant X1 | 3.516 | 1.813891171 | 2.05682E-05 | 0.001188498 | yes | up | 0.4 | 0.47 | 0.44 | 1.83 | 1 | 1.28 | 0.436666667 | 1.37 |
| gene9984 | HAS2 | hyaluronan synthase 2 | 3.515 | 1.813516091 | 3.96761E-05 | 0.002076332 | yes | up | 6.07 | 3.74 | 7.28 | 9.12 | 16.24 | 29.92 | 5.696666667 | 18.42666667 |
| gene15276 | LOC106044301 | uncharacterized LOC106044301, transcript variant X1 | 3.476 | 1.797332258 | 7.79298E-05 | 0.003604408 | yes | up | 0.61 | 0.41 | 0.48 | 2.54 | 1.18 | 0.98 | 0.5 | 1.566666667 |
| gene9875 | TMEM108 | transmembrane protein 108, transcript variant X2 | 3.473 | 1.796275991 | 2.27402E-07 | 2.46376E-05 | yes | up | 0.44 | 0.56 | 0.29 | 1.63 | 1.15 | 1.63 | 0.43 | 1.47 |
| gene15380 | LOC106044418 | uncharacterized LOC106044418 | 3.463 | 1.792066452 | 3.47086E-10 | 6.97593E-08 | yes | up | 1.08 | 0.71 | 0.58 | 2.77 | 3.72 | 1.82 | 0.79 | 2.77 |
| gene19703 | OGN | osteoglycin | 3.446 | 1.784762622 | 3.42229E-05 | 0.001828351 | yes | up | 127.9 | 206.9 | 51.43 | 437.77 | 443.83 | 335.52 | 128.7433333 | 405.7066667 |
| gene1566 | LOC106045814 | histone H2B 7-like | 3.442 | 1.783191011 | 0.00083634 | 0.024469107 | yes | up | 3.33 | 9.33 | 5.79 | 12.14 | 12.34 | 34.1 | 6.15 | 19.52666667 |
| gene10429 | TEX36 | testis expressed 36 | 3.432 | 1.779132536 | 7.55098E-12 | 2.09434E-09 | yes | up | 5.89 | 3.62 | 4.86 | 17.82 | 16.84 | 15.54 | 4.79 | 16.73333333 |
| gene19871 | LOC106049351 | DNA-directed RNA polymerase II subunit RPB1-like | 3.428 | 1.777567032 | 0.000123024 | 0.005185706 | yes | up | 1.69 | 1.98 | 1.32 | 3 | 7.76 | 5.25 | 1.663333333 | 5.336666667 |
| gene13507 | SERPINH1 | serpin peptidase inhibitor, clade H (heat shock protein 47), member 1, (collagen binding protein 1) | 3.415 | 1.77187084 | 1.35161E-12 | 4.57173E-10 | yes | up | 111.07 | 59.03 | 73.21 | 198.02 | 339.77 | 253.55 | 81.10333333 | 263.78 |
| gene7965 | PAX3 | paired box 3 | 3.403 | 1.766661077 | 1.43283E-07 | 1.63896E-05 | yes | up | 1.86 | 1.66 | 3.09 | 8.25 | 5.67 | 6.71 | 2.203333333 | 6.876666667 |
| gene12600 | SERPINF1 | serpin peptidase inhibitor, clade F (alpha-2 antiplasmin, pigment epithelium derived factor), member 1 | 3.394 | 1.763083066 | 1.44183E-07 | 1.63896E-05 | yes | up | 196.87 | 115 | 166.1 | 345.36 | 734.39 | 406.64 | 159.3233333 | 495.4633333 |
| gene5886 | AK5 | adenylate kinase 5, transcript variant X1 | 3.39 | 1.761167625 | 0.000550369 | 0.017627056 | yes | up | 3.43 | 5.01 | 5.7 | 4.85 | 21.55 | 17.46 | 4.713333333 | 14.62 |
| gene14944 | LOC106043950 | 16 kDa beta-galactoside-binding lectin | 3.374 | 1.754621947 | 0.00141505 | 0.036273402 | yes | up | 6 | 8.19 | 7.69 | 4.77 | 26.54 | 15.5 | 7.293333333 | 15.60333333 |
| gene17976 | CERCAM | cerebral endothelial cell adhesion molecule | 3.373 | 1.754000512 | 1.68528E-05 | 0.001003066 | yes | up | 0.58 | 0.46 | 0.89 | 1.4 | 2.54 | 2.01 | 0.643333333 | 1.983333333 |
| gene15970 | LOC106045060 | netrin-4-like | 3.339 | 1.739630014 | 1.02441E-06 | 9.04871E-05 | yes | up | 8.6 | 15.69 | 6.41 | 35.74 | 34.81 | 23.25 | 10.23333333 | 31.26666667 |
| gene6002 | PDGFD | platelet derived growth factor D | 3.339 | 1.739475997 | 3.11935E-09 | 5.08932E-07 | yes | up | 40.29 | 23.69 | 37.38 | 73.35 | 107.68 | 126.52 | 33.78666667 | 102.5166667 |
| gene9774 | KIAA1644 | KIAA1644 ortholog, transcript variant X7 | 3.333 | 1.736656879 | 0.000261679 | 0.009703097 | yes | up | 3.17 | 4.25 | 4.63 | 14.23 | 3.72 | 16.71 | 4.016666667 | 11.55333333 |
| gene3658 | LOC106031513 | uncharacterized LOC106031513 | 3.325 | 1.733459326 | 0.000319413 | 0.011328964 | yes | up | 1.04 | 1.74 | 0.58 | 4.4 | 3.52 | 2.27 | 1.12 | 3.396666667 |
| gene20076 | IGFBP4 | insulin-like growth factor binding protein 4 | 3.321 | 1.73151037 | 2.71604E-10 | 5.53912E-08 | yes | up | 77.27 | 98.73 | 57.17 | 188.29 | 295.8 | 227.77 | 77.72333333 | 237.2866667 |
| gene19161 | IL11RA | interleukin 11 receptor, alpha, transcript variant X1 | 3.317 | 1.729761607 | 8.81563E-06 | 0.000565996 | yes | up | 16.22 | 11.27 | 26.72 | 36.44 | 72.5 | 56.35 | 18.07 | 55.09666667 |
| gene3647 | PKDCC | protein kinase domain containing, cytoplasmic | 3.316 | 1.729285754 | 3.98794E-06 | 0.000297337 | yes | up | 6.73 | 5.49 | 2.69 | 18.08 | 16.88 | 10.67 | 4.97 | 15.21 |
| gene13512 | DGAT2 | diacylglycerol O-acyltransferase 2 | 3.307 | 1.725594564 | 4.84365E-07 | 4.79798E-05 | yes | up | 101.06 | 83.01 | 87.15 | 435.67 | 215.76 | 171.11 | 90.40666667 | 274.18 |
| gene5426 | MYH11 | myosin, heavy chain 11, smooth muscle, transcript variant X1 | 3.296 | 1.720824987 | 0.001613998 | 0.040068926 | yes | up | 48.82 | 48.01 | 89.38 | 51.46 | 323.2 | 183.9 | 62.07 | 186.1866667 |
| gene15396 | GFRA2 | GDNF family receptor alpha 2 | 3.295 | 1.720268351 | 3.35306E-05 | 0.001802605 | yes | up | 12.8 | 6.17 | 18.12 | 34 | 27.09 | 51.93 | 12.36333333 | 37.67333333 |
| gene864 | LOC106037591 | galactosylgalactosylxylosylprotein 3-beta-glucuronosyltransferase 1-like | 3.291 | 1.718678363 | 7.00847E-07 | 6.52305E-05 | yes | up | 1.56 | 2.52 | 1.37 | 5.04 | 7.19 | 4.13 | 1.816666667 | 5.453333333 |
| gene16638 | FMOD | fibromodulin | 3.287 | 1.716858402 | 3.42783E-05 | 0.001828351 | yes | up | 0.72 | 1.06 | 0.36 | 2.86 | 3.29 | 2.19 | 0.713333333 | 2.78 |
| gene5196 | ENKD1 | enkurin domain containing 1, transcript variant X4 | 3.28 | 1.713640983 | 9.13274E-06 | 0.000580976 | yes | up | 3.05 | 5.71 | 3.11 | 14.71 | 14.21 | 7.48 | 3.956666667 | 12.13333333 |
| gene5200 | NDRG4 | NDRG family member 4, transcript variant X1 | 3.273 | 1.710464938 | 0.000267266 | 0.009823487 | yes | up | 15.55 | 7.87 | 4.01 | 17.01 | 26.64 | 35.11 | 9.143333333 | 26.25333333 |
| gene9100 | PON2 | paraoxonase 2 | 3.258 | 1.703767603 | 1.38314E-15 | 8.33975E-13 | yes | up | 35.3 | 23.65 | 24.49 | 79.6 | 73.94 | 95.38 | 27.81333333 | 82.97333333 |
| gene14670 | ITGA11 | integrin, alpha 11 | 3.256 | 1.703072399 | 8.14279E-05 | 0.003726873 | yes | up | 3.39 | 4.17 | 3.27 | 6.6 | 18.88 | 6.72 | 3.61 | 10.73333333 |
| gene2135 | SPEG | SPEG complex locus | 3.249 | 1.700103864 | 0.001855384 | 0.044516371 | yes | up | 8.71 | 6.54 | 14.28 | 7.92 | 46.64 | 33.61 | 9.843333333 | 29.39 |
| gene3875 | NPAS3 | neuronal PAS domain protein 3 | 3.248 | 1.699408485 | 1.43155E-06 | 0.000121053 | yes | up | 0.82 | 1.43 | 0.89 | 2.54 | 3.23 | 3.53 | 1.046666667 | 3.1 |
| gene18712 | LOC106048094 | uncharacterized LOC106048094, transcript variant X3 | 3.239 | 1.695734519 | 0.001748965 | 0.042552017 | yes | up | 0.27 | 0.25 | 0.23 | 1.07 | 0.69 | 0.48 | 0.25 | 0.746666667 |
| gene766 | ASB2 | ankyrin repeat and SOCS box containing 2 | 3.188 | 1.67266167 | 0.000128903 | 0.00540069 | yes | up | 2.61 | 1.23 | 1.6 | 2.52 | 6.55 | 6.5 | 1.813333333 | 5.19 |
| gene6661 | LOC106034905 | uncharacterized LOC106034905, transcript variant X2 | 3.186 | 1.671557862 | 5.01416E-16 | 3.31126E-13 | yes | up | 2.98 | 3.3 | 2.41 | 8.54 | 8.06 | 8.66 | 2.896666667 | 8.42 |
| gene10450 | LOC106039008 | uncharacterized LOC106039008, transcript variant X1 | 3.181 | 1.669632689 | 0.000361705 | 0.012402849 | yes | up | 2.35 | 0.96 | 0.93 | 4.49 | 6.01 | 2.16 | 1.413333333 | 4.22 |
| gene19023 | PI16 | peptidase inhibitor 16, transcript variant X1 | 3.179 | 1.668537144 | 0.000795407 | 0.023620361 | yes | up | 45.6 | 22.81 | 33.89 | 55.13 | 190.98 | 55.8 | 34.1 | 100.6366667 |
| gene5593 | LOC106033657 | tropomodulin-2, transcript variant X1 | 3.154 | 1.657200425 | 0.001412517 | 0.036273402 | yes | up | 1.24 | 1.08 | 1.11 | 2.38 | 1.78 | 5.69 | 1.143333333 | 3.283333333 |
| gene9532 | RSPO1 | R-spondin 1, transcript variant X2 | 3.146 | 1.65329452 | 0.000631111 | 0.019492756 | yes | up | 1.23 | 1.44 | 2.66 | 7.13 | 2.23 | 5.87 | 1.776666667 | 5.076666667 |
| gene7036 | MATN2 | matrilin 2, transcript variant X1 | 3.144 | 1.652381577 | 1.79914E-05 | 0.001057225 | yes | up | 7.69 | 7.46 | 4.27 | 13.08 | 31.89 | 13.67 | 6.473333333 | 19.54666667 |
| gene910 | CCDC80 | coiled-coil domain containing 80 | 3.107 | 1.635567052 | 3.00457E-07 | 3.20519E-05 | yes | up | 30.96 | 34.74 | 14.92 | 71.17 | 86.14 | 70.79 | 26.87333333 | 76.03333333 |
| gene13622 | ANGPTL1 | angiopoietin-like 1 | 3.093 | 1.628809408 | 6.4899E-09 | 9.51587E-07 | yes | up | 14.58 | 15.33 | 10.27 | 28.17 | 50.33 | 34.91 | 13.39333333 | 37.80333333 |
| gene16080 | LOC106045192 | uncharacterized LOC106045192, transcript variant X1 | 3.088 | 1.626455992 | 0.002114718 | 0.048155837 | yes | up | 1.95 | 2.17 | 3.58 | 2.28 | 11.42 | 8.17 | 2.566666667 | 7.29 |
| gene16957 | LOC106046179 | receptor-type tyrosine-protein phosphatase V-like | 3.069 | 1.617629457 | 4.61107E-14 | 2.20504E-11 | yes | up | 3.55 | 3.34 | 4.44 | 9.53 | 12.14 | 9.59 | 3.776666667 | 10.42 |
| gene5014 | LOC106033012 | uncharacterized LOC106033012, transcript variant X3 | 3.041 | 1.604460559 | 3.19847E-06 | 0.000249193 | yes | up | 6.83 | 10.03 | 6.27 | 5.16 | 9.68 | 21.91 | 7.71 | 12.25 |
| gene16053 | FABP3 | fatty acid binding protein 3, muscle and heart | 3.038 | 1.603205392 | 4.27084E-07 | 4.32321E-05 | yes | up | 42.57 | 21.48 | 26.4 | 103.12 | 82.22 | 71.32 | 30.15 | 85.55333333 |
| gene5748 | LOC106033870 | fatty acid desaturase 2-like | 3.017 | 1.59331745 | 8.63339E-08 | 1.02332E-05 | yes | up | 1.38 | 3.71 | 1.41 | 10.21 | 5.14 | 7.11 | 2.166666667 | 7.486666667 |
| gene6605 | SMIM5 | small integral membrane protein 5, transcript variant X5 | 2.971 | 1.571011335 | 3.15547E-17 | 2.43112E-14 | yes | up | 6.4 | 8.52 | 6.13 | 19.25 | 17.86 | 17.81 | 7.016666667 | 18.30666667 |
| gene5436 | XYLT1 | xylosyltransferase I, transcript variant X1 | 2.962 | 1.56663711 | 1.64182E-05 | 0.000985658 | yes | up | 6.34 | 6.08 | 10.8 | 12.59 | 21.49 | 28.36 | 7.74 | 20.81333333 |
| gene13457 | CAMK4 | calcium/calmodulin-dependent protein kinase IV, transcript variant X1 | 2.922 | 1.54712719 | 7.31123E-07 | 6.71471E-05 | yes | up | 1.02 | 1.72 | 1.66 | 3.28 | 3.69 | 4.81 | 1.466666667 | 3.926666667 |
| gene9618 | ADCY3 | adenylate cyclase 3, transcript variant X1 | 2.909 | 1.540497656 | 0.000516834 | 0.016864591 | yes | up | 2.01 | 3.45 | 2.72 | 3.38 | 10.87 | 7.48 | 2.726666667 | 7.243333333 |
| gene19689 | SARM1 | sterile alpha and TIR motif containing 1 | 2.875 | 1.523701461 | 0.00087675 | 0.025173433 | yes | up | 0.75 | 0.56 | 1.57 | 1.86 | 2.31 | 3.39 | 0.96 | 2.52 |
| gene19942 | LOC106049430 | IgGFc-binding protein-like | 2.862 | 1.516791342 | 3.45981E-06 | 0.000268048 | yes | up | 22.39 | 12.75 | 21.31 | 32.65 | 49.05 | 65.08 | 18.81666667 | 48.92666667 |
| gene6421 | LOC106034495 | chromosome unknown open reading frame, human C2orf88, transcript variant X2 | 2.86 | 1.516258596 | 3.95003E-06 | 0.000296103 | yes | up | 1.88 | 2.75 | 1.9 | 4.43 | 6.84 | 4.63 | 2.176666667 | 5.3 |
| gene16689 | LOC106045839 | receptor-type tyrosine-protein phosphatase V-like | 2.85 | 1.511144327 | 7.96034E-07 | 7.2153E-05 | yes | up | 3.48 | 3.25 | 5.08 | 10.13 | 12.76 | 8.01 | 3.936666667 | 10.3 |
| gene4729 | LOC106032884 | uncharacterized LOC106032884, transcript variant X3 | 2.841 | 1.506316532 | 0.000807762 | 0.023884956 | yes | up | 0.1 | 0.13 | 0.21 | 0.34 | 0.52 | 0.3 | 0.146666667 | 0.386666667 |
| gene10082 | GRIA3 | glutamate receptor, ionotropic, AMPA 3, transcript variant X2 | 2.832 | 1.501818822 | 1.00068E-07 | 1.17605E-05 | yes | up | 9.23 | 14.57 | 4.77 | 7.01 | 5.46 | 7.47 | 9.523333333 | 6.646666667 |
| gene13859 | STC2 | stanniocalcin 2 | 2.832 | 1.50206416 | 0.000168138 | 0.006681203 | yes | up | 6.8 | 6.13 | 2.78 | 15.26 | 17.2 | 8.16 | 5.236666667 | 13.54 |
| gene14586 | GPR149 | G protein-coupled receptor 149 | 2.826 | 1.498637728 | 4.1098E-05 | 0.002118764 | yes | up | 0.43 | 0.56 | 0.4 | 1.42 | 1.34 | 0.8 | 0.463333333 | 1.186666667 |
| gene20205 | LOXL2 | lysyl oxidase-like 2, transcript variant X5 | 2.822 | 1.496463196 | 1.56029E-07 | 1.75919E-05 | yes | up | 11.11 | 14.54 | 21.62 | 35.64 | 44.65 | 40.42 | 15.75666667 | 40.23666667 |
| gene6971 | TLR3 | toll-like receptor 3, transcript variant X3 | 2.81 | 1.49052391 | 7.10918E-08 | 8.64826E-06 | yes | up | 1.88 | 2.12 | 1.98 | 4.04 | 4.56 | 6.66 | 1.993333333 | 5.086666667 |
| gene13393 | FBLN7 | fibulin 7 | 2.806 | 1.488263681 | 2.19614E-06 | 0.000181287 | yes | up | 1.03 | 1.37 | 1.46 | 3.02 | 2.62 | 4.01 | 1.286666667 | 3.216666667 |
| gene17125 | GLDC | glycine dehydrogenase (decarboxylating) | 2.803 | 1.486936914 | 0.000205803 | 0.007994629 | yes | up | 0.77 | 1.9 | 0.98 | 3.77 | 3.71 | 3.07 | 1.216666667 | 3.516666667 |
| gene18711 | LOC106048107 | uncharacterized LOC106048107, transcript variant X3 | 2.782 | 1.475897498 | 5.77294E-06 | 0.000404339 | yes | up | 0.55 | 0.51 | 0.35 | 0.88 | 1.49 | 1.25 | 0.47 | 1.206666667 |
| gene12723 | ALX1 | ALX homeobox 1 | 2.764 | 1.466617214 | 0.001075516 | 0.029360727 | yes | up | 0.94 | 2.1 | 1.66 | 2.77 | 4.74 | 4.39 | 1.566666667 | 3.966666667 |
| gene19104 | UNC13B | unc-13 homolog B (C. elegans) | 2.751 | 1.459733779 | 0.001646105 | 0.040619552 | yes | up | 0.4 | 0.45 | 0.28 | 1.46 | 0.54 | 0.81 | 0.376666667 | 0.936666667 |
| gene7467 | NIPA1 | non imprinted in Prader-Willi/Angelman syndrome 1 | 2.745 | 1.456895361 | 2.41274E-06 | 0.000195672 | yes | up | 17.03 | 15.92 | 21.32 | 49.61 | 28.26 | 58.54 | 18.09 | 45.47 |
| gene11433 | FBLN2 | fibulin 2, transcript variant X4 | 2.741 | 1.45494013 | 5.08005E-13 | 1.90406E-10 | yes | up | 34.31 | 44.16 | 37.02 | 84.42 | 112.87 | 91.24 | 38.49666667 | 96.17666667 |
| gene3537 | LOC106031329 | melanotransferrin-like, transcript variant X3 | 2.74 | 1.454360104 | 0.000245371 | 0.009196774 | yes | up | 2.32 | 2.1 | 2.75 | 5.25 | 9 | 3.27 | 2.39 | 5.84 |
| gene2864 | SULF1 | sulfatase 1 | 2.727 | 1.447220031 | 2.4321E-13 | 9.9201E-11 | yes | up | 21.85 | 20.84 | 27.38 | 58.68 | 67.5 | 51.39 | 23.35666667 | 59.19 |
| gene18969 | ADAMTS10 | ADAM metallopeptidase with thrombospondin type 1 motif, 10, transcript variant X2 | 2.72 | 1.443624969 | 4.42164E-07 | 4.42223E-05 | yes | up | 2.87 | 3.26 | 2.31 | 6.03 | 10.05 | 5.55 | 2.813333333 | 7.21 |
| gene6683 | F13A1 | coagulation factor XIII, A1 polypeptide, transcript variant X2 | 2.707 | 1.436756503 | 3.33392E-05 | 0.001802605 | yes | up | 33.48 | 52.83 | 35.14 | 60.61 | 143.52 | 98.05 | 40.48333333 | 100.7266667 |
| gene18065 | LOC106047414 | uncharacterized LOC106047414, transcript variant X5 | 2.704 | 1.434910056 | 0.000326488 | 0.011549442 | yes | up | 3.02 | 0.44 | 2.97 | 3.72 | 6.95 | 6.21 | 2.143333333 | 5.626666667 |
| gene4169 | LOC106032214 | uncharacterized LOC106032214 | 2.704 | 1.435179553 | 0.002090241 | 0.047913163 | yes | up | 4.93 | 5.55 | 5.26 | 8.65 | 17.2 | 14.76 | 5.246666667 | 13.53666667 |
| gene13598 | SERPINC1 | serpin peptidase inhibitor, clade C (antithrombin), member 1 | 2.694 | 1.429961416 | 0.000985629 | 0.027669441 | yes | up | 3.44 | 1.43 | 1.17 | 6.77 | 4.71 | 3.3 | 2.013333333 | 4.926666667 |
| gene19706 | ECM2 | extracellular matrix protein 2, female organ and adipocyte specific | 2.682 | 1.423135813 | 1.64998E-09 | 2.93358E-07 | yes | up | 50.95 | 52.25 | 34.95 | 112.23 | 90.5 | 139.08 | 46.05 | 113.9366667 |
| gene15251 | FAM132A | family with sequence similarity 132, member A, transcript variant X2 | 2.679 | 1.421560734 | 0.000604203 | 0.01891443 | yes | up | 4.63 | 3.3 | 2.7 | 14.23 | 6.53 | 5.18 | 3.543333333 | 8.646666667 |
| gene14684 | LOC106043657 | uncharacterized LOC106043657 | 2.677 | 1.420680623 | 0.000538961 | 0.017422632 | yes | up | 0.34 | 0.53 | 0.44 | 1.24 | 0.94 | 1.01 | 0.436666667 | 1.063333333 |
| gene758 | LOC106036370 | serine protease inhibitor 2.1-like, transcript variant X1 | 2.675 | 1.419487171 | 4.81599E-05 | 0.002428661 | yes | up | 11.71 | 1.83 | 5.55 | 4.5 | 2.7 | 3.8 | 6.363333333 | 3.666666667 |
| gene8976 | ZIC3 | Zic family member 3 | 2.675 | 1.41933826 | 0.001502249 | 0.037947509 | yes | up | 2.1 | 1.91 | 1.48 | 4.22 | 5.23 | 4.12 | 1.83 | 4.523333333 |
| gene11422 | EFCC1 | EF-hand and coiled-coil domain containing 1, transcript variant X1 | 2.665 | 1.413926224 | 1.77211E-05 | 0.001045769 | yes | up | 1.64 | 0.82 | 1 | 2.66 | 3.16 | 2.54 | 1.153333333 | 2.786666667 |
| gene1475 | LRRC4C | leucine rich repeat containing 4C | 2.663 | 1.413267292 | 0.002156817 | 0.048953738 | yes | up | 0.73 | 0.74 | 0.42 | 2.12 | 1.1 | 1.62 | 0.63 | 1.613333333 |
| gene14118 | LPAR1 | lysophosphatidic acid receptor 1, transcript variant X1 | 2.662 | 1.412504807 | 7.78967E-10 | 1.46592E-07 | yes | up | 3.41 | 4.89 | 4.84 | 8.84 | 13.58 | 15.47 | 4.38 | 12.63 |
| gene12088 | NKAIN4 | Na+/K+ transporting ATPase interacting 4, transcript variant X1 | 2.654 | 1.407938799 | 0.000498053 | 0.016367293 | yes | up | 9.56 | 5.83 | 7.55 | 12.47 | 11.79 | 27.15 | 7.646666667 | 17.13666667 |
| gene10594 | LOC106039209 | chromosome unknown open reading frame, human C3orf70, transcript variant X3 | 2.652 | 1.407104813 | 0.000192617 | 0.007567188 | yes | up | 3.97 | 1.94 | 2.23 | 4.14 | 7.84 | 7.76 | 2.713333333 | 6.58 |
| gene16701 | IGFBP5 | insulin-like growth factor binding protein 5 | 2.644 | 1.402590495 | 1.58582E-06 | 0.000132483 | yes | up | 86.57 | 41.08 | 78.35 | 151.74 | 193.98 | 170.73 | 68.66666667 | 172.15 |
| gene3718 | TGFB2 | transforming growth factor, beta 2 | 2.64 | 1.400507851 | 2.46654E-11 | 6.21928E-09 | yes | up | 6.53 | 5.37 | 5.07 | 12.01 | 13.9 | 15.6 | 5.656666667 | 13.83666667 |
| gene4127 | LOC106032022 | calcium-activated potassium channel subunit beta-2, transcript variant X2 | 2.639 | 1.400129444 | 0.001505419 | 0.037958453 | yes | up | 11.06 | 2.68 | 9.61 | 22.89 | 12.33 | 30.12 | 7.783333333 | 21.78 |
| gene4872 | SPARC | secreted protein, acidic, cysteine-rich (osteonectin) | 2.636 | 1.398390384 | 0.001344092 | 0.034906127 | yes | up | 2547.59 | 1140.49 | 772.95 | 3180.09 | 4961.98 | 2768.86 | 1487.01 | 3636.976667 |
| gene579 | ABLIM2 | actin binding LIM protein family, member 2, transcript variant X1 | 2.634 | 1.397485519 | 0.000567034 | 0.017994571 | yes | up | 1.83 | 0.74 | 0.91 | 2.83 | 3.25 | 2.18 | 1.16 | 2.753333333 |
| gene18083 | AP3B2 | adaptor-related protein complex 3, beta 2 subunit, transcript variant X1 | 2.631 | 1.395605436 | 8.59254E-09 | 1.21593E-06 | yes | up | 1.31 | 1.89 | 1.85 | 4.17 | 3.97 | 4 | 1.683333333 | 4.046666667 |
| gene7533 | GLT8D2 | glycosyltransferase 8 domain containing 2, transcript variant X1 | 2.624 | 1.39163461 | 1.49371E-12 | 4.81738E-10 | yes | up | 9.05 | 10.3 | 8.56 | 17.92 | 25.91 | 20.53 | 9.303333333 | 21.45333333 |
| gene3243 | SEZ6L | seizure related 6 homolog (mouse)-like | 2.618 | 1.388601476 | 7.82219E-10 | 1.46592E-07 | yes | up | 2.83 | 2.35 | 2.96 | 7 | 6.18 | 5.93 | 2.713333333 | 6.37 |
| gene1418 | MDK | midkine (neurite growth-promoting factor 2) | 2.609 | 1.383460089 | 0.000990047 | 0.027737325 | yes | up | 3.18 | 9.22 | 8.23 | 22.77 | 16.5 | 16.21 | 6.876666667 | 18.49333333 |
| gene3233 | DRAM1 | DNA-damage regulated autophagy modulator 1, transcript variant X1 | 2.6 | 1.378493216 | 0.000216506 | 0.008363526 | yes | up | 9.62 | 16.58 | 11.77 | 44.44 | 17.52 | 28.53 | 12.65666667 | 30.16333333 |
| gene5570 | FBN1 | fibrillin 1, transcript variant X2 | 2.6 | 1.378424316 | 1.83333E-07 | 2.05037E-05 | yes | up | 61.43 | 76.32 | 69.79 | 124.47 | 223.58 | 143.61 | 69.18 | 163.8866667 |
| gene9740 | SSPN | sarcospan | 2.582 | 1.368553649 | 3.06512E-07 | 3.24481E-05 | yes | up | 4.57 | 6.63 | 4.96 | 10.48 | 12.31 | 15.32 | 5.386666667 | 12.70333333 |
| gene19299 | LOC106048738 | plectin-like | 2.555 | 1.353434622 | 0.001094176 | 0.029636781 | yes | up | 8.2 | 1.07 | 1.65 | 5.5 | 9.04 | 7.37 | 3.64 | 7.303333333 |
| gene11341 | TMEFF2 | transmembrane protein with EGF-like and two follistatin-like domains 2 | 2.554 | 1.35281979 | 0.000308796 | 0.011008694 | yes | up | 2.23 | 1.65 | 3.42 | 3.89 | 6.81 | 6.81 | 2.433333333 | 5.836666667 |
| gene19630 | NPR1 | natriuretic peptide receptor 1 | 2.552 | 1.351826042 | 4.15294E-09 | 6.32889E-07 | yes | up | 35.13 | 47.33 | 33.4 | 76.05 | 89.6 | 104.91 | 38.62 | 90.18666667 |
| gene124 | ACTA2 | actin, alpha 2, smooth muscle, aorta, transcript variant X2 | 2.551 | 1.350954973 | 0.002043542 | 0.047404387 | yes | up | 215.57 | 205.8 | 313.62 | 217.94 | 719.04 | 772.52 | 244.9966667 | 569.8333333 |
| gene14141 | B3GNT9 | UDP-GlcNAc:betaGal beta-1,3-N-acetylglucosaminyltransferase 9, transcript variant X1 | 2.544 | 1.347207926 | 6.75754E-06 | 0.000452723 | yes | up | 11.08 | 10.89 | 11.65 | 16.22 | 33.89 | 30.67 | 11.20666667 | 26.92666667 |
| gene20140 | LOC106049637 | leucine-rich repeat and fibronectin type III domain-containing protein 1-like protein | 2.542 | 1.345895311 | 7.06317E-05 | 0.003343075 | yes | up | 5.01 | 2.67 | 3.32 | 6.26 | 10.81 | 9.37 | 3.666666667 | 8.813333333 |
| gene20279 | LOC106049785 | collagen alpha-1(XI) chain-like | 2.527 | 1.337469821 | 0.000775366 | 0.023224133 | yes | up | 12.02 | 8.27 | 19.97 | 33.62 | 35.27 | 28.17 | 13.42 | 32.35333333 |
| gene2078 | SIX2 | SIX homeobox 2 | 2.526 | 1.33680602 | 6.36913E-09 | 9.49754E-07 | yes | up | 31.7 | 52.19 | 36.46 | 91.58 | 92.15 | 91.97 | 40.11666667 | 91.9 |
| gene14279 | MAB21L1 | mab-21-like 1 (C. elegans) | 2.508 | 1.326603018 | 1.9893E-05 | 0.001159146 | yes | up | 2.53 | 4.21 | 2.25 | 6.5 | 7.96 | 6.18 | 2.996666667 | 6.88 |
| gene19363 | ACTA1 | actin, alpha 1, skeletal muscle | 2.506 | 1.325630911 | 0.000451935 | 0.014993874 | yes | up | 72.12 | 53.43 | 81.75 | 99.32 | 258.28 | 120.06 | 69.1 | 159.22 |
| gene10104 | SYNPO2 | synaptopodin 2 | 2.503 | 1.323666337 | 0.00109263 | 0.029636781 | yes | up | 3.91 | 3.35 | 5.7 | 5.59 | 15.98 | 10.52 | 4.32 | 10.69666667 |
| gene6409 | COL5A2 | collagen, type V, alpha 2 | 2.493 | 1.317677854 | 2.95043E-12 | 9.29923E-10 | yes | up | 51.36 | 57.26 | 62.99 | 113.57 | 127.57 | 148.73 | 57.20333333 | 129.9566667 |
| gene7233 | PRICKLE2 | prickle homolog 2, transcript variant X1 | 2.491 | 1.316736211 | 0.000224729 | 0.008633092 | yes | up | 10.6 | 9.68 | 7.64 | 14.42 | 15.79 | 32.55 | 9.306666667 | 20.92 |
| gene16557 | FSTL1 | follistatin-like 1 | 2.487 | 1.314503582 | 2.59304E-08 | 3.38824E-06 | yes | up | 147.33 | 126.47 | 133.97 | 221.16 | 368.89 | 334.49 | 135.9233333 | 308.18 |
| gene6684 | NRN1 | neuritin 1 | 2.461 | 1.299115855 | 1.38289E-10 | 3.09321E-08 | yes | up | 6.2 | 6.03 | 6.95 | 13.56 | 15.21 | 14.58 | 6.393333333 | 14.45 |
| gene15930 | TERT | telomerase reverse transcriptase | 2.458 | 1.29769614 | 0.000764758 | 0.023005774 | yes | up | 4.56 | 4.9 | 2.56 | 12.02 | 12.92 | 6.09 | 4.006666667 | 10.34333333 |
| gene10754 | LYPD1 | LY6/PLAUR domain containing 1 | 2.457 | 1.297157326 | 6.84092E-05 | 0.003248966 | yes | up | 1.01 | 0.95 | 0.76 | 1.91 | 1.73 | 2.43 | 0.906666667 | 2.023333333 |
| gene5954 | LOC106034075 | uncharacterized LOC106034075, transcript variant X1 | 2.446 | 1.29048052 | 0.002044118 | 0.047404387 | yes | up | 0.42 | 0.41 | 0.51 | 0.79 | 1.4 | 0.81 | 0.446666667 | 1 |
| gene11987 | GSN | gelsolin, transcript variant X1 | 2.444 | 1.289206287 | 3.03179E-09 | 5.00534E-07 | yes | up | 3771.33 | 4301.25 | 3715.75 | 8667.84 | 6845.27 | 10615.71 | 3929.443333 | 8709.606667 |
| gene6982 | SLC25A4 | solute carrier family 25 (mitochondrial carrier; adenine nucleotide translocator), member 4 | 2.439 | 1.286285603 | 5.38464E-06 | 0.000379057 | yes | up | 4.04 | 3.2 | 3.21 | 6.4 | 9.54 | 7.41 | 3.483333333 | 7.783333333 |
| gene756 | GSC | goosecoid homeobox | 2.428 | 1.279630093 | 0.000297729 | 0.010724435 | yes | up | 10.92 | 6.94 | 12.52 | 25.02 | 15.46 | 25.31 | 10.12666667 | 21.93 |
| gene9790 | FBLN1 | fibulin 1, transcript variant X1 | 2.411 | 1.269578863 | 6.4434E-06 | 0.000435888 | yes | up | 18.65 | 25.31 | 26.34 | 43.47 | 68.98 | 41.64 | 23.43333333 | 51.36333333 |
| gene10241 | CHKA | choline kinase alpha, transcript variant X1 | 2.409 | 1.268555811 | 7.60024E-06 | 0.000499527 | yes | up | 37.97 | 27.81 | 39.59 | 53.42 | 66.36 | 84.15 | 35.12333333 | 67.97666667 |
| gene5881 | ST6GALNAC3 | ST6 (alpha-N-acetyl-neuraminyl-2,3-beta-galactosyl-1,3)-N-acetylgalactosaminide alpha-2,6-sialyltransferase 3, transcript variant X1 | 2.395 | 1.260107726 | 0.00013589 | 0.005625432 | yes | up | 0.98 | 0.87 | 1.25 | 2.81 | 1.68 | 1.55 | 1.033333333 | 2.013333333 |
| gene14089 | GRIN3A | glutamate receptor, ionotropic, N-methyl-D-aspartate 3A | 2.383 | 1.2525695 | 0.001330744 | 0.034624312 | yes | up | 2.85 | 2.03 | 1.76 | 7.57 | 4.04 | 2.82 | 2.213333333 | 4.81 |
| gene8538 | LUM | lumican | 2.382 | 1.25227019 | 1.44848E-05 | 0.000881033 | yes | up | 367 | 570.74 | 266.77 | 865.94 | 865.92 | 893.49 | 401.5033333 | 875.1166667 |
| gene16086 | ENHO | energy homeostasis associated | 2.371 | 1.245192431 | 0.001107579 | 0.029883087 | yes | up | 15.66 | 15.92 | 13.9 | 55 | 23.29 | 20.77 | 15.16 | 33.02 |
| gene12319 | WBSCR17 | Williams-Beuren syndrome chromosome region 17, transcript variant X1 | 2.369 | 1.243975827 | 5.82584E-06 | 0.000405994 | yes | up | 7.37 | 8.79 | 6.94 | 11.92 | 21.15 | 16.89 | 7.7 | 16.65333333 |
| gene13715 | IGF2 | insulin-like growth factor 2 | 2.365 | 1.241737367 | 2.16067E-10 | 4.54002E-08 | yes | up | 33.73 | 40.68 | 32.43 | 82.46 | 84.36 | 65.94 | 35.61333333 | 77.58666667 |
| gene16124 | HSPA12A | heat shock 70kDa protein 12A, transcript variant X1 | 2.353 | 1.234762713 | 0.000902518 | 0.025737127 | yes | up | 2.85 | 1.99 | 2.41 | 4.45 | 7.97 | 3.1 | 2.416666667 | 5.173333333 |
| gene5563 | FGF7 | fibroblast growth factor 7, transcript variant X1 | 2.348 | 1.231492544 | 2.16504E-09 | 3.7531E-07 | yes | up | 15.1 | 13.98 | 14.05 | 28.4 | 35.63 | 27.15 | 14.37666667 | 30.39333333 |
| gene10990 | NETO2 | neuropilin (NRP) and tolloid (TLL)-like 2 | 2.346 | 1.230023386 | 7.4828E-05 | 0.003482262 | yes | up | 2.14 | 1.79 | 1.7 | 4.12 | 3.83 | 4.17 | 1.876666667 | 4.04 |
| gene2902 | COLEC11 | collectin sub-family member 11, transcript variant X1 | 2.339 | 1.226180667 | 0.000545867 | 0.017551184 | yes | up | 4.14 | 2.78 | 3.77 | 4.58 | 10.53 | 7.43 | 3.563333333 | 7.513333333 |
| gene14890 | GALNT5 | polypeptide N-acetylgalactosaminyltransferase 5 | 2.307 | 1.205799623 | 2.80458E-06 | 0.000226127 | yes | up | 6.35 | 5.97 | 8.39 | 12.43 | 16.04 | 15.44 | 6.903333333 | 14.63666667 |
| gene10289 | LTBP2 | latent transforming growth factor beta binding protein 2, transcript variant X1 | 2.306 | 1.205473392 | 0.001908397 | 0.045473625 | yes | up | 11.66 | 30.77 | 16.68 | 56.08 | 30.86 | 38.04 | 19.70333333 | 41.66 |
| gene5653 | THSD4 | thrombospondin, type I, domain containing 4, transcript variant X1 | 2.303 | 1.20374153 | 2.0265E-05 | 0.001175881 | yes | up | 6.23 | 8.34 | 6.7 | 9.83 | 18.44 | 16.95 | 7.09 | 15.07333333 |
| gene18389 | SNTA1 | syntrophin, alpha 1 | 2.302 | 1.203043485 | 4.66387E-10 | 9.10965E-08 | yes | up | 5.45 | 3.36 | 4.35 | 8.7 | 8.03 | 10.3 | 4.386666667 | 9.01 |
| gene10050 | CHRDL1 | chordin-like 1, transcript variant X1 | 2.3 | 1.201323591 | 8.99667E-05 | 0.003973435 | yes | up | 11.02 | 11.08 | 7.66 | 13.61 | 27.54 | 21.25 | 9.92 | 20.8 |
| gene4107 | TNFSF10 | tumor necrosis factor (ligand) superfamily, member 10, transcript variant X1 | 2.291 | 1.196250446 | 5.09205E-05 | 0.002495284 | yes | up | 25.38 | 15.91 | 22.31 | 47.89 | 55.81 | 29.35 | 21.2 | 44.35 |
| gene11553 | MTHFR | methylenetetrahydrofolate reductase (NAD(P)H), transcript variant X1 | 2.285 | 1.192205761 | 0.001808423 | 0.043667759 | yes | up | 31.33 | 32.56 | 24.73 | 99.48 | 50.68 | 33.28 | 29.54 | 61.14666667 |
| gene4853 | ARSI | arylsulfatase family, member I | 2.284 | 1.191801068 | 0.000560421 | 0.017907645 | yes | up | 4.81 | 3.97 | 4.12 | 5.71 | 7.86 | 13.25 | 4.3 | 8.94 |
| gene2646 | BMP5 | bone morphogenetic protein 5, transcript variant X1 | 2.283 | 1.191198291 | 7.1308E-20 | 8.24083E-17 | yes | up | 45.96 | 45.29 | 47.18 | 92.62 | 100.94 | 92.2 | 46.14333333 | 95.25333333 |
| gene12311 | ELN | elastin | 2.28 | 1.188867086 | 0.00145959 | 0.037140538 | yes | up | 45.35 | 59.21 | 66.35 | 134.56 | 58.63 | 162.35 | 56.97 | 118.5133333 |
| gene9345 | CDKL5 | cyclin-dependent kinase-like 5, transcript variant X3 | 2.272 | 1.184254896 | 2.74263E-05 | 0.001533659 | yes | up | 2 | 1.81 | 1.53 | 2.68 | 4.87 | 3.37 | 1.78 | 3.64 |
| gene3132 | WNT5B | wingless-type MMTV integration site family, member 5B | 2.271 | 1.183633605 | 0.000222436 | 0.008568733 | yes | up | 32.14 | 29.09 | 17.6 | 68.95 | 58.41 | 36.79 | 26.27666667 | 54.71666667 |
| gene1185 | SLC2A10 | solute carrier family 2 (facilitated glucose transporter), member 10, transcript variant X1 | 2.27 | 1.182916132 | 1.74759E-05 | 0.001035707 | yes | up | 2.76 | 2.1 | 2.29 | 3.76 | 5.59 | 5.45 | 2.383333333 | 4.933333333 |
| gene8539 | DCN | decorin | 2.267 | 1.181092982 | 5.30097E-06 | 0.000378937 | yes | up | 1063.72 | 1481.06 | 815.47 | 2260.74 | 2186.55 | 2661.9 | 1120.083333 | 2369.73 |
| gene18061 | LOX | lysyl oxidase, transcript variant X1 | 2.266 | 1.180305104 | 7.23318E-05 | 0.00340033 | yes | up | 138.56 | 100.59 | 62.17 | 232.51 | 180.22 | 208.63 | 100.44 | 207.12 |
| gene12491 | OLFML3 | olfactomedin-like 3 | 2.26 | 1.176459985 | 4.51744E-12 | 1.32938E-09 | yes | up | 28.42 | 28.58 | 30.82 | 54.12 | 58.85 | 68.28 | 29.27333333 | 60.41666667 |
| gene1982 | LTBP1 | latent transforming growth factor beta binding protein 1, transcript variant X1 | 2.258 | 1.17505913 | 2.23214E-05 | 0.001263481 | yes | up | 11.83 | 19.97 | 21.51 | 32.96 | 37.22 | 40.66 | 17.77 | 36.94666667 |
| gene17618 | FZD2 | frizzled class receptor 2 | 2.252 | 1.171093666 | 1.17806E-05 | 0.000722892 | yes | up | 6.98 | 6.22 | 6.51 | 10.72 | 17.41 | 12.56 | 6.57 | 13.56333333 |
| gene5032 | CDKN2C | cyclin-dependent kinase inhibitor 2C (p18, inhibits CDK4) | 2.252 | 1.170951867 | 2.09783E-08 | 2.79738E-06 | yes | up | 29.46 | 34.12 | 22.39 | 63.05 | 66.46 | 52.94 | 28.65666667 | 60.81666667 |
| gene4826 | GFPT2 | glutamine-fructose-6-phosphate transaminase 2, transcript variant X1 | 2.246 | 1.167474476 | 1.08142E-06 | 9.49182E-05 | yes | up | 42.83 | 31.19 | 29.58 | 62.5 | 89.43 | 59 | 34.53333333 | 70.31 |
| gene8764 | LOC106037110 | dual specificity testis-specific protein kinase 2-like, transcript variant X1 | 2.245 | 1.167019743 | 0.000756482 | 0.022806281 | yes | up | 3.96 | 2.25 | 4.45 | 6.97 | 9.42 | 5.33 | 3.553333333 | 7.24 |
| gene4502 | LOC106032484 | protein SOGA3, transcript variant X4 | 2.241 | 1.164451278 | 4.01435E-07 | 4.15455E-05 | yes | up | 2.7 | 1.95 | 2.82 | 5.07 | 5.04 | 4.1 | 2.49 | 4.736666667 |
| gene20447 | LOC106049951 | acyl-CoA synthetase short-chain family member 3, mitochondrial-like, transcript variant X1 | 2.237 | 1.161883002 | 0.000875337 | 0.025173433 | yes | up | 3.61 | 4.03 | 2.38 | 6.04 | 9.65 | 4.97 | 3.34 | 6.886666667 |
| gene3122 | CECR1 | cat eye syndrome chromosome region, candidate 1, transcript variant X1 | 2.234 | 1.159449094 | 3.89215E-05 | 0.002044556 | yes | up | 2.76 | 4.36 | 4.93 | 8.68 | 9.02 | 6.91 | 4.016666667 | 8.203333333 |
| gene13526 | TSKU | tsukushi, small leucine rich proteoglycan | 2.215 | 1.147000074 | 0.000140726 | 0.005756885 | yes | up | 25.35 | 17.57 | 14.04 | 29.74 | 37.15 | 50.29 | 18.98666667 | 39.06 |
| gene20023 | SCARA5 | scavenger receptor class A, member 5 | 2.209 | 1.143138596 | 5.68102E-07 | 5.39619E-05 | yes | up | 185.69 | 150.81 | 190.95 | 311.85 | 445.53 | 313.89 | 175.8166667 | 357.09 |
| gene20105 | COL6A2 | collagen, type VI, alpha 2, transcript variant X1 | 2.207 | 1.141827042 | 0.000586057 | 0.018555808 | yes | up | 541.5 | 402.02 | 914.13 | 987.15 | 1274.31 | 1476.79 | 619.2166667 | 1246.083333 |
| gene2462 | JAZF1 | JAZF zinc finger 1, transcript variant X1 | 2.197 | 1.135389939 | 7.62051E-09 | 1.0895E-06 | yes | up | 7.73 | 7.76 | 6.81 | 16.98 | 14.84 | 12.9 | 7.433333333 | 14.90666667 |
| gene7273 | GXYLT2 | glucoside xylosyltransferase 2, transcript variant X1 | 2.192 | 1.132291799 | 7.24008E-08 | 8.73091E-06 | yes | up | 58.59 | 44.93 | 36.85 | 100.57 | 89.49 | 98.83 | 46.79 | 96.29666667 |
| gene8234 | ROPN1L | rhophilin associated tail protein 1-like, transcript variant X2 | 2.186 | 1.128332091 | 0.001810568 | 0.043667759 | yes | up | 20.97 | 23.08 | 18.96 | 65.76 | 25.69 | 36.31 | 21.00333333 | 42.58666667 |
| gene15109 | ADAMTSL2 | ADAMTS-like 2 | 2.183 | 1.126615879 | 0.000436668 | 0.014627325 | yes | up | 1.45 | 1.78 | 2.84 | 3.78 | 3.46 | 3.8 | 2.023333333 | 3.68 |
| gene16293 | MYL9 | myosin, light chain 9, regulatory | 2.182 | 1.125806042 | 9.84225E-07 | 8.74951E-05 | yes | up | 102.03 | 75.83 | 94.96 | 139.02 | 212.26 | 191.85 | 90.94 | 181.0433333 |
| gene1651 | ACSS3 | acyl-CoA synthetase short-chain family member 3 | 2.181 | 1.125064729 | 0.002218767 | 0.049789412 | yes | up | 3.17 | 3.62 | 3.23 | 5.75 | 9.86 | 4.45 | 3.34 | 6.686666667 |
| gene57 | CPN1 | carboxypeptidase N, polypeptide 1 | 2.18 | 1.12425302 | 0.000292774 | 0.01057342 | yes | up | 8.77 | 5.79 | 6.21 | 14 | 17.92 | 9.48 | 6.923333333 | 13.8 |
| gene19712 | LOC106049199 | bone morphogenetic protein 1-like | 2.173 | 1.119786348 | 0.000355398 | 0.012275148 | yes | up | 5.54 | 6.4 | 7.21 | 9.07 | 17.28 | 11.83 | 6.383333333 | 12.72666667 |
| gene18605 | P3H4 | prolyl 3-hydroxylase family member 4 (non-enzymatic) | 2.171 | 1.118564965 | 0.001684476 | 0.041272648 | yes | up | 5.56 | 2.69 | 3.91 | 5.58 | 10.06 | 8.43 | 4.053333333 | 8.023333333 |
| gene19705 | ASPN | asporin | 2.164 | 1.113614398 | 1.38653E-06 | 0.000117966 | yes | up | 23.97 | 17.79 | 26.01 | 52.53 | 37.18 | 44.41 | 22.59 | 44.70666667 |
| gene11108 | CLMP | CXADR-like membrane protein, transcript variant X6 | 2.157 | 1.108843546 | 2.62151E-08 | 3.38824E-06 | yes | up | 12.72 | 12.97 | 12.2 | 29.76 | 25.96 | 21.31 | 12.63 | 25.67666667 |
| gene15052 | NID2 | nidogen 2 (osteonidogen) | 2.156 | 1.1082845 | 4.97871E-05 | 0.002483624 | yes | up | 40.49 | 32.32 | 48.86 | 57.3 | 98.51 | 83.79 | 40.55666667 | 79.86666667 |
| gene429 | ZCCHC24 | zinc finger, CCHC domain containing 24 | 2.151 | 1.104907006 | 2.14047E-10 | 4.54002E-08 | yes | up | 25.5 | 23.23 | 22.8 | 41.12 | 54.04 | 45.05 | 23.84333333 | 46.73666667 |
| gene6950 | PDGFRL | platelet-derived growth factor receptor-like, transcript variant X1 | 2.149 | 1.103819551 | 1.11799E-10 | 2.54169E-08 | yes | up | 12.96 | 13.34 | 12.44 | 23.13 | 29.39 | 23.36 | 12.91333333 | 25.29333333 |
| gene2590 | COL12A1 | collagen, type XII, alpha 1 | 2.146 | 1.101597137 | 0.000377811 | 0.012873431 | yes | up | 21.97 | 20.64 | 40.19 | 58.6 | 50.23 | 56.59 | 27.6 | 55.14 |
| gene12616 | LRRC75A | leucine rich repeat containing 75A | 2.141 | 1.098126136 | 0.000376451 | 0.012858691 | yes | up | 5.28 | 5.5 | 7.17 | 10.02 | 11.5 | 15.73 | 5.983333333 | 12.41666667 |
| gene16758 | MRC2 | mannose receptor, C type 2 | 2.14 | 1.09768457 | 0.001555308 | 0.038933237 | yes | up | 33.59 | 56.5 | 23.32 | 57.27 | 88.02 | 76.19 | 37.80333333 | 73.82666667 |
| gene20106 | COL6A1 | collagen, type VI, alpha 1 | 2.14 | 1.097290642 | 0.000563973 | 0.017938473 | yes | up | 414.52 | 299.48 | 649.26 | 696.67 | 921.3 | 1040.38 | 454.42 | 886.1166667 |
| gene10892 | EPHX4 | epoxide hydrolase 4 | 2.136 | 1.095011488 | 0.000616015 | 0.01911162 | yes | up | 29.13 | 19.04 | 17.68 | 55.55 | 28.12 | 42.3 | 21.95 | 41.99 |
| gene14773 | PITPNM3 | PITPNM family member 3, transcript variant X4 | 2.136 | 1.094644752 | 0.00048807 | 0.01615407 | yes | up | 2.23 | 2.85 | 2.55 | 3.44 | 7.02 | 4.34 | 2.543333333 | 4.933333333 |
| gene19214 | TMED3 | transmembrane emp24 protein transport domain containing 3 | 2.136 | 1.094973566 | 0.000131871 | 0.005508391 | yes | up | 12.57 | 23.61 | 18.56 | 34.81 | 36.71 | 36 | 18.24666667 | 35.84 |
| gene16134 | FBN2 | fibrillin 2, transcript variant X1 | 2.135 | 1.094515403 | 0.002167054 | 0.049082747 | yes | up | 15.72 | 14.1 | 17.7 | 12.5 | 12.67 | 11.25 | 15.84 | 12.14 |
| gene47 | HPSE2 | heparanase 2 (inactive) | 2.135 | 1.094514698 | 0.001454224 | 0.037092301 | yes | up | 16.96 | 41.24 | 18.65 | 55.66 | 38.94 | 56.33 | 25.61666667 | 50.31 |
| gene16785 | RAB26 | RAB26, member RAS oncogene family | 2.132 | 1.091992918 | 8.99297E-05 | 0.003973435 | yes | up | 6.87 | 6.3 | 7.71 | 14.28 | 10.49 | 14.72 | 6.96 | 13.16333333 |
| gene19637 | LOC106049116 | protein S100-A4-like | 2.131 | 1.0917798 | 5.28921E-05 | 0.002573709 | yes | up | 1088.1 | 1395.88 | 1713.84 | 3390.93 | 2115.66 | 2976.43 | 1399.273333 | 2827.673333 |
| gene15995 | S1PR3 | sphingosine-1-phosphate receptor 3, transcript variant X1 | 2.13 | 1.090832756 | 3.03193E-05 | 0.001668522 | yes | up | 23.93 | 15.51 | 13.64 | 36.32 | 37.69 | 28.06 | 17.69333333 | 34.02333333 |
| gene13196 | FOXP2 | forkhead box P2 | 2.123 | 1.086074881 | 7.19732E-06 | 0.000477417 | yes | up | 1.81 | 2.44 | 1.73 | 4.09 | 3.74 | 3.44 | 1.993333333 | 3.756666667 |
| gene7851 | DSCR3 | Down syndrome critical region 3 | 2.122 | 1.085501586 | 0.000239068 | 0.009058453 | yes | up | 7.71 | 9.25 | 11.63 | 24.22 | 16.41 | 15.21 | 9.53 | 18.61333333 |
| gene3409 | ADGRD1 | adhesion G protein-coupled receptor D1, transcript variant X1 | 2.12 | 1.084005667 | 0.000298957 | 0.010740779 | yes | up | 12.8 | 11.62 | 10.17 | 12.18 | 13.83 | 11.57 | 11.53 | 12.52666667 |
| gene15269 | SAMD11 | sterile alpha motif domain containing 11 | 2.114 | 1.079869446 | 3.17367E-05 | 0.001732773 | yes | up | 4.91 | 4.63 | 3.2 | 5.43 | 10.89 | 7.64 | 4.246666667 | 7.986666667 |
| gene20733 | LOC106029601 | butyrophilin subfamily 1 member A1-like | 2.111 | 1.077724158 | 0.001250748 | 0.033101863 | yes | up | 29.84 | 15.72 | 36.69 | 47.49 | 53.78 | 59.68 | 27.41666667 | 53.65 |
| gene18040 | LOC106047369 | uncharacterized LOC106047369 | 2.11 | 1.077097462 | 0.000180887 | 0.007126537 | yes | up | 7.08 | 6.01 | 4.63 | 13.21 | 15.62 | 8.32 | 5.906666667 | 12.38333333 |
| gene10723 | HRASLS | HRAS-like suppressor, transcript variant X1 | 2.103 | 1.072704969 | 0.000111641 | 0.004749184 | yes | up | 2.89 | 2.52 | 2.73 | 4.62 | 6.11 | 4.94 | 2.713333333 | 5.223333333 |
| gene13284 | SRPX | sushi-repeat containing protein, X-linked | 2.102 | 1.071602027 | 0.000597588 | 0.018749671 | yes | up | 21.45 | 31.17 | 15.65 | 38.13 | 33.26 | 45.73 | 22.75666667 | 39.04 |
| gene18771 | LOC106048174 | protein transport protein Sec24D-like, transcript variant X3 | 2.102 | 1.071524299 | 5.3278E-05 | 0.002583426 | yes | up | 15.27 | 21.47 | 21.42 | 28.73 | 42.38 | 41.89 | 19.38666667 | 37.66666667 |
| gene5711 | DAPK2 | death-associated protein kinase 2 | 2.101 | 1.071404569 | 0.001770176 | 0.042992636 | yes | up | 2.04 | 1.49 | 2.05 | 4.94 | 4.18 | 2.11 | 1.86 | 3.743333333 |
| gene15095 | GBGT1 | globoside alpha-1,3-N-acetylgalactosaminyltransferase 1, transcript variant X2 | 2.088 | 1.062416169 | 1.98485E-05 | 0.001159146 | yes | up | 3.39 | 3.3 | 4.05 | 6.07 | 8.68 | 5.7 | 3.58 | 6.816666667 |
| gene3028 | PDGFRA | platelet-derived growth factor receptor, alpha polypeptide, transcript variant X1 | 2.087 | 1.061435548 | 2.19876E-05 | 0.001255418 | yes | up | 30.8 | 30.93 | 21.5 | 50.03 | 66.39 | 42.13 | 27.74333333 | 52.85 |
| gene4515 | EPB41L2 | erythrocyte membrane protein band 4.1-like 2, transcript variant X3 | 2.084 | 1.05938789 | 3.84367E-05 | 0.002026767 | yes | up | 30.97 | 36.43 | 23.01 | 52.1 | 71.8 | 47.4 | 30.13666667 | 57.1 |
| gene12708 | LOC106041506 | uncharacterized LOC106041506 | 2.082 | 1.057817361 | 7.40277E-05 | 0.003456618 | yes | up | 4.82 | 4.68 | 4.21 | 9.05 | 10.87 | 6.3 | 4.57 | 8.74 |
| gene11073 | FZD1 | frizzled class receptor 1 | 2.081 | 1.057461376 | 0.000142653 | 0.005801506 | yes | up | 45.15 | 70.35 | 66.13 | 80.04 | 90.43 | 131.9 | 60.54333333 | 100.79 |
| gene7137 | TBX18 | T-box 18 | 2.073 | 1.051981631 | 3.54099E-06 | 0.000269908 | yes | up | 7.04 | 8.79 | 6.12 | 12.27 | 13.28 | 15.94 | 7.316666667 | 13.83 |
| gene18084 | CPEB1 | cytoplasmic polyadenylation element binding protein 1, transcript variant X1 | 2.068 | 1.048477821 | 0.001656383 | 0.04080057 | yes | up | 1.94 | 3.21 | 3.87 | 5.42 | 4.8 | 6.83 | 3.006666667 | 5.683333333 |
| gene18779 | BNC2 | basonuclin 2 | 2.066 | 1.046696479 | 0.000516477 | 0.016864591 | yes | up | 1.98 | 3.98 | 2.98 | 5.41 | 4.94 | 6.72 | 2.98 | 5.69 |
| gene10105 | SEC24D | SEC24 family member D | 2.064 | 1.045353682 | 5.73469E-11 | 1.39524E-08 | yes | up | 21.97 | 20.27 | 24.97 | 50.55 | 53.6 | 38.97 | 22.40333333 | 47.70666667 |
| gene4754 | P4HA2 | prolyl 4-hydroxylase, alpha polypeptide II, transcript variant X6 | 2.063 | 1.044726403 | 1.33424E-09 | 2.40302E-07 | yes | up | 18.33 | 18.48 | 19.57 | 33.22 | 42.14 | 33.87 | 18.79333333 | 36.41 |
| gene2665 | LOC106030435 | glutathione S-transferase 3 | 2.061 | 1.043606265 | 0.000426266 | 0.014313458 | yes | up | 56.39 | 67.76 | 39.22 | 95.76 | 83.22 | 138.71 | 54.45666667 | 105.8966667 |
| gene2731 | GEM | GTP binding protein overexpressed in skeletal muscle | 2.059 | 1.041859032 | 1.2417E-07 | 1.43499E-05 | yes | up | 46.99 | 39.76 | 54.93 | 82.42 | 85.37 | 99.07 | 47.22666667 | 88.95333333 |
| gene10846 | LOC106039426 | lipid phosphate phosphatase-related protein type 5 | 2.058 | 1.04130966 | 0.000421478 | 0.014214594 | yes | up | 4.96 | 4.28 | 3.61 | 10.82 | 5.91 | 7.43 | 4.283333333 | 8.053333333 |
| gene1064 | PTGFRN | prostaglandin F2 receptor inhibitor | 2.042 | 1.029639802 | 2.94249E-09 | 4.91644E-07 | yes | up | 10.87 | 12.83 | 11.88 | 18.93 | 24.65 | 22.91 | 11.86 | 22.16333333 |
| gene16342 | FKBP9 | FK506 binding protein 9, 63 kDa | 2.029 | 1.020753085 | 1.47103E-06 | 0.000123638 | yes | up | 133.77 | 172.24 | 134.21 | 236.11 | 326.88 | 254.55 | 146.74 | 272.5133333 |
| gene15897 | LOC106044932 | perilipin-3-like | 2.022 | 1.015522649 | 2.20287E-05 | 0.001255418 | yes | up | 339.95 | 231.51 | 358.5 | 603.62 | 471.45 | 648.46 | 309.9866667 | 574.51 |
| gene11760 | PSD3 | pleckstrin and Sec7 domain containing 3, transcript variant X1 | 2.008 | 1.00566285 | 0.000681579 | 0.0208197 | yes | up | 7.78 | 12.77 | 6.39 | 18.49 | 17.48 | 14.56 | 8.98 | 16.84333333 |
| gene4586 | DSE | dermatan sulfate epimerase | 2.002 | 1.001427518 | 0.000315796 | 0.011229398 | yes | up | 6.19 | 5.73 | 5.26 | 7.23 | 10.45 | 13.71 | 5.726666667 | 10.46333333 |
| gene7510 | PRKX | protein kinase, X-linked, transcript variant X3 | 0.497 | -1.008520852 | 0.001976736 | 0.04646334 | yes | down | 13.92 | 18.11 | 14.11 | 6.85 | 4.29 | 9.91 | 15.38 | 7.016666667 |
| gene6699 | FAM134B | family with sequence similarity 134, member B, transcript variant X1 | 0.492 | -1.023087926 | 8.46296E-05 | 0.00381053 | yes | down | 7.72 | 10.26 | 6.18 | 4.06 | 2.74 | 2.74 | 8.053333333 | 3.18 |
| gene15651 | LGALSL | lectin, galactoside-binding-like | 0.462 | -1.112484333 | 4.08698E-05 | 0.002118764 | yes | down | 41.77 | 60.65 | 45.11 | 23.95 | 14.8 | 23.53 | 49.17666667 | 20.76 |
| gene7327 | SEMA3A | sema domain, immunoglobulin domain (Ig), short basic domain, secreted, (semaphorin) 3A, transcript variant X1 | 0.462 | -1.115192393 | 0.002094615 | 0.047934196 | yes | down | 3.45 | 3.72 | 2.69 | 1.38 | 0.8 | 1.97 | 3.286666667 | 1.383333333 |
| gene20307 | LOC106049814 | uncharacterized LOC106049814, transcript variant X1 | 0.458 | -1.125987709 | 0.000158562 | 0.006355301 | yes | down | 10.34 | 13.28 | 17.69 | 5.77 | 6.06 | 7.81 | 13.77 | 6.546666667 |
| gene9866 | CXCR6 | chemokine (C-X-C motif) receptor 6 | 0.458 | -1.125220892 | 0.000588959 | 0.018562918 | yes | down | 12.2 | 13.7 | 6.84 | 5.5 | 4.65 | 3.12 | 10.91333333 | 4.423333333 |
| gene11726 | ARHGAP20 | Rho GTPase activating protein 20 | 0.453 | -1.141365203 | 0.000269909 | 0.009876255 | yes | down | 4.57 | 6.04 | 3.41 | 2.33 | 1.52 | 1.95 | 4.673333333 | 1.933333333 |
| gene17469 | DNAH17 | dynein, axonemal, heavy chain 17 | 0.442 | -1.17850823 | 0.000976127 | 0.027570107 | yes | down | 29.25 | 27.6 | 34.09 | 32.47 | 24.3 | 21.38 | 30.31333333 | 26.05 |
| gene6524 | TOM1L1 | target of myb1 like 1 membrane trafficking protein, transcript variant X3 | 0.418 | -1.258242759 | 0.001524162 | 0.038361295 | yes | down | 14.63 | 22.58 | 13 | 7.08 | 1.67 | 5.91 | 16.73666667 | 4.886666667 |
| gene3493 | LOC106031290 | uncharacterized LOC106031290 | 0.416 | -1.266072095 | 7.96091E-05 | 0.003667838 | yes | down | 104.48 | 51.35 | 84.2 | 31.99 | 29.99 | 32.69 | 80.01 | 31.55666667 |
| gene14098 | SLC46A2 | solute carrier family 46, member 2 | 0.413 | -1.27484159 | 0.002107352 | 0.048067045 | yes | down | 11.53 | 20.7 | 22.48 | 7.61 | 1.86 | 8.72 | 18.23666667 | 6.063333333 |
| gene10348 | INSIG1 | insulin induced gene 1 | 0.405 | -1.302677519 | 0.000289989 | 0.010500177 | yes | down | 162.61 | 211.96 | 117.4 | 76.63 | 32.95 | 70.2 | 163.99 | 59.92666667 |
| gene16029 | LOC106045149 | connective tissue growth factor-like, transcript variant X10 | 0.404 | -1.307580039 | 7.88945E-06 | 0.000516089 | yes | down | 192.12 | 221 | 204.62 | 91.57 | 91.65 | 44.92 | 205.9133333 | 76.04666667 |
| gene14822 | DUSP14 | dual specificity phosphatase 14, transcript variant X1 | 0.399 | -1.326565415 | 1.32475E-06 | 0.000113405 | yes | down | 32.71 | 48.41 | 52.31 | 17.25 | 12.24 | 19.05 | 44.47666667 | 16.18 |
| gene14844 | LURAP1L | leucine rich adaptor protein 1-like | 0.385 | -1.37866244 | 0.002035068 | 0.047404387 | yes | down | 13.11 | 16.98 | 15.96 | 5.23 | 2.72 | 9.15 | 15.35 | 5.7 |
| gene18845 | ALDH3B1 | aldehyde dehydrogenase 3 family, member B1 | 0.372 | -1.426748572 | 0.00026378 | 0.009754945 | yes | down | 9.16 | 22.57 | 13.81 | 6.64 | 4 | 5.13 | 15.18 | 5.256666667 |
| gene4527 | MOXD1 | monooxygenase, DBH-like 1 | 0.363 | -1.461147874 | 1.67518E-09 | 2.94069E-07 | yes | down | 14.04 | 21.23 | 19.88 | 5.83 | 5.56 | 6.92 | 18.38333333 | 6.103333333 |
| gene10475 | LOC106039066 | uncharacterized LOC106039066, transcript variant X1 | 0.362 | -1.467727239 | 0.001309461 | 0.034198886 | yes | down | 2.09 | 3.2 | 2.36 | 1.03 | 0.47 | 1.04 | 2.55 | 0.846666667 |
| gene10735 | CXCR4 | chemokine (C-X-C motif) receptor 4 | 0.348 | -1.522940718 | 1.10208E-05 | 0.00067927 | yes | down | 16.36 | 31.85 | 25.19 | 10.18 | 5.41 | 7.87 | 24.46666667 | 7.82 |
| gene16232 | LOC106045365 | fructose-1,6-bisphosphatase 1-like | 0.343 | -1.542050501 | 6.65512E-05 | 0.003171588 | yes | down | 14.97 | 35.45 | 21.27 | 9.48 | 5.49 | 7.68 | 23.89666667 | 7.55 |
| gene15810 | TNFSF13B | tumor necrosis factor (ligand) superfamily, member 13b | 0.324 | -1.626220517 | 0.000979191 | 0.027575864 | yes | down | 5.08 | 11.62 | 10.12 | 4.54 | 1.3 | 2.16 | 8.94 | 2.666666667 |
| gene10214 | LOC106038747 | endonuclease domain-containing 1 protein-like, transcript variant X1 | 0.316 | -1.661466161 | 0.00030402 | 0.010866347 | yes | down | 2.15 | 3.74 | 4.29 | 1.46 | 0.98 | 0.68 | 3.393333333 | 1.04 |
| gene20406 | LOC106049923 | uncharacterized LOC106049923 | 0.304 | -1.716287372 | 0.000135092 | 0.005609148 | yes | down | 147.2 | 61.41 | 110.74 | 39.97 | 20.14 | 28.9 | 106.45 | 29.67 |
| gene17837 | LOC106047166 | uncharacterized LOC106047166 | 0.292 | -1.777905942 | 0.000441322 | 0.014747614 | yes | down | 27.7 | 66.58 | 66.46 | 15.91 | 9.33 | 20.7 | 53.58 | 15.31333333 |
| gene4619 | SLC22A16 | solute carrier family 22 (organic cation/carnitine transporter), member 16, transcript variant X1 | 0.284 | -1.817206052 | 0.000116649 | 0.004931986 | yes | down | 0.52 | 0.42 | 0.48 | 0.11 | 0.13 | 0.14 | 0.473333333 | 0.126666667 |
| gene12056 | TNFRSF6B | tumor necrosis factor receptor superfamily, member 6b, decoy | 0.283 | -1.823142848 | 0.001085095 | 0.029564049 | yes | down | 8.4 | 16.13 | 16.47 | 2.87 | 1.24 | 6.51 | 13.66666667 | 3.54 |
| gene2667 | LOC106030438 | glutathione S-transferase-like | 0.278 | -1.84518415 | 0.00084259 | 0.024600093 | yes | down | 8.18 | 24.09 | 39.91 | 8.39 | 3.87 | 6.35 | 24.06 | 6.203333333 |
| gene497 | CD8A | CD8a molecule | 0.268 | -1.902107506 | 0.000148352 | 0.00601561 | yes | down | 1.68 | 4.84 | 3.63 | 0.98 | 0.9 | 0.6 | 3.383333333 | 0.826666667 |
| gene16539 | FAM46C | family with sequence similarity 46, member C | 0.267 | -1.903997765 | 4.50562E-05 | 0.002288787 | yes | down | 2.5 | 3.32 | 6.22 | 1.58 | 0.69 | 0.68 | 4.013333333 | 0.983333333 |
| gene20398 | LOC106049913 | Ig mu chain C region-like | 0.262 | -1.930523342 | 2.01062E-07 | 2.21296E-05 | yes | down | 54.36 | 66.66 | 114.29 | 21.73 | 24.61 | 15.41 | 78.43666667 | 20.58333333 |
| gene9354 | MAP3K15 | mitogen-activated protein kinase kinase kinase 15, transcript variant X18 | 0.254 | -1.978233314 | 0.000108503 | 0.004644199 | yes | down | 3.46 | 6.77 | 2.52 | 0.4 | 1.34 | 0.6 | 4.25 | 0.78 |
| gene2852 | MSC | musculin | 0.244 | -2.034328064 | 3.02264E-05 | 0.001668522 | yes | down | 6.57 | 2.37 | 7.2 | 1.02 | 1.48 | 1.1 | 5.38 | 1.2 |
| gene17135 | PDCD1LG2 | programmed cell death 1 ligand 2, transcript variant X1 | 0.222 | -2.171430194 | 3.02214E-05 | 0.001668522 | yes | down | 2.22 | 2.07 | 3.04 | 0.66 | 0.33 | 0.45 | 2.443333333 | 0.48 |
| gene5876 | TMEM125 | transmembrane protein 125 | 0.214 | -2.224540151 | 0.002064513 | 0.047599814 | yes | down | 1.62 | 2.22 | 2.03 | 0.43 | 0.04 | 0.67 | 1.956666667 | 0.38 |
| gene5537 | TMEM184A | transmembrane protein 184A, transcript variant X2 | 0.209 | -2.260598695 | 0.001668431 | 0.041024481 | yes | down | 0.93 | 0.94 | 0.47 | 0.23 | 0.04 | 0.17 | 0.78 | 0.146666667 |
| gene6009 | LOC106034204 | interstitial collagenase-like | 0.208 | -2.267510459 | 0.001101198 | 0.029768839 | yes | down | 10.56 | 38.5 | 7.35 | 2.29 | 1.59 | 6.85 | 18.80333333 | 3.576666667 |
| gene9019 | SLC6A14 | solute carrier family 6 (amino acid transporter), member 14, transcript variant X1 | 0.205 | -2.286567499 | 5.5819E-07 | 5.3386E-05 | yes | down | 15.85 | 23.21 | 11.64 | 4.86 | 1.31 | 3.26 | 16.9 | 3.143333333 |
| gene3487 | LOC106031283 | Ig lambda chain V-I region BL2-like isoform X50 | 0.199 | -2.326030758 | 3.00672E-06 | 0.00023827 | yes | down | 1902.9 | 3432.43 | 5212.27 | 1191.12 | 342.62 | 465.99 | 3515.866667 | 666.5766667 |
| gene3228 | SPIC | Spi-C transcription factor (Spi-1/PU.1 related), transcript variant X3 | 0.189 | -2.40080477 | 0.00145502 | 0.037092301 | yes | down | 2.84 | 0.81 | 11.6 | 0.86 | 0.82 | 0.97 | 5.083333333 | 0.883333333 |
| gene14957 | JCHAIN | joining chain of multimeric IgA and IgM | 0.187 | -2.416548205 | 4.90554E-09 | 7.39456E-07 | yes | down | 295.15 | 500.57 | 570.87 | 129.94 | 46.65 | 59.06 | 455.53 | 78.55 |
| gene20397 | LOC106049912 | uncharacterized LOC106049912 | 0.184 | -2.440499699 | 2.2853E-06 | 0.000186426 | yes | down | 115.97 | 113.96 | 193.64 | 44.42 | 13.1 | 12.12 | 141.19 | 23.21333333 |
| gene3494 | MMP11 | matrix metallopeptidase 11 | 0.179 | -2.481995399 | 3.73008E-09 | 5.94583E-07 | yes | down | 23.61 | 25.63 | 11.04 | 4 | 4.09 | 1.77 | 20.09333333 | 3.286666667 |
| gene11540 | LOC106040180 | arylacetamide deacetylase-like 4, transcript variant X1 | 0.174 | -2.526987312 | 5.25746E-06 | 0.000377774 | yes | down | 2.24 | 6.89 | 3.2 | 0.92 | 0.44 | 0.49 | 4.11 | 0.616666667 |
| gene4037 | PLEKHS1 | pleckstrin homology domain containing, family S member 1, transcript variant X2 | 0.17 | -2.558065359 | 0.000164428 | 0.006552541 | yes | down | 1.47 | 0.59 | 2.8 | 0.18 | 0.15 | 0.34 | 1.62 | 0.223333333 |
| gene6185 | MZB1 | marginal zone B and B1 cell-specific protein | 0.153 | -2.711094082 | 0.000272472 | 0.009943807 | yes | down | 5.07 | 4.77 | 26.69 | 1.75 | 1.87 | 1.62 | 12.17666667 | 1.746666667 |
| gene20396 | LOC106049916 | Ig heavy chain V region 914-like | 0.136 | -2.874953973 | 3.69004E-09 | 5.94583E-07 | yes | down | 186.98 | 246.78 | 486.41 | 77.44 | 21.78 | 38.14 | 306.7233333 | 45.78666667 |
| gene13153 | GPR22 | G protein-coupled receptor 22 | 0.126 | -2.983402842 | 0.00129634 | 0.033984205 | yes | down | 0.16 | 0.92 | 0.72 | 0.07 | 0.07 | 0.08 | 0.6 | 0.073333333 |
| gene20401 | LOC106049915 | Ig mu heavy chain disease protein-like | 0.122 | -3.038614679 | 2.9165E-09 | 4.91644E-07 | yes | down | 682.56 | 1099.1 | 1822.73 | 251.57 | 74.55 | 81.61 | 1201.463333 | 135.91 |
| gene19237 | LOC106048678 | keratin, type II cytoskeletal 5-like | 0.113 | -3.144440397 | 0.000780889 | 0.02333917 | yes | down | 13.02 | 8.08 | 2.11 | 2.03 | 0.09 | 0.28 | 7.736666667 | 0.8 |
| gene14433 | LOC106043396 | uncharacterized LOC106043396 | 0.111 | -3.171005403 | 9.5504E-10 | 1.76593E-07 | yes | down | 4.63 | 4.93 | 13.54 | 0.95 | 0.88 | 0.56 | 7.7 | 0.796666667 |
| gene14316 | LOC106043258 | uncharacterized LOC106043258 | 0.106 | -3.240467864 | 0.001191948 | 0.031849573 | yes | down | 0.2 | 2.35 | 0.25 | 0.14 | 0.07 | 0.09 | 0.933333333 | 0.1 |
| gene10169 | LOC106038763 | carboxypeptidase A2-like, transcript variant X1 | 0.092 | -3.4473805 | 1.88691E-07 | 2.09341E-05 | yes | down | 13.91 | 25.47 | 17.61 | 2.11 | 0.19 | 2.52 | 18.99666667 | 1.606666667 |
| gene19236 | LOC106048692 | keratin, type II cytoskeletal-like, transcript variant X1 | 0.082 | -3.605005534 | 8.57547E-05 | 0.003848694 | yes | down | 4.7 | 1.68 | 0.81 | 0.46 | 0.1 | 0.05 | 2.396666667 | 0.203333333 |
| gene19238 | LOC106048695 | uncharacterized LOC106048695 | 0.079 | -3.666572555 | 4.31653E-05 | 0.002200793 | yes | down | 6.11 | 4 | 1.49 | 0.6 | 0 | 0.23 | 3.866666667 | 0.276666667 |
| gene19845 | LOC106049327 | growth-regulated alpha protein-like | 0.044 | -4.511999059 | 0.001027206 | 0.028320653 | yes | down | 1.58 | 0.11 | 10.46 | 0.28 | 0.1 | 0.11 | 4.05 | 0.163333333 |
| gene19734 | LOC106049216 | uncharacterized protein DDB_G0274171-like | 0.043 | -4.528672561 | 0.001880047 | 0.044952569 | yes | down | 2.47 | 17.94 | 10.46 | 0.32 | 0 | 0.91 | 10.29 | 0.41 |
| gene19739 | LOC106049217 | scale keratin-like | 0.016 | -5.992402253 | 5.87588E-06 | 0.000407434 | yes | down | 1.44 | 4.81 | 8.1 | 0.32 | 0 | 0 | 4.783333333 | 0.106666667 |
| gene19733 | LOC106049224 | protein TsetseEP-like | 0.006 | -7.389584843 | 6.19656E-06 | 0.000425415 | yes | down | 0.19 | 0.67 | 2.49 | 0 | 0 | 0 | 1.116666667 | 0 |

Note: (1) Gene ID (2) Gene name;(3)The information of Gene description;(4) Fc (L / S): the differential multiple of the gene between the two samples;(5) Log_2_Fc (L/ S) : the logarithm value of the difference multiple base 2 between the two samples of the gene;(6) P-value: the test result of the significance difference of the gene between the two samples;(7) P-adjust: p value adjusted after multiple tests;(8) Significant: whether the difference of the gene between the two samples is Significant, yes is Significant;(9) Regulate Up down-regulation, S2 is the control, up is up-regulation, down is down-regulation;(10) The other columns represent the expression level of the gene in each sample and each group (TPM).

| Table S4. GO terms | | | | | | | |
| --- | --- | --- | --- | --- | --- | --- | --- |
| Number | GO ID | Term Type | Description | DTG/DAG | BTG/BTG | P-value | P-adjust |
| 7 | GO:0044420 | CC | extracellular matrix component | 7/284 | 10/12055 | 3.14701E-08 | 0.000440487 |
| 9 | GO:0005201 | MF | extracellular matrix structural constituent | 9/284 | 37/12055 | 1.3738E-07 | 0.001922912 |
| 10 | GO:0043062 | BP | extracellular structure organization | 10/284 | 50/12055 | 2.0089E-07 | 0.002811863 |
| 18 | GO:0031012 | CC | extracellular matrix | 18/284 | 107/12055 | 2.01266E-07 | 0.002817114 |
| 10 | GO:0030198 | BP | extracellular matrix organization | 10/284 | 42/12055 | 4.44061E-07 | 0.00621552 |
| 12 | GO:0062023 | CC | collagen-containing extracellular matrix | 12/284 | 55/12055 | 5.15511E-07 | 0.007215607 |
| 48 | GO:0044421 | CC | extracellular region part | 48/284 | 466/12055 | 5.5893E-07 | 0.007823342 |
| 18 | GO:0005581 | CC | collagen trimer | 18/284 | 56/12055 | 6.14666E-07 | 0.008603487 |
| 36 | GO:0005615 | CC | extracellular space | 36/284 | 327/12055 | 8.18336E-07 | 0.011454249 |
| 31 | GO:0005576 | CC | extracellular region | 31/284 | 330/12055 | 9.63862E-07 | 0.013491179 |
| 6 | GO:0005518 | MF | collagen binding | 6/284 | 16/12055 | 1.06482E-06 | 0.01490422 |
| 9 | GO:0005539 | MF | glycosaminoglycan binding | 9/284 | 48/12055 | 1.4756E-06 | 0.020653947 |
| 4 | GO:0005583 | CC | fibrillar collagen trimer | 4/284 | 5/12055 | 1.48057E-06 | 0.020723543 |
| 4 | GO:0090130 | BP | tissue migration | 4/284 | 6/12055 | 4.35921E-06 | 0.061015853 |
| 4 | GO:0030199 | BP | collagen fibril organization | 4/284 | 8/12055 | 1.95951E-05 | 0.274272477 |
| 6 | GO:0008201 | MF | heparin binding | 6/284 | 27/12055 | 3.16341E-05 | 0.44278191 |
| 5 | GO:0016525 | BP | negative regulation of angiogenesis | 5/284 | 21/12055 | 0.000104573 | 1 |
| 3 | GO:0090131 | BP | mesenchyme migration | 3/284 | 5/12055 | 0.000124924 | 1 |
| 5 | GO:2000181 | BP | negative regulation of blood vessel morphogenesis | 5/284 | 22/12055 | 0.000132739 | 1 |
| 5 | GO:1901343 | BP | negative regulation of vasculature development | 5/284 | 23/12055 | 0.000166367 | 1 |
| 7 | GO:0045765 | BP | regulation of angiogenesis | 7/284 | 51/12055 | 0.000178242 | 1 |
| 5 | GO:0001501 | BP | skeletal system development | 5/284 | 24/12055 | 0.000206131 | 1 |
| 7 | GO:1901342 | BP | regulation of vasculature development | 7/284 | 55/12055 | 0.00028833 | 1 |
| 3 | GO:0032461 | BP | positive regulation of protein oligomerization | 3/284 | 7/12055 | 0.000422118 | 1 |
| 13 | GO:0022603 | BP | regulation of anatomical structure morphogenesis | 13/284 | 186/12055 | 0.00046056 | 1 |
| 2 | GO:0072378 | BP | blood coagulation, fibrin clot formation | 2/284 | 2/12055 | 0.000553103 | 1 |
| 2 | GO:0045725 | BP | positive regulation of glycogen biosynthetic process | 2/284 | 2/12055 | 0.000553103 | 1 |
| 2 | GO:0043569 | BP | negative regulation of insulin-like growth factor receptor signaling pathway | 2/284 | 2/12055 | 0.000553103 | 1 |
| 2 | GO:0070875 | BP | positive regulation of glycogen metabolic process | 2/284 | 2/12055 | 0.000553103 | 1 |
| 2 | GO:0072376 | BP | protein activation cascade | 2/284 | 3/12055 | 0.001633429 | 1 |
| 2 | GO:0070208 | BP | protein heterotrimerization | 2/284 | 3/12055 | 0.001633429 | 1 |
| 23 | GO:0050793 | BP | regulation of developmental process | 23/284 | 487/12055 | 0.001829475 | 1 |
| 3 | GO:0006939 | BP | smooth muscle contraction | 3/284 | 12/12055 | 0.00243077 | 1 |
| 3 | GO:0032459 | BP | regulation of protein oligomerization | 3/284 | 12/12055 | 0.00243077 | 1 |
| 4 | GO:0051896 | BP | regulation of protein kinase B signaling | 4/284 | 25/12055 | 0.002581441 | 1 |
| 3 | GO:0051897 | BP | positive regulation of protein kinase B signaling | 3/284 | 13/12055 | 0.003105311 | 1 |
| 2 | GO:1904707 | BP | positive regulation of vascular smooth muscle cell proliferation | 2/284 | 4/12055 | 0.003216 | 1 |
| 2 | GO:1904705 | BP | regulation of vascular smooth muscle cell proliferation | 2/284 | 4/12055 | 0.003216 | 1 |
| 2 | GO:0003414 | BP | chondrocyte morphogenesis involved in endochondral bone morphogenesis | 2/284 | 4/12055 | 0.003216 | 1 |
| 2 | GO:0005979 | BP | regulation of glycogen biosynthetic process | 2/284 | 4/12055 | 0.003216 | 1 |
| 2 | GO:0003429 | BP | growth plate cartilage chondrocyte morphogenesis | 2/284 | 4/12055 | 0.003216 | 1 |
| 2 | GO:0003422 | BP | growth plate cartilage morphogenesis | 2/284 | 4/12055 | 0.003216 | 1 |
| 2 | GO:0090171 | BP | chondrocyte morphogenesis | 2/284 | 4/12055 | 0.003216 | 1 |
| 2 | GO:0032964 | BP | collagen biosynthetic process | 2/284 | 4/12055 | 0.003216 | 1 |
| 2 | GO:0010962 | BP | regulation of glucan biosynthetic process | 2/284 | 4/12055 | 0.003216 | 1 |
| 3 | GO:0051146 | BP | striated muscle cell differentiation | 3/284 | 14/12055 | 0.003883892 | 1 |
| 18 | GO:0023056 | BP | positive regulation of signaling | 18/284 | 366/12055 | 0.00406136 | 1 |
| 4 | GO:0007423 | BP | sensory organ development | 4/284 | 29/12055 | 0.004502158 | 1 |
| 3 | GO:0032963 | BP | collagen metabolic process | 3/284 | 15/12055 | 0.00477104 | 1 |
| 10 | GO:0051093 | BP | negative regulation of developmental process | 10/284 | 162/12055 | 0.005017033 | 1 |
| 4 | GO:0006936 | BP | muscle contraction | 4/284 | 30/12055 | 0.00510008 | 1 |
| 2 | GO:0010907 | BP | positive regulation of glucose metabolic process | 2/284 | 5/12055 | 0.005276717 | 1 |
| 2 | GO:0032885 | BP | regulation of polysaccharide biosynthetic process | 2/284 | 5/12055 | 0.005276717 | 1 |
| 2 | GO:0035459 | BP | vesicle cargo loading | 2/284 | 5/12055 | 0.005276717 | 1 |
| 2 | GO:0046628 | BP | positive regulation of insulin receptor signaling pathway | 2/284 | 5/12055 | 0.005276717 | 1 |
| 2 | GO:0090110 | BP | COPII-coated vesicle cargo loading | 2/284 | 5/12055 | 0.005276717 | 1 |
| 4 | GO:0000904 | BP | cell morphogenesis involved in differentiation | 4/284 | 31/12055 | 0.005748968 | 1 |
| 3 | GO:0046626 | BP | regulation of insulin receptor signaling pathway | 3/284 | 16/12055 | 0.005770781 | 1 |
| 14 | GO:0023057 | BP | negative regulation of signaling | 14/284 | 268/12055 | 0.006086292 | 1 |
| 14 | GO:0010648 | BP | negative regulation of cell communication | 14/284 | 268/12055 | 0.006086292 | 1 |
| 5 | GO:0043122 | BP | regulation of I-kappaB kinase/NF-kappaB signaling | 5/284 | 50/12055 | 0.006244773 | 1 |
| 3 | GO:0043255 | BP | regulation of carbohydrate biosynthetic process | 3/284 | 17/12055 | 0.006886674 | 1 |
| 3 | GO:0042692 | BP | muscle cell differentiation | 3/284 | 17/12055 | 0.006886674 | 1 |
| 8 | GO:0097435 | BP | supramolecular fiber organization | 8/284 | 119/12055 | 0.007078871 | 1 |
| 17 | GO:0010647 | BP | positive regulation of cell communication | 17/284 | 363/12055 | 0.007108079 | 1 |
| 2 | GO:0043567 | BP | regulation of insulin-like growth factor receptor signaling pathway | 2/284 | 6/12055 | 0.007792331 | 1 |
| 2 | GO:0060536 | BP | cartilage morphogenesis | 2/284 | 6/12055 | 0.007792331 | 1 |
| 2 | GO:1900078 | BP | positive regulation of cellular response to insulin stimulus | 2/284 | 6/12055 | 0.007792331 | 1 |
| 26 | GO:0048856 | BP | anatomical structure development | 26/284 | 654/12055 | 0.00783448 | 1 |
| 6 | GO:0048729 | BP | tissue morphogenesis | 6/284 | 74/12055 | 0.007870046 | 1 |
| 44 | GO:0048518 | BP | positive regulation of biological process | 44/284 | 1269/12055 | 0.008073424 | 1 |
| 3 | GO:1900076 | BP | regulation of cellular response to insulin stimulus | 3/284 | 18/12055 | 0.008121831 | 1 |
| 3 | GO:0001649 | BP | osteoblast differentiation | 3/284 | 18/12055 | 0.008121831 | 1 |
| 8 | GO:0043413 | BP | macromolecule glycosylation | 8/284 | 122/12055 | 0.008185224 | 1 |
| 8 | GO:0006486 | BP | protein glycosylation | 8/284 | 122/12055 | 0.008185224 | 1 |
| 8 | GO:0070085 | BP | glycosylation | 8/284 | 124/12055 | 0.008991736 | 1 |
| 3 | GO:0090277 | BP | positive regulation of peptide hormone secretion | 3/284 | 19/12055 | 0.009478945 | 1 |
| 4 | GO:0043123 | BP | positive regulation of I-kappaB kinase/NF-kappaB signaling | 4/284 | 36/12055 | 0.009819513 | 1 |
| 2 | GO:0043588 | BP | skin development | 2/284 | 7/12055 | 0.010740416 | 1 |
| 2 | GO:0010676 | BP | positive regulation of cellular carbohydrate metabolic process | 2/284 | 7/12055 | 0.010740416 | 1 |
| 2 | GO:0033002 | BP | muscle cell proliferation | 2/284 | 7/12055 | 0.010740416 | 1 |
| 2 | GO:0070873 | BP | regulation of glycogen metabolic process | 2/284 | 7/12055 | 0.010740416 | 1 |
| 2 | GO:1901862 | BP | negative regulation of muscle tissue development | 2/284 | 7/12055 | 0.010740416 | 1 |
| 4 | GO:0003012 | BP | muscle system process | 4/284 | 37/12055 | 0.010810188 | 1 |
| 4 | GO:0006022 | BP | aminoglycan metabolic process | 4/284 | 37/12055 | 0.010810188 | 1 |
| 20 | GO:0048584 | BP | positive regulation of response to stimulus | 20/284 | 463/12055 | 0.011047885 | 1 |
| 10 | GO:1902533 | BP | positive regulation of intracellular signal transduction | 10/284 | 187/12055 | 0.013168308 | 1 |
| 24 | GO:0051239 | BP | regulation of multicellular organismal process | 24/284 | 612/12055 | 0.013222723 | 1 |
| 15 | GO:0009967 | BP | positive regulation of signal transduction | 15/284 | 326/12055 | 0.013881768 | 1 |
| 2 | GO:0032881 | BP | regulation of polysaccharide metabolic process | 2/284 | 8/12055 | 0.014099344 | 1 |
| 2 | GO:0045913 | BP | positive regulation of carbohydrate metabolic process | 2/284 | 8/12055 | 0.014099344 | 1 |
| 2 | GO:0010828 | BP | positive regulation of glucose transmembrane transport | 2/284 | 8/12055 | 0.014099344 | 1 |
| 2 | GO:0046326 | BP | positive regulation of glucose import | 2/284 | 8/12055 | 0.014099344 | 1 |
| 2 | GO:0050886 | BP | endocrine process | 2/284 | 8/12055 | 0.014099344 | 1 |
| 2 | GO:0001990 | BP | regulation of systemic arterial blood pressure by hormone | 2/284 | 8/12055 | 0.014099344 | 1 |
| 2 | GO:0006958 | BP | complement activation, classical pathway | 2/284 | 8/12055 | 0.014099344 | 1 |
| 17 | GO:2000026 | BP | regulation of multicellular organismal development | 17/284 | 380/12055 | 0.014179048 | 1 |
| 3 | GO:0010906 | BP | regulation of glucose metabolic process | 3/284 | 22/12055 | 0.014303088 | 1 |
| 12 | GO:0051094 | BP | positive regulation of developmental process | 12/284 | 243/12055 | 0.015467411 | 1 |
| 3 | GO:1901861 | BP | regulation of muscle tissue development | 3/284 | 23/12055 | 0.016167292 | 1 |
| 2 | GO:0048661 | BP | positive regulation of smooth muscle cell proliferation | 2/284 | 9/12055 | 0.017848259 | 1 |
| 2 | GO:0070206 | BP | protein trimerization | 2/284 | 9/12055 | 0.017848259 | 1 |
| 2 | GO:0003044 | BP | regulation of systemic arterial blood pressure mediated by a chemical signal | 2/284 | 9/12055 | 0.017848259 | 1 |
| 7 | GO:0048731 | BP | system development | 7/284 | 114/12055 | 0.01814042 | 1 |
| 3 | GO:0001654 | BP | eye development | 3/284 | 24/12055 | 0.018161356 | 1 |
| 8 | GO:0008284 | BP | positive regulation of cell population proliferation | 8/284 | 141/12055 | 0.018426936 | 1 |
| 12 | GO:0009968 | BP | negative regulation of signal transduction | 12/284 | 252/12055 | 0.018670372 | 1 |
| 26 | GO:0032501 | BP | multicellular organismal process | 26/284 | 697/12055 | 0.019696301 | 1 |
| 3 | GO:0000187 | BP | activation of MAPK activity | 3/284 | 25/12055 | 0.02028589 | 1 |
| 3 | GO:0010675 | BP | regulation of cellular carbohydrate metabolic process | 3/284 | 25/12055 | 0.02028589 | 1 |
| 37 | GO:0048522 | BP | positive regulation of cellular process | 37/284 | 1082/12055 | 0.020349926 | 1 |
| 14 | GO:0048585 | BP | negative regulation of response to stimulus | 14/284 | 316/12055 | 0.021963265 | 1 |
| 2 | GO:0055006 | BP | cardiac cell development | 2/284 | 10/12055 | 0.021967056 | 1 |
| 2 | GO:0046324 | BP | regulation of glucose import | 2/284 | 10/12055 | 0.021967056 | 1 |
| 2 | GO:0048706 | BP | embryonic skeletal system development | 2/284 | 10/12055 | 0.021967056 | 1 |
| 2 | GO:0055013 | BP | cardiac muscle cell development | 2/284 | 10/12055 | 0.021967056 | 1 |
| 2 | GO:0060317 | BP | cardiac epithelial to mesenchymal transition | 2/284 | 10/12055 | 0.021967056 | 1 |
| 2 | GO:0042108 | BP | positive regulation of cytokine biosynthetic process | 2/284 | 10/12055 | 0.021967056 | 1 |
| 1 | GO:0060267 | BP | positive regulation of respiratory burst | 1/284 | 1/12055 | 0.023558689 | 1 |
| 1 | GO:0099641 | BP | anterograde axonal protein transport | 1/284 | 1/12055 | 0.023558689 | 1 |
| 1 | GO:0090132 | BP | epithelium migration | 1/284 | 1/12055 | 0.023558689 | 1 |
| 1 | GO:0003201 | BP | epithelial to mesenchymal transition involved in coronary vasculature morphogenesis | 1/284 | 1/12055 | 0.023558689 | 1 |
| 1 | GO:0044342 | BP | type B pancreatic cell proliferation | 1/284 | 1/12055 | 0.023558689 | 1 |
| 1 | GO:0021502 | BP | neural fold elevation formation | 1/284 | 1/12055 | 0.023558689 | 1 |
| 1 | GO:0034138 | BP | toll-like receptor 3 signaling pathway | 1/284 | 1/12055 | 0.023558689 | 1 |
| 1 | GO:0038033 | BP | positive regulation of endothelial cell chemotaxis by VEGF-activated vascular endothelial growth factor receptor signaling pathway | 1/284 | 1/12055 | 0.023558689 | 1 |
| 1 | GO:0090032 | BP | negative regulation of steroid hormone biosynthetic process | 1/284 | 1/12055 | 0.023558689 | 1 |
| 1 | GO:0014745 | BP | negative regulation of muscle adaptation | 1/284 | 1/12055 | 0.023558689 | 1 |
| 1 | GO:2000542 | BP | negative regulation of gastrulation | 1/284 | 1/12055 | 0.023558689 | 1 |
| 1 | GO:1903766 | BP | positive regulation of potassium ion export across plasma membrane | 1/284 | 1/12055 | 0.023558689 | 1 |
| 1 | GO:0060346 | BP | bone trabecula formation | 1/284 | 1/12055 | 0.023558689 | 1 |
| 1 | GO:1903225 | BP | negative regulation of endodermal cell differentiation | 1/284 | 1/12055 | 0.023558689 | 1 |
| 1 | GO:1903224 | BP | regulation of endodermal cell differentiation | 1/284 | 1/12055 | 0.023558689 | 1 |
| 1 | GO:0032345 | BP | negative regulation of aldosterone metabolic process | 1/284 | 1/12055 | 0.023558689 | 1 |
| 1 | GO:1904204 | BP | regulation of skeletal muscle hypertrophy | 1/284 | 1/12055 | 0.023558689 | 1 |
| 1 | GO:1904205 | BP | negative regulation of skeletal muscle hypertrophy | 1/284 | 1/12055 | 0.023558689 | 1 |
| 1 | GO:0034346 | BP | positive regulation of type III interferon production | 1/284 | 1/12055 | 0.023558689 | 1 |
| 1 | GO:0032348 | BP | negative regulation of aldosterone biosynthetic process | 1/284 | 1/12055 | 0.023558689 | 1 |
| 1 | GO:0014911 | BP | positive regulation of smooth muscle cell migration | 1/284 | 1/12055 | 0.023558689 | 1 |
| 1 | GO:0014912 | BP | negative regulation of smooth muscle cell migration | 1/284 | 1/12055 | 0.023558689 | 1 |
| 1 | GO:0008355 | BP | olfactory learning | 1/284 | 1/12055 | 0.023558689 | 1 |
| 1 | GO:0015858 | BP | nucleoside transport | 1/284 | 1/12055 | 0.023558689 | 1 |
| 1 | GO:2000266 | BP | regulation of blood coagulation, intrinsic pathway | 1/284 | 1/12055 | 0.023558689 | 1 |
| 1 | GO:2000467 | BP | positive regulation of glycogen (starch) synthase activity | 1/284 | 1/12055 | 0.023558689 | 1 |
| 1 | GO:0061033 | BP | secretion by lung epithelial cell involved in lung growth | 1/284 | 1/12055 | 0.023558689 | 1 |
| 1 | GO:0048251 | BP | elastic fiber assembly | 1/284 | 1/12055 | 0.023558689 | 1 |
| 1 | GO:0045163 | BP | clustering of voltage-gated potassium channels | 1/284 | 1/12055 | 0.023558689 | 1 |
| 1 | GO:1903867 | BP | extraembryonic membrane development | 1/284 | 1/12055 | 0.023558689 | 1 |
| 1 | GO:1901642 | BP | nucleoside transmembrane transport | 1/284 | 1/12055 | 0.023558689 | 1 |
| 1 | GO:0016103 | BP | diterpenoid catabolic process | 1/284 | 1/12055 | 0.023558689 | 1 |
| 1 | GO:0072144 | BP | glomerular mesangial cell development | 1/284 | 1/12055 | 0.023558689 | 1 |
| 1 | GO:0072143 | BP | mesangial cell development | 1/284 | 1/12055 | 0.023558689 | 1 |
| 1 | GO:0034344 | BP | regulation of type III interferon production | 1/284 | 1/12055 | 0.023558689 | 1 |
| 1 | GO:1904808 | BP | positive regulation of protein oxidation | 1/284 | 1/12055 | 0.023558689 | 1 |
| 1 | GO:1904806 | BP | regulation of protein oxidation | 1/284 | 1/12055 | 0.023558689 | 1 |
| 1 | GO:0014910 | BP | regulation of smooth muscle cell migration | 1/284 | 1/12055 | 0.023558689 | 1 |
| 1 | GO:0035583 | BP | sequestering of TGFbeta in extracellular matrix | 1/284 | 1/12055 | 0.023558689 | 1 |
| 1 | GO:0032914 | BP | positive regulation of transforming growth factor beta1 production | 1/284 | 1/12055 | 0.023558689 | 1 |
| 1 | GO:0003272 | BP | endocardial cushion formation | 1/284 | 1/12055 | 0.023558689 | 1 |
| 1 | GO:1905069 | BP | allantois development | 1/284 | 1/12055 | 0.023558689 | 1 |
| 1 | GO:2000324 | BP | positive regulation of glucocorticoid receptor signaling pathway | 1/284 | 1/12055 | 0.023558689 | 1 |
| 1 | GO:0060931 | BP | sinoatrial node cell development | 1/284 | 1/12055 | 0.023558689 | 1 |
| 1 | GO:0035989 | BP | tendon development | 1/284 | 1/12055 | 0.023558689 | 1 |
| 1 | GO:0034128 | BP | negative regulation of MyD88-independent toll-like receptor signaling pathway | 1/284 | 1/12055 | 0.023558689 | 1 |
| 1 | GO:0045354 | BP | regulation of interferon-alpha biosynthetic process | 1/284 | 1/12055 | 0.023558689 | 1 |
| 1 | GO:0045357 | BP | regulation of interferon-beta biosynthetic process | 1/284 | 1/12055 | 0.023558689 | 1 |
| 1 | GO:0045356 | BP | positive regulation of interferon-alpha biosynthetic process | 1/284 | 1/12055 | 0.023558689 | 1 |
| 1 | GO:0045359 | BP | positive regulation of interferon-beta biosynthetic process | 1/284 | 1/12055 | 0.023558689 | 1 |
| 1 | GO:1904752 | BP | regulation of vascular associated smooth muscle cell migration | 1/284 | 1/12055 | 0.023558689 | 1 |
| 1 | GO:1904754 | BP | positive regulation of vascular associated smooth muscle cell migration | 1/284 | 1/12055 | 0.023558689 | 1 |
| 1 | GO:0031947 | BP | negative regulation of glucocorticoid biosynthetic process | 1/284 | 1/12055 | 0.023558689 | 1 |
| 1 | GO:0031944 | BP | negative regulation of glucocorticoid metabolic process | 1/284 | 1/12055 | 0.023558689 | 1 |
| 1 | GO:0018057 | BP | peptidyl-lysine oxidation | 1/284 | 1/12055 | 0.023558689 | 1 |
| 1 | GO:0038089 | BP | positive regulation of cell migration by vascular endothelial growth factor signaling pathway | 1/284 | 1/12055 | 0.023558689 | 1 |
| 1 | GO:2000257 | BP | regulation of protein activation cascade | 1/284 | 1/12055 | 0.023558689 | 1 |
| 1 | GO:0002636 | BP | positive regulation of germinal center formation | 1/284 | 1/12055 | 0.023558689 | 1 |
| 1 | GO:2000065 | BP | negative regulation of cortisol biosynthetic process | 1/284 | 1/12055 | 0.023558689 | 1 |
| 1 | GO:0045112 | BP | integrin biosynthetic process | 1/284 | 1/12055 | 0.023558689 | 1 |
| 1 | GO:1905383 | BP | protein localization to presynapse | 1/284 | 1/12055 | 0.023558689 | 1 |
| 1 | GO:0038028 | BP | insulin receptor signaling pathway via phosphatidylinositol 3-kinase | 1/284 | 1/12055 | 0.023558689 | 1 |
| 1 | GO:0030213 | BP | hyaluronan biosynthetic process | 1/284 | 1/12055 | 0.023558689 | 1 |
| 1 | GO:0016115 | BP | terpenoid catabolic process | 1/284 | 1/12055 | 0.023558689 | 1 |
| 1 | GO:0003344 | BP | pericardium morphogenesis | 1/284 | 1/12055 | 0.023558689 | 1 |
| 1 | GO:0034653 | BP | retinoic acid catabolic process | 1/284 | 1/12055 | 0.023558689 | 1 |
| 1 | GO:0031296 | BP | B cell costimulation | 1/284 | 1/12055 | 0.023558689 | 1 |
| 4 | GO:1904062 | BP | regulation of cation transmembrane transport | 4/284 | 47/12055 | 0.024335534 | 1 |
| 14 | GO:0045595 | BP | regulation of cell differentiation | 14/284 | 323/12055 | 0.024610436 | 1 |
| 8 | GO:0007167 | BP | enzyme linked receptor protein signaling pathway | 8/284 | 149/12055 | 0.02472248 | 1 |
| 3 | GO:0009612 | BP | response to mechanical stimulus | 3/284 | 27/12055 | 0.024927429 | 1 |
| 10 | GO:0040012 | BP | regulation of locomotion | 10/284 | 194/12055 | 0.026088573 | 1 |
| 2 | GO:0002260 | BP | lymphocyte homeostasis | 2/284 | 11/12055 | 0.026436352 | 1 |
| 3 | GO:0055001 | BP | muscle cell development | 3/284 | 28/12055 | 0.027444323 | 1 |
| 3 | GO:0055002 | BP | striated muscle cell development | 3/284 | 28/12055 | 0.027444323 | 1 |
| 3 | GO:0046887 | BP | positive regulation of hormone secretion | 3/284 | 28/12055 | 0.027444323 | 1 |
| 13 | GO:0042127 | BP | regulation of cell population proliferation | 13/284 | 290/12055 | 0.027494413 | 1 |
| 3 | GO:0006109 | BP | regulation of carbohydrate metabolic process | 3/284 | 29/12055 | 0.030091496 | 1 |
| 3 | GO:0034764 | BP | positive regulation of transmembrane transport | 3/284 | 29/12055 | 0.030091496 | 1 |
| 2 | GO:0010827 | BP | regulation of glucose transmembrane transport | 2/284 | 12/12055 | 0.031237468 | 1 |
| 2 | GO:1901019 | BP | regulation of calcium ion transmembrane transporter activity | 2/284 | 12/12055 | 0.031237468 | 1 |
| 2 | GO:1901379 | BP | regulation of potassium ion transmembrane transport | 2/284 | 12/12055 | 0.031237468 | 1 |
| 2 | GO:0032760 | BP | positive regulation of tumor necrosis factor production | 2/284 | 12/12055 | 0.031237468 | 1 |
| 2 | GO:0006024 | BP | glycosaminoglycan biosynthetic process | 2/284 | 12/12055 | 0.031237468 | 1 |
| 8 | GO:0043408 | BP | regulation of MAPK cascade | 8/284 | 156/12055 | 0.031352563 | 1 |
| 4 | GO:0062012 | BP | regulation of small molecule metabolic process | 4/284 | 51/12055 | 0.031715518 | 1 |
| 3 | GO:0008360 | BP | regulation of cell shape | 3/284 | 30/12055 | 0.03286832 | 1 |
| 3 | GO:0006959 | BP | humoral immune response | 3/284 | 30/12055 | 0.03286832 | 1 |
| 7 | GO:0008285 | BP | negative regulation of cell population proliferation | 7/284 | 129/12055 | 0.032870863 | 1 |
| 4 | GO:0031334 | BP | positive regulation of protein complex assembly | 4/284 | 52/12055 | 0.033743929 | 1 |
| 3 | GO:0030203 | BP | glycosaminoglycan metabolic process | 3/284 | 31/12055 | 0.035773965 | 1 |
| 2 | GO:0001776 | BP | leukocyte homeostasis | 2/284 | 13/12055 | 0.036352406 | 1 |
| 2 | GO:0018149 | BP | peptide cross-linking | 2/284 | 13/12055 | 0.036352406 | 1 |
| 2 | GO:1903557 | BP | positive regulation of tumor necrosis factor superfamily cytokine production | 2/284 | 13/12055 | 0.036352406 | 1 |
| 4 | GO:0010959 | BP | regulation of metal ion transport | 4/284 | 54/12055 | 0.038022853 | 1 |
| 4 | GO:0032147 | BP | activation of protein kinase activity | 4/284 | 54/12055 | 0.038022853 | 1 |
| 3 | GO:0030177 | BP | positive regulation of Wnt signaling pathway | 3/284 | 32/12055 | 0.038807411 | 1 |
| 9 | GO:0001934 | BP | positive regulation of protein phosphorylation | 9/284 | 178/12055 | 0.039024422 | 1 |
| 10 | GO:0031401 | BP | positive regulation of protein modification process | 10/284 | 218/12055 | 0.039082172 | 1 |
| 6 | GO:0007169 | BP | transmembrane receptor protein tyrosine kinase signaling pathway | 6/284 | 106/12055 | 0.039136898 | 1 |
| 28 | GO:0023051 | BP | regulation of signaling | 28/284 | 804/12055 | 0.039446865 | 1 |
| 4 | GO:0002009 | BP | morphogenesis of an epithelium | 4/284 | 55/12055 | 0.040273654 | 1 |
| 10 | GO:0048513 | BP | animal organ development | 10/284 | 220/12055 | 0.040673802 | 1 |
| 9 | GO:2000145 | BP | regulation of cell motility | 9/284 | 183/12055 | 0.041653121 | 1 |
| 2 | GO:0043010 | BP | camera-type eye development | 2/284 | 14/12055 | 0.041763825 | 1 |
| 2 | GO:0070373 | BP | negative regulation of ERK1 and ERK2 cascade | 2/284 | 14/12055 | 0.041763825 | 1 |
| 2 | GO:0006023 | BP | aminoglycan biosynthetic process | 2/284 | 14/12055 | 0.041763825 | 1 |
| 2 | GO:0042102 | BP | positive regulation of T cell proliferation | 2/284 | 14/12055 | 0.041763825 | 1 |
| 6 | GO:0040017 | BP | positive regulation of locomotion | 6/284 | 108/12055 | 0.04225766 | 1 |
| 7 | GO:0034762 | BP | regulation of transmembrane transport | 7/284 | 138/12055 | 0.044736271 | 1 |
| 9 | GO:0042327 | BP | positive regulation of phosphorylation | 9/284 | 188/12055 | 0.04493453 | 1 |
| 3 | GO:0090288 | BP | negative regulation of cellular response to growth factor stimulus | 3/284 | 34/12055 | 0.045252763 | 1 |
| 3 | GO:0032412 | BP | regulation of ion transmembrane transporter activity | 3/284 | 34/12055 | 0.045252763 | 1 |
| 3 | GO:0022898 | BP | regulation of transmembrane transporter activity | 3/284 | 34/12055 | 0.045252763 | 1 |
| 3 | GO:0050679 | BP | positive regulation of epithelial cell proliferation | 3/284 | 34/12055 | 0.045252763 | 1 |
| 1 | GO:0045078 | BP | positive regulation of interferon-gamma biosynthetic process | 1/284 | 2/12055 | 0.046564275 | 1 |
| 1 | GO:0045072 | BP | regulation of interferon-gamma biosynthetic process | 1/284 | 2/12055 | 0.046564275 | 1 |
| 1 | GO:0014846 | BP | esophagus smooth muscle contraction | 1/284 | 2/12055 | 0.046564275 | 1 |
| 1 | GO:2000312 | BP | regulation of kainate selective glutamate receptor activity | 1/284 | 2/12055 | 0.046564275 | 1 |
| 1 | GO:0035630 | BP | bone mineralization involved in bone maturation | 1/284 | 2/12055 | 0.046564275 | 1 |
| 1 | GO:0009164 | BP | nucleoside catabolic process | 1/284 | 2/12055 | 0.046564275 | 1 |
| 1 | GO:0060926 | BP | cardiac pacemaker cell development | 1/284 | 2/12055 | 0.046564275 | 1 |
| 1 | GO:1990418 | BP | response to insulin-like growth factor stimulus | 1/284 | 2/12055 | 0.046564275 | 1 |
| 1 | GO:1901077 | BP | regulation of relaxation of muscle | 1/284 | 2/12055 | 0.046564275 | 1 |
| 1 | GO:0048305 | BP | immunoglobulin secretion | 1/284 | 2/12055 | 0.046564275 | 1 |
| 1 | GO:0090030 | BP | regulation of steroid hormone biosynthetic process | 1/284 | 2/12055 | 0.046564275 | 1 |
| 1 | GO:0034656 | BP | nucleobase-containing small molecule catabolic process | 1/284 | 2/12055 | 0.046564275 | 1 |
| 1 | GO:1903764 | BP | regulation of potassium ion export across plasma membrane | 1/284 | 2/12055 | 0.046564275 | 1 |
| 1 | GO:1902176 | BP | negative regulation of oxidative stress-induced intrinsic apoptotic signaling pathway | 1/284 | 2/12055 | 0.046564275 | 1 |
| 1 | GO:0060665 | BP | regulation of branching involved in salivary gland morphogenesis by mesenchymal-epithelial signaling | 1/284 | 2/12055 | 0.046564275 | 1 |
| 1 | GO:0030049 | BP | muscle filament sliding | 1/284 | 2/12055 | 0.046564275 | 1 |
| 1 | GO:0032347 | BP | regulation of aldosterone biosynthetic process | 1/284 | 2/12055 | 0.046564275 | 1 |
| 1 | GO:0003094 | BP | glomerular filtration | 1/284 | 2/12055 | 0.046564275 | 1 |
| 1 | GO:0060292 | BP | long-term synaptic depression | 1/284 | 2/12055 | 0.046564275 | 1 |
| 1 | GO:0019731 | BP | antibacterial humoral response | 1/284 | 2/12055 | 0.046564275 | 1 |
| 1 | GO:1900747 | BP | negative regulation of vascular endothelial growth factor signaling pathway | 1/284 | 2/12055 | 0.046564275 | 1 |
| 1 | GO:0031017 | BP | exocrine pancreas development | 1/284 | 2/12055 | 0.046564275 | 1 |
| 1 | GO:0042363 | BP | fat-soluble vitamin catabolic process | 1/284 | 2/12055 | 0.046564275 | 1 |
| 1 | GO:0003050 | BP | regulation of systemic arterial blood pressure by atrial natriuretic peptide | 1/284 | 2/12055 | 0.046564275 | 1 |
| 1 | GO:0031034 | BP | myosin filament assembly | 1/284 | 2/12055 | 0.046564275 | 1 |
| 1 | GO:0031033 | BP | myosin filament organization | 1/284 | 2/12055 | 0.046564275 | 1 |
| 1 | GO:2000465 | BP | regulation of glycogen (starch) synthase activity | 1/284 | 2/12055 | 0.046564275 | 1 |
| 1 | GO:0030241 | BP | skeletal muscle myosin thick filament assembly | 1/284 | 2/12055 | 0.046564275 | 1 |
| 1 | GO:0043503 | BP | skeletal muscle fiber adaptation | 1/284 | 2/12055 | 0.046564275 | 1 |
| 1 | GO:2000696 | BP | regulation of epithelial cell differentiation involved in kidney development | 1/284 | 2/12055 | 0.046564275 | 1 |
| 1 | GO:0097065 | BP | anterior head development | 1/284 | 2/12055 | 0.046564275 | 1 |
| 1 | GO:0035313 | BP | wound healing, spreading of epidermal cells | 1/284 | 2/12055 | 0.046564275 | 1 |
| 1 | GO:0045161 | BP | neuronal ion channel clustering | 1/284 | 2/12055 | 0.046564275 | 1 |
| 1 | GO:0051547 | BP | regulation of keratinocyte migration | 1/284 | 2/12055 | 0.046564275 | 1 |
| 1 | GO:0051549 | BP | positive regulation of keratinocyte migration | 1/284 | 2/12055 | 0.046564275 | 1 |
| 1 | GO:0010463 | BP | mesenchymal cell proliferation | 1/284 | 2/12055 | 0.046564275 | 1 |
| 1 | GO:1901894 | BP | regulation of ATPase-coupled calcium transmembrane transporter activity | 1/284 | 2/12055 | 0.046564275 | 1 |
| 1 | GO:0019800 | BP | peptide cross-linking via chondroitin 4-sulfate glycosaminoglycan | 1/284 | 2/12055 | 0.046564275 | 1 |
| 1 | GO:0035358 | BP | regulation of peroxisome proliferator activated receptor signaling pathway | 1/284 | 2/12055 | 0.046564275 | 1 |
| 1 | GO:0006152 | BP | purine nucleoside catabolic process | 1/284 | 2/12055 | 0.046564275 | 1 |
| 1 | GO:0006154 | BP | adenosine catabolic process | 1/284 | 2/12055 | 0.046564275 | 1 |
| 1 | GO:0042535 | BP | positive regulation of tumor necrosis factor biosynthetic process | 1/284 | 2/12055 | 0.046564275 | 1 |
| 1 | GO:0032344 | BP | regulation of aldosterone metabolic process | 1/284 | 2/12055 | 0.046564275 | 1 |
| 1 | GO:0042454 | BP | ribonucleoside catabolic process | 1/284 | 2/12055 | 0.046564275 | 1 |
| 1 | GO:0007635 | BP | chemosensory behavior | 1/284 | 2/12055 | 0.046564275 | 1 |
| 1 | GO:0060134 | BP | prepulse inhibition | 1/284 | 2/12055 | 0.046564275 | 1 |
| 1 | GO:0009111 | BP | vitamin catabolic process | 1/284 | 2/12055 | 0.046564275 | 1 |
| 1 | GO:0014733 | BP | regulation of skeletal muscle adaptation | 1/284 | 2/12055 | 0.046564275 | 1 |
| 1 | GO:0060484 | BP | lung-associated mesenchyme development | 1/284 | 2/12055 | 0.046564275 | 1 |
| 1 | GO:0007252 | BP | I-kappaB phosphorylation | 1/284 | 2/12055 | 0.046564275 | 1 |
| 1 | GO:0048505 | BP | regulation of timing of cell differentiation | 1/284 | 2/12055 | 0.046564275 | 1 |
| 1 | GO:0031946 | BP | regulation of glucocorticoid biosynthetic process | 1/284 | 2/12055 | 0.046564275 | 1 |
| 1 | GO:0051901 | BP | positive regulation of mitochondrial depolarization | 1/284 | 2/12055 | 0.046564275 | 1 |
| 1 | GO:0051900 | BP | regulation of mitochondrial depolarization | 1/284 | 2/12055 | 0.046564275 | 1 |
| 1 | GO:0038084 | BP | vascular endothelial growth factor signaling pathway | 1/284 | 2/12055 | 0.046564275 | 1 |
| 1 | GO:0045080 | BP | positive regulation of chemokine biosynthetic process | 1/284 | 2/12055 | 0.046564275 | 1 |
| 1 | GO:0042048 | BP | olfactory behavior | 1/284 | 2/12055 | 0.046564275 | 1 |
| 1 | GO:0097205 | BP | renal filtration | 1/284 | 2/12055 | 0.046564275 | 1 |
| 1 | GO:0006546 | BP | glycine catabolic process | 1/284 | 2/12055 | 0.046564275 | 1 |
| 1 | GO:0048632 | BP | negative regulation of skeletal muscle tissue growth | 1/284 | 2/12055 | 0.046564275 | 1 |
| 1 | GO:0032353 | BP | negative regulation of hormone biosynthetic process | 1/284 | 2/12055 | 0.046564275 | 1 |
| 1 | GO:0032351 | BP | negative regulation of hormone metabolic process | 1/284 | 2/12055 | 0.046564275 | 1 |
| 1 | GO:1904181 | BP | positive regulation of membrane depolarization | 1/284 | 2/12055 | 0.046564275 | 1 |
| 1 | GO:0071688 | BP | striated muscle myosin thick filament assembly | 1/284 | 2/12055 | 0.046564275 | 1 |
| 1 | GO:0060445 | BP | branching involved in salivary gland morphogenesis | 1/284 | 2/12055 | 0.046564275 | 1 |
| 1 | GO:0006002 | BP | fructose 6-phosphate metabolic process | 1/284 | 2/12055 | 0.046564275 | 1 |
| 1 | GO:0040034 | BP | regulation of development, heterochronic | 1/284 | 2/12055 | 0.046564275 | 1 |
| 1 | GO:0038063 | BP | collagen-activated tyrosine kinase receptor signaling pathway | 1/284 | 2/12055 | 0.046564275 | 1 |
| 1 | GO:0038065 | BP | collagen-activated signaling pathway | 1/284 | 2/12055 | 0.046564275 | 1 |
| 1 | GO:0097527 | BP | necroptotic signaling pathway | 1/284 | 2/12055 | 0.046564275 | 1 |
| 1 | GO:0002634 | BP | regulation of germinal center formation | 1/284 | 2/12055 | 0.046564275 | 1 |
| 1 | GO:2000064 | BP | regulation of cortisol biosynthetic process | 1/284 | 2/12055 | 0.046564275 | 1 |
| 1 | GO:0046130 | BP | purine ribonucleoside catabolic process | 1/284 | 2/12055 | 0.046564275 | 1 |
| 1 | GO:0034127 | BP | regulation of MyD88-independent toll-like receptor signaling pathway | 1/284 | 2/12055 | 0.046564275 | 1 |
| 1 | GO:0060693 | BP | regulation of branching involved in salivary gland morphogenesis | 1/284 | 2/12055 | 0.046564275 | 1 |
| 1 | GO:0002730 | BP | regulation of dendritic cell cytokine production | 1/284 | 2/12055 | 0.046564275 | 1 |
| 1 | GO:1902548 | BP | negative regulation of cellular response to vascular endothelial growth factor stimulus | 1/284 | 2/12055 | 0.046564275 | 1 |
| 1 | GO:0035360 | BP | positive regulation of peroxisome proliferator activated receptor signaling pathway | 1/284 | 2/12055 | 0.046564275 | 1 |
| 1 | GO:0032908 | BP | regulation of transforming growth factor beta1 production | 1/284 | 2/12055 | 0.046564275 | 1 |
| 6 | GO:0044089 | BP | positive regulation of cellular component biogenesis | 6/284 | 111/12055 | 0.047225209 | 1 |
| 2 | GO:0051781 | BP | positive regulation of cell division | 2/284 | 15/12055 | 0.047455022 | 1 |
| 2 | GO:0006040 | BP | amino sugar metabolic process | 2/284 | 15/12055 | 0.047455022 | 1 |
| 2 | GO:0043266 | BP | regulation of potassium ion transport | 2/284 | 15/12055 | 0.047455022 | 1 |
| 2 | GO:0048660 | BP | regulation of smooth muscle cell proliferation | 2/284 | 15/12055 | 0.047455022 | 1 |
| 2 | GO:0010812 | BP | negative regulation of cell-substrate adhesion | 2/284 | 15/12055 | 0.047455022 | 1 |
| 4 | GO:0050678 | BP | regulation of epithelial cell proliferation | 4/284 | 58/12055 | 0.047471729 | 1 |
| 16 | GO:1902531 | BP | regulation of intracellular signal transduction | 16/284 | 418/12055 | 0.048813315 | 1 |
| 30 | GO:0065008 | BP | regulation of biological quality | 30/284 | 888/12055 | 0.0493466 | 1 |
| 2 | GO:0036454 | CC | growth factor complex | 2/284 | 2/12055 | 0.000553103 | 1 |
| 2 | GO:0042567 | CC | insulin-like growth factor ternary complex | 2/284 | 2/12055 | 0.000553103 | 1 |
| 2 | GO:0016942 | CC | insulin-like growth factor binding protein complex | 2/284 | 2/12055 | 0.000553103 | 1 |
| 2 | GO:0001527 | CC | microfibril | 2/284 | 3/12055 | 0.001633429 | 1 |
| 221 | GO:0005575 | CC | cellular_component | 221/284 | 8489/12055 | 0.005624977 | 1 |
| 3 | GO:0030175 | CC | filopodium | 3/284 | 18/12055 | 0.008121831 | 1 |
| 2 | GO:0098878 | CC | neurotransmitter receptor complex | 2/284 | 7/12055 | 0.010740416 | 1 |
| 2 | GO:0008328 | CC | ionotropic glutamate receptor complex | 2/284 | 7/12055 | 0.010740416 | 1 |
| 3 | GO:0045095 | CC | keratin filament | 3/284 | 20/12055 | 0.010960307 | 1 |
| 5 | GO:0044297 | CC | cell body | 5/284 | 62/12055 | 0.015196349 | 1 |
| 1 | GO:0071752 | CC | secretory dimeric IgA immunoglobulin complex | 1/284 | 1/12055 | 0.023558689 | 1 |
| 1 | GO:0071753 | CC | IgM immunoglobulin complex | 1/284 | 1/12055 | 0.023558689 | 1 |
| 1 | GO:0071750 | CC | dimeric IgA immunoglobulin complex | 1/284 | 1/12055 | 0.023558689 | 1 |
| 1 | GO:0071751 | CC | secretory IgA immunoglobulin complex | 1/284 | 1/12055 | 0.023558689 | 1 |
| 1 | GO:0071756 | CC | pentameric IgM immunoglobulin complex | 1/284 | 1/12055 | 0.023558689 | 1 |
| 1 | GO:0071754 | CC | IgM immunoglobulin complex, circulating | 1/284 | 1/12055 | 0.023558689 | 1 |
| 1 | GO:0035867 | CC | alphav-beta3 integrin-IGF-1-IGF1R complex | 1/284 | 1/12055 | 0.023558689 | 1 |
| 1 | GO:0038037 | CC | G protein-coupled receptor dimeric complex | 1/284 | 1/12055 | 0.023558689 | 1 |
| 1 | GO:0038039 | CC | G protein-coupled receptor heterodimeric complex | 1/284 | 1/12055 | 0.023558689 | 1 |
| 1 | GO:0005584 | CC | collagen type I trimer | 1/284 | 1/12055 | 0.023558689 | 1 |
| 1 | GO:0005586 | CC | collagen type III trimer | 1/284 | 1/12055 | 0.023558689 | 1 |
| 1 | GO:0005588 | CC | collagen type V trimer | 1/284 | 1/12055 | 0.023558689 | 1 |
| 1 | GO:0042571 | CC | immunoglobulin complex, circulating | 1/284 | 1/12055 | 0.023558689 | 1 |
| 1 | GO:0097648 | CC | G protein-coupled receptor complex | 1/284 | 1/12055 | 0.023558689 | 1 |
| 1 | GO:0071749 | CC | polymeric IgA immunoglobulin complex | 1/284 | 1/12055 | 0.023558689 | 1 |
| 1 | GO:0071748 | CC | monomeric IgA immunoglobulin complex | 1/284 | 1/12055 | 0.023558689 | 1 |
| 1 | GO:0071745 | CC | IgA immunoglobulin complex | 1/284 | 1/12055 | 0.023558689 | 1 |
| 1 | GO:0071746 | CC | IgA immunoglobulin complex, circulating | 1/284 | 1/12055 | 0.023558689 | 1 |
| 1 | GO:0030485 | CC | smooth muscle contractile fiber | 1/284 | 1/12055 | 0.023558689 | 1 |
| 1 | GO:0071953 | CC | elastic fiber | 1/284 | 1/12055 | 0.023558689 | 1 |
| 11 | GO:0099512 | CC | supramolecular fiber | 11/284 | 240/12055 | 0.030017051 | 1 |
| 11 | GO:0099080 | CC | supramolecular complex | 11/284 | 240/12055 | 0.030017051 | 1 |
| 11 | GO:0099081 | CC | supramolecular polymer | 11/284 | 240/12055 | 0.030017051 | 1 |
| 2 | GO:0030127 | CC | COPII vesicle coat | 2/284 | 12/12055 | 0.031237468 | 1 |
| 5 | GO:0034703 | CC | cation channel complex | 5/284 | 75/12055 | 0.031753489 | 1 |
| 3 | GO:0098858 | CC | actin-based cell projection | 3/284 | 30/12055 | 0.03286832 | 1 |
| 4 | GO:0044449 | CC | contractile fiber part | 4/284 | 53/12055 | 0.035846315 | 1 |
| 4 | GO:0000786 | CC | nucleosome | 4/284 | 57/12055 | 0.044998148 | 1 |
| 1 | GO:0070852 | CC | cell body fiber | 1/284 | 2/12055 | 0.046564275 | 1 |
| 1 | GO:0044214 | CC | spanning component of plasma membrane | 1/284 | 2/12055 | 0.046564275 | 1 |
| 1 | GO:0016460 | CC | myosin II complex | 1/284 | 2/12055 | 0.046564275 | 1 |
| 1 | GO:0005859 | CC | muscle myosin complex | 1/284 | 2/12055 | 0.046564275 | 1 |
| 1 | GO:0072562 | CC | blood microparticle | 1/284 | 2/12055 | 0.046564275 | 1 |
| 1 | GO:0089717 | CC | spanning component of membrane | 1/284 | 2/12055 | 0.046564275 | 1 |
| 9 | GO:0008083 | MF | growth factor activity | 9/284 | 78/12055 | 8.65315E-05 | 1 |
| 24 | GO:0005509 | MF | calcium ion binding | 24/284 | 436/12055 | 0.000125967 | 1 |
| 14 | GO:0048018 | MF | receptor ligand activity | 14/284 | 197/12055 | 0.000236203 | 1 |
| 14 | GO:0030546 | MF | signaling receptor activator activity | 14/284 | 198/12055 | 0.000248901 | 1 |
| 14 | GO:0030545 | MF | receptor regulator activity | 14/284 | 202/12055 | 0.000305741 | 1 |
| 25 | GO:0005102 | MF | signaling receptor binding | 25/284 | 504/12055 | 0.000425329 | 1 |
| 4 | GO:0030215 | MF | semaphorin receptor binding | 4/284 | 21/12055 | 0.001315142 | 1 |
| 6 | GO:1901681 | MF | sulfur compound binding | 6/284 | 53/12055 | 0.001469319 | 1 |
| 6 | GO:0005179 | MF | hormone activity | 6/284 | 57/12055 | 0.002147453 | 1 |
| 13 | GO:0046873 | MF | metal ion transmembrane transporter activity | 13/284 | 229/12055 | 0.003048936 | 1 |
| 2 | GO:0001968 | MF | fibronectin binding | 2/284 | 4/12055 | 0.003216 | 1 |
| 18 | GO:0005198 | MF | structural molecule activity | 18/284 | 374/12055 | 0.004509318 | 1 |
| 8 | GO:0030246 | MF | carbohydrate binding | 8/284 | 113/12055 | 0.005208802 | 1 |
| 2 | GO:0005159 | MF | insulin-like growth factor receptor binding | 2/284 | 5/12055 | 0.005276717 | 1 |
| 2 | GO:0043394 | MF | proteoglycan binding | 2/284 | 5/12055 | 0.005276717 | 1 |
| 2 | GO:0035252 | MF | UDP-xylosyltransferase activity | 2/284 | 5/12055 | 0.005276717 | 1 |
| 2 | GO:0042285 | MF | xylosyltransferase activity | 2/284 | 6/12055 | 0.007792331 | 1 |
| 2 | GO:0004096 | MF | catalase activity | 2/284 | 6/12055 | 0.007792331 | 1 |
| 2 | GO:0045125 | MF | bioactive lipid receptor activity | 2/284 | 7/12055 | 0.010740416 | 1 |
| 15 | GO:0022890 | MF | inorganic cation transmembrane transporter activity | 15/284 | 318/12055 | 0.012516238 | 1 |
| 8 | GO:0008237 | MF | metallopeptidase activity | 8/284 | 135/12055 | 0.01452768 | 1 |
| 4 | GO:0019838 | MF | growth factor binding | 4/284 | 42/12055 | 0.016723264 | 1 |
| 25 | GO:0098772 | MF | molecular function regulator | 25/284 | 658/12055 | 0.016815379 | 1 |
| 4 | GO:0005044 | MF | scavenger receptor activity | 4/284 | 43/12055 | 0.018105981 | 1 |
| 14 | GO:0044877 | MF | protein-containing complex binding | 14/284 | 305/12055 | 0.018796471 | 1 |
| 2 | GO:0042813 | MF | Wnt-activated receptor activity | 2/284 | 10/12055 | 0.021967056 | 1 |
| 2 | GO:0050840 | MF | extracellular matrix binding | 2/284 | 10/12055 | 0.021967056 | 1 |
| 2 | GO:0017147 | MF | Wnt-protein binding | 2/284 | 10/12055 | 0.021967056 | 1 |
| 2 | GO:0043531 | MF | ADP binding | 2/284 | 10/12055 | 0.021967056 | 1 |
| 1 | GO:0004996 | MF | thyroid-stimulating hormone receptor activity | 1/284 | 1/12055 | 0.023558689 | 1 |
| 1 | GO:0005415 | MF | nucleoside:sodium symporter activity | 1/284 | 1/12055 | 0.023558689 | 1 |
| 1 | GO:0003721 | MF | telomerase RNA reverse transcriptase activity | 1/284 | 1/12055 | 0.023558689 | 1 |
| 1 | GO:0004375 | MF | glycine dehydrogenase (decarboxylating) activity | 1/284 | 1/12055 | 0.023558689 | 1 |
| 1 | GO:0004971 | MF | AMPA glutamate receptor activity | 1/284 | 1/12055 | 0.023558689 | 1 |
| 1 | GO:0004720 | MF | protein-lysine 6-oxidase activity | 1/284 | 1/12055 | 0.023558689 | 1 |
| 1 | GO:0004064 | MF | arylesterase activity | 1/284 | 1/12055 | 0.023558689 | 1 |
| 1 | GO:0016167 | MF | glial cell-derived neurotrophic factor receptor activity | 1/284 | 1/12055 | 0.023558689 | 1 |
| 1 | GO:0019862 | MF | IgA binding | 1/284 | 1/12055 | 0.023558689 | 1 |
| 1 | GO:0030021 | MF | extracellular matrix structural constituent conferring compression resistance | 1/284 | 1/12055 | 0.023558689 | 1 |
| 1 | GO:0031994 | MF | insulin-like growth factor I binding | 1/284 | 1/12055 | 0.023558689 | 1 |
| 1 | GO:0019841 | MF | retinol binding | 1/284 | 1/12055 | 0.023558689 | 1 |
| 1 | GO:0070052 | MF | collagen V binding | 1/284 | 1/12055 | 0.023558689 | 1 |
| 1 | GO:0070051 | MF | fibrinogen binding | 1/284 | 1/12055 | 0.023558689 | 1 |
| 1 | GO:0019970 | MF | interleukin-11 binding | 1/284 | 1/12055 | 0.023558689 | 1 |
| 1 | GO:0043395 | MF | heparan sulfate proteoglycan binding | 1/284 | 1/12055 | 0.023558689 | 1 |
| 1 | GO:0042834 | MF | peptidoglycan binding | 1/284 | 1/12055 | 0.023558689 | 1 |
| 1 | GO:0070287 | MF | ferritin receptor activity | 1/284 | 1/12055 | 0.023558689 | 1 |
| 1 | GO:0004921 | MF | interleukin-11 receptor activity | 1/284 | 1/12055 | 0.023558689 | 1 |
| 1 | GO:0050501 | MF | hyaluronan synthase activity | 1/284 | 1/12055 | 0.023558689 | 1 |
| 1 | GO:0047390 | MF | glycerophosphocholine cholinephosphodiesterase activity | 1/284 | 1/12055 | 0.023558689 | 1 |
| 1 | GO:0008426 | MF | protein kinase C inhibitor activity | 1/284 | 1/12055 | 0.023558689 | 1 |
| 1 | GO:0032027 | MF | myosin light chain binding | 1/284 | 1/12055 | 0.023558689 | 1 |
| 1 | GO:0004489 | MF | methylenetetrahydrofolate reductase (NAD(P)H) activity | 1/284 | 1/12055 | 0.023558689 | 1 |
| 1 | GO:0016642 | MF | oxidoreductase activity, acting on the CH-NH2 group of donors, disulfide as acceptor | 1/284 | 1/12055 | 0.023558689 | 1 |
| 2 | GO:0004364 | MF | glutathione transferase activity | 2/284 | 11/12055 | 0.026436352 | 1 |
| 2 | GO:0015095 | MF | magnesium ion transmembrane transporter activity | 2/284 | 11/12055 | 0.026436352 | 1 |
| 2 | GO:0005160 | MF | transforming growth factor beta receptor binding | 2/284 | 11/12055 | 0.026436352 | 1 |
| 15 | GO:0008324 | MF | cation transmembrane transporter activity | 15/284 | 345/12055 | 0.027700733 | 1 |
| 4 | GO:0038024 | MF | cargo receptor activity | 4/284 | 49/12055 | 0.027879546 | 1 |
| 2 | GO:0015459 | MF | potassium channel regulator activity | 2/284 | 12/12055 | 0.031237468 | 1 |
| 2 | GO:0005158 | MF | insulin receptor binding | 2/284 | 12/12055 | 0.031237468 | 1 |
| 2 | GO:0004602 | MF | glutathione peroxidase activity | 2/284 | 12/12055 | 0.031237468 | 1 |
| 4 | GO:0015085 | MF | calcium ion transmembrane transporter activity | 4/284 | 51/12055 | 0.031715518 | 1 |
| 6 | GO:0015079 | MF | potassium ion transmembrane transporter activity | 6/284 | 101/12055 | 0.03199088 | 1 |
| 4 | GO:0015293 | MF | symporter activity | 4/284 | 53/12055 | 0.035846315 | 1 |
| 2 | GO:0016638 | MF | oxidoreductase activity, acting on the CH-NH2 group of donors | 2/284 | 13/12055 | 0.036352406 | 1 |
| 9 | GO:0005261 | MF | cation channel activity | 9/284 | 177/12055 | 0.038576972 | 1 |
| 5 | GO:0061134 | MF | peptidase regulator activity | 5/284 | 80/12055 | 0.040289115 | 1 |
| 2 | GO:0030291 | MF | protein serine/threonine kinase inhibitor activity | 2/284 | 14/12055 | 0.041763825 | 1 |
| 1 | GO:0004800 | MF | thyroxine 5'-deiodinase activity | 1/284 | 2/12055 | 0.046564275 | 1 |
| 1 | GO:0004937 | MF | alpha1-adrenergic receptor activity | 1/284 | 2/12055 | 0.046564275 | 1 |
| 1 | GO:0070700 | MF | BMP receptor binding | 1/284 | 2/12055 | 0.046564275 | 1 |
| 1 | GO:0034987 | MF | immunoglobulin receptor binding | 1/284 | 2/12055 | 0.046564275 | 1 |
| 1 | GO:0005250 | MF | A-type (transient outward) potassium channel activity | 1/284 | 2/12055 | 0.046564275 | 1 |
| 1 | GO:0016717 | MF | oxidoreductase activity, acting on paired donors, with oxidation of a pair of donors resulting in the reduction of molecular oxygen to two molecules of water | 1/284 | 2/12055 | 0.046564275 | 1 |
| 1 | GO:0004104 | MF | cholinesterase activity | 1/284 | 2/12055 | 0.046564275 | 1 |
| 1 | GO:0031685 | MF | adenosine receptor binding | 1/284 | 2/12055 | 0.046564275 | 1 |
| 1 | GO:0001972 | MF | retinoic acid binding | 1/284 | 2/12055 | 0.046564275 | 1 |
| 1 | GO:0030158 | MF | protein xylosyltransferase activity | 1/284 | 2/12055 | 0.046564275 | 1 |
| 1 | GO:0004965 | MF | G protein-coupled GABA receptor activity | 1/284 | 2/12055 | 0.046564275 | 1 |
| 1 | GO:0070696 | MF | transmembrane receptor protein serine/threonine kinase binding | 1/284 | 2/12055 | 0.046564275 | 1 |
| 1 | GO:0004949 | MF | cannabinoid receptor activity | 1/284 | 2/12055 | 0.046564275 | 1 |
| 1 | GO:0035727 | MF | lysophosphatidic acid binding | 1/284 | 2/12055 | 0.046564275 | 1 |
| 1 | GO:0000146 | MF | microfilament motor activity | 1/284 | 2/12055 | 0.046564275 | 1 |
| 1 | GO:0045159 | MF | myosin II binding | 1/284 | 2/12055 | 0.046564275 | 1 |
| 1 | GO:0004360 | MF | glutamine-fructose-6-phosphate transaminase (isomerizing) activity | 1/284 | 2/12055 | 0.046564275 | 1 |
| 2 | GO:0008484 | MF | sulfuric ester hydrolase activity | 2/284 | 15/12055 | 0.047455022 | 1 |
| 3 | GO:0004867 | MF | serine-type endopeptidase inhibitor activity | 3/284 | 35/12055 | 0.048661797 | 1 |
| 3 | GO:0005262 | MF | calcium channel activity | 3/284 | 35/12055 | 0.048661797 | 1 |
| 6 | GO:0004857 | MF | enzyme inhibitor activity | 6/284 | 112/12055 | 0.048958104 | 1 |

Note: DTG/DAG: represents the number of DEGS enriched in this GO term/ the number of DEGS enrich in all GO terms. BTG/BTG represents the number of all background genes enriched in this GO term/ the number of all background genes enriched in all GO term

| Table S5. KEGG pathways | | | | | | | | | | |
| --- | --- | --- | --- | --- | --- | --- | --- | --- | --- | --- |
| Num | Pathway id | Description | Database | DTK/DAK | BTK/BAK | Pvalue | Padjust | First Category | Second Category |  |
| 13 | map04974 | Protein digestion and absorption | KEGG PATHWAY | 13/260 | 93/11255 | 1.98034E-07 | 4.15871E-05 | Organismal Systems | Digestive system |  |
| 23 | map04151 | PI3K-Akt signaling pathway | KEGG PATHWAY | 23/260 | 341/11255 | 4.06484E-06 | 0.000426808 | Environmental Information Processing | Signal transduction |  |
| 10 | map05414 | Dilated cardiomyopathy (DCM) | KEGG PATHWAY | 10/260 | 94/11255 | 5.97889E-05 | 0.00418522 | Human Diseases | Cardiovascular diseases |  |
| 7 | map04672 | Intestinal immune network for IgA production | KEGG PATHWAY | 7/260 | 47/11255 | 9.24391E-05 | 0.004853052 | Organismal Systems | Immune system |  |
| 9 | map05322 | Systemic lupus erythematosus | KEGG PATHWAY | 9/260 | 91/11255 | 0.000244985 | 0.006430858 | Human Diseases | Immune diseases |  |
| 5 | map05310 | Asthma | KEGG PATHWAY | 5/260 | 25/11255 | 0.000230252 | 0.006907564 | Human Diseases | Immune diseases |  |
| 9 | map04512 | ECM-receptor interaction | KEGG PATHWAY | 9/260 | 94/11255 | 0.000313095 | 0.007305548 | Environmental Information Processing | Signaling molecules and interaction |  |
| 6 | map05340 | Primary immunodeficiency | KEGG PATHWAY | 6/260 | 38/11255 | 0.000212819 | 0.007448656 | Human Diseases | Immune diseases |  |
| 10 | map05146 | Amoebiasis | KEGG PATHWAY | 10/260 | 108/11255 | 0.000193001 | 0.008106031 | Human Diseases | Infectious diseases: Parasitic |  |
| 6 | map05320 | Autoimmune thyroid disease | KEGG PATHWAY | 6/260 | 43/11255 | 0.000426736 | 0.008961464 | Human Diseases | Immune diseases |  |
| 13 | map04020 | Calcium signaling pathway | KEGG PATHWAY | 13/260 | 193/11255 | 0.000538736 | 0.010284968 | Environmental Information Processing | Signal transduction |  |
| 14 | map04510 | Focal adhesion | KEGG PATHWAY | 14/260 | 221/11255 | 0.000614634 | 0.010756095 | Cellular Processes | Cellular community - eukaryotes |  |
| 8 | map05323 | Rheumatoid arthritis | KEGG PATHWAY | 8/260 | 87/11255 | 0.000880547 | 0.014224219 | Human Diseases | Immune diseases |  |
| 5 | map05143 | African trypanosomiasis | KEGG PATHWAY | 5/260 | 34/11255 | 0.001017837 | 0.015267562 | Human Diseases | Infectious diseases: Parasitic |  |
| 8 | map04640 | Hematopoietic cell lineage | KEGG PATHWAY | 8/260 | 91/11255 | 0.001183327 | 0.016566573 | Organismal Systems | Immune system |  |
| 5 | map05330 | Allograft rejection | KEGG PATHWAY | 5/260 | 37/11255 | 0.001507146 | 0.019781288 | Human Diseases | Immune diseases |  |
| 9 | map04926 | Relaxin signaling pathway | KEGG PATHWAY | 9/260 | 118/11255 | 0.001629832 | 0.020133219 | Organismal Systems | Endocrine system |  |
| 8 | map04666 | Fc gamma R-mediated phagocytosis | KEGG PATHWAY | 8/260 | 99/11255 | 0.002035615 | 0.023748842 | Organismal Systems | Immune system |  |
| 10 | map04145 | Phagosome | KEGG PATHWAY | 10/260 | 156/11255 | 0.003329248 | 0.036796954 | Cellular Processes | Transport and catabolism |  |
| 11 | map04360 | Axon guidance | KEGG PATHWAY | 11/260 | 184/11255 | 0.003630107 | 0.038116125 | Organismal Systems | Development |  |
| 5 | map00480 | Glutathione metabolism | KEGG PATHWAY | 5/260 | 48/11255 | 0.004819641 | 0.046005665 | Metabolism | Metabolism of other amino acids |  |
| 6 | map05140 | Leishmaniasis | KEGG PATHWAY | 6/260 | 68/11255 | 0.004743222 | 0.047432221 | Human Diseases | Infectious diseases: Parasitic |  |
| 6 | map05416 | Viral myocarditis | KEGG PATHWAY | 6/260 | 72/11255 | 0.006273798 | 0.054895735 | Human Diseases | Cardiovascular diseases |  |
| 5 | map05150 | Staphylococcus aureus infection | KEGG PATHWAY | 5/260 | 51/11255 | 0.00625264 | 0.057089325 | Human Diseases | Infectious diseases: Bacterial |  |
| 4 | map00980 | Metabolism of xenobiotics by cytochrome P450 | KEGG PATHWAY | 4/260 | 37/11255 | 0.010100754 | 0.075755657 | Metabolism | Xenobiotics biodegradation and metabolism |  |
| 6 | map04925 | Aldosterone synthesis and secretion | KEGG PATHWAY | 6/260 | 79/11255 | 0.009771208 | 0.075998281 | Organismal Systems | Endocrine system |  |
| 9 | map04072 | Phospholipase D signaling pathway | KEGG PATHWAY | 9/260 | 157/11255 | 0.010640944 | 0.077055109 | Environmental Information Processing | Signal transduction |  |
| 11 | map04060 | Cytokine-cytokine receptor interaction | KEGG PATHWAY | 11/260 | 210/11255 | 0.009581131 | 0.077386058 | Environmental Information Processing | Signaling molecules and interaction |  |
| 15 | map05165 | Human papillomavirus infection | KEGG PATHWAY | 15/260 | 328/11255 | 0.009228495 | 0.077519357 | Human Diseases | Infectious diseases: Viral |  |
| 5 | map04918 | Thyroid hormone synthesis | KEGG PATHWAY | 5/260 | 60/11255 | 0.012301438 | 0.086110068 | Organismal Systems | Endocrine system |  |
| 6 | map04350 | TGF-beta signaling pathway | KEGG PATHWAY | 6/260 | 86/11255 | 0.014475062 | 0.098056875 | Environmental Information Processing | Signal transduction |  |
| 7 | map04270 | Vascular smooth muscle contraction | KEGG PATHWAY | 7/260 | 112/11255 | 0.01503351 | 0.098657409 | Organismal Systems | Circulatory system |  |
| 10 | map04024 | cAMP signaling pathway | KEGG PATHWAY | 10/260 | 198/11255 | 0.016679113 | 0.106139811 | Environmental Information Processing | Signal transduction |  |
| 6 | map04650 | Natural killer cell mediated cytotoxicity | KEGG PATHWAY | 6/260 | 92/11255 | 0.019602697 | 0.121075483 | Organismal Systems | Immune system |  |
| 9 | map05202 | Transcriptional misregulation in cancer | KEGG PATHWAY | 9/260 | 179/11255 | 0.023142609 | 0.131349944 | Human Diseases | Cancers: Overview |  |
| 5 | map04664 | Fc epsilon RI signaling pathway | KEGG PATHWAY | 5/260 | 71/11255 | 0.023942574 | 0.132314225 | Organismal Systems | Immune system |  |
| 7 | map05034 | Alcoholism | KEGG PATHWAY | 7/260 | 122/11255 | 0.022944322 | 0.133841877 | Human Diseases | Substance dependence |  |
| 5 | map05218 | Melanoma | KEGG PATHWAY | 5/260 | 70/11255 | 0.022669262 | 0.136015572 | Human Diseases | Cancers: Specific types |  |
| 8 | map05225 | Hepatocellular carcinoma | KEGG PATHWAY | 8/260 | 154/11255 | 0.026465544 | 0.142506776 | Human Diseases | Cancers: Specific types |  |
| 4 | map05204 | Chemical carcinogenesis | KEGG PATHWAY | 4/260 | 50/11255 | 0.027939082 | 0.143102613 | Human Diseases | Cancers: Overview |  |
| 10 | map04810 | Regulation of actin cytoskeleton | KEGG PATHWAY | 10/260 | 215/11255 | 0.027669684 | 0.145265844 | Cellular Processes | Cell motility |  |
| 8 | map04022 | cGMP-PKG signaling pathway | KEGG PATHWAY | 8/260 | 158/11255 | 0.030214911 | 0.151074554 | Environmental Information Processing | Signal transduction |  |
| 6 | map04064 | NF-kappa B signaling pathway | KEGG PATHWAY | 6/260 | 104/11255 | 0.033293152 | 0.158899136 | Environmental Information Processing | Signal transduction |  |
| 5 | map04970 | Salivary secretion | KEGG PATHWAY | 5/260 | 77/11255 | 0.032548383 | 0.158957219 | Organismal Systems | Digestive system |  |
| 10 | map05205 | Proteoglycans in cancer | KEGG PATHWAY | 10/260 | 226/11255 | 0.037082819 | 0.173053157 | Human Diseases | Cancers: Overview |  |
| 2 | map00604 | Glycosphingolipid biosynthesis - ganglio series | KEGG PATHWAY | 2/260 | 14/11255 | 0.040293316 | 0.180033965 | Metabolism | Glycan biosynthesis and metabolism |  |
| 4 | map05144 | Malaria | KEGG PATHWAY | 4/260 | 56/11255 | 0.040076299 | 0.182957019 | Human Diseases | Infectious diseases: Parasitic |  |
| 18 | map05200 | Pathways in cancer | KEGG PATHWAY | 18/260 | 503/11255 | 0.043646016 | 0.187054354 | Human Diseases | Cancers: Overview |  |
| 5 | map04662 | B cell receptor signaling pathway | KEGG PATHWAY | 5/260 | 83/11255 | 0.042867574 | 0.187545635 | Organismal Systems | Immune system |  |
| 4 | map05031 | Amphetamine addiction | KEGG PATHWAY | 4/260 | 58/11255 | 0.044692076 | 0.187706717 | Human Diseases | Substance dependence |  |
| 3 | map00982 | Drug metabolism - cytochrome P450 | KEGG PATHWAY | 3/260 | 37/11255 | 0.053224186 | 0.219158413 | Metabolism | Xenobiotics biodegradation and metabolism |  |
| 9 | map04015 | Rap1 signaling pathway | KEGG PATHWAY | 9/260 | 212/11255 | 0.057602407 | 0.232625103 | Environmental Information Processing | Signal transduction |  |
| 7 | map05226 | Gastric cancer | KEGG PATHWAY | 7/260 | 150/11255 | 0.059143746 | 0.234343144 | Human Diseases | Cancers: Specific types |  |
| 6 | map05162 | Measles | KEGG PATHWAY | 6/260 | 121/11255 | 0.061426647 | 0.234538106 | Human Diseases | Infectious diseases: Viral |  |
| 7 | map04934 | Cushing syndrome | KEGG PATHWAY | 7/260 | 151/11255 | 0.060858173 | 0.236670674 | Human Diseases | Endocrine and metabolic diseases |  |
| 2 | map00532 | Glycosaminoglycan biosynthesis - chondroitin sulfate / dermatan sulfate | KEGG PATHWAY | 2/260 | 19/11255 | 0.070249758 | 0.263436594 | Metabolism | Glycan biosynthesis and metabolism |  |
| 7 | map05152 | Tuberculosis | KEGG PATHWAY | 7/260 | 158/11255 | 0.073708517 | 0.271557693 | Human Diseases | Infectious diseases: Bacterial |  |
| 5 | map05215 | Prostate cancer | KEGG PATHWAY | 5/260 | 98/11255 | 0.076404598 | 0.276637337 | Human Diseases | Cancers: Specific types |  |
| 5 | map04916 | Melanogenesis | KEGG PATHWAY | 5/260 | 101/11255 | 0.08443174 | 0.300519753 | Organismal Systems | Endocrine system |  |
| 5 | map04933 | AGE-RAGE signaling pathway in diabetic complications | KEGG PATHWAY | 5/260 | 103/11255 | 0.090021021 | 0.315073572 | Human Diseases | Endocrine and metabolic diseases |  |
| 3 | map04913 | Ovarian steroidogenesis | KEGG PATHWAY | 3/260 | 48/11255 | 0.098840256 | 0.340269734 | Organismal Systems | Endocrine system |  |
| 6 | map04310 | Wnt signaling pathway | KEGG PATHWAY | 6/260 | 139/11255 | 0.102974343 | 0.348784064 | Environmental Information Processing | Signal transduction |  |
| 3 | map00640 | Propanoate metabolism | KEGG PATHWAY | 3/260 | 50/11255 | 0.108408184 | 0.361360614 | Metabolism | Carbohydrate metabolism |  |
| 3 | map04923 | Regulation of lipolysis in adipocytes | KEGG PATHWAY | 3/260 | 53/11255 | 0.123397249 | 0.398668037 | Organismal Systems | Endocrine system |  |
| 10 | map04010 | MAPK signaling pathway | KEGG PATHWAY | 10/260 | 284/11255 | 0.122606319 | 0.402301983 | Environmental Information Processing | Signal transduction |  |
| 6 | map05224 | Breast cancer | KEGG PATHWAY | 6/260 | 149/11255 | 0.131101099 | 0.41713986 | Human Diseases | Cancers: Specific types |  |
| 4 | map05410 | Hypertrophic cardiomyopathy (HCM) | KEGG PATHWAY | 4/260 | 86/11255 | 0.137622684 | 0.431354681 | Human Diseases | Cardiovascular diseases |  |
| 3 | map04927 | Cortisol synthesis and secretion | KEGG PATHWAY | 3/260 | 58/11255 | 0.149905377 | 0.462943076 | Organismal Systems | Endocrine system |  |
| 3 | map00590 | Arachidonic acid metabolism | KEGG PATHWAY | 3/260 | 58/11255 | 0.149905377 | 0.462943076 | Metabolism | Lipid metabolism |  |
| 4 | map04211 | Longevity regulating pathway | KEGG PATHWAY | 4/260 | 90/11255 | 0.154670212 | 0.464010637 | Organismal Systems | Aging |  |
| 2 | map00512 | Mucin type O-glycan biosynthesis | KEGG PATHWAY | 2/260 | 31/11255 | 0.160090061 | 0.466929345 | Metabolism | Glycan biosynthesis and metabolism |  |
| 4 | map04610 | Complement and coagulation cascades | KEGG PATHWAY | 4/260 | 92/11255 | 0.16347857 | 0.470280818 | Organismal Systems | Immune system |  |
| 4 | map04540 | Gap junction | KEGG PATHWAY | 4/260 | 91/11255 | 0.159051655 | 0.470434473 | Cellular Processes | Cellular community - eukaryotes |  |
| 2 | map01040 | Biosynthesis of unsaturated fatty acids | KEGG PATHWAY | 2/260 | 32/11255 | 0.168301045 | 0.477611073 | Metabolism | Lipid metabolism |  |
| 1 | map00430 | Taurine and hypotaurine metabolism | KEGG PATHWAY | 1/260 | 8/11255 | 0.170584181 | 0.477635706 | Metabolism | Metabolism of other amino acids |  |
| 5 | map04152 | AMPK signaling pathway | KEGG PATHWAY | 5/260 | 129/11255 | 0.178368994 | 0.492861694 | Environmental Information Processing | Signal transduction |  |
| 6 | map04390 | Hippo signaling pathway | KEGG PATHWAY | 6/260 | 166/11255 | 0.186221969 | 0.507878099 | Environmental Information Processing | Signal transduction |  |
| 3 | map05217 | Basal cell carcinoma | KEGG PATHWAY | 3/260 | 66/11255 | 0.195564094 | 0.526518715 | Human Diseases | Cancers: Specific types |  |
| 2 | map00250 | Alanine, aspartate and glutamate metabolism | KEGG PATHWAY | 2/260 | 36/11255 | 0.201770991 | 0.536353268 | Metabolism | Amino acid metabolism |  |
| 4 | map05231 | Choline metabolism in cancer | KEGG PATHWAY | 4/260 | 104/11255 | 0.21970723 | 0.555885764 | Human Diseases | Cancers: Overview |  |
| 3 | map04911 | Insulin secretion | KEGG PATHWAY | 3/260 | 70/11255 | 0.219543894 | 0.562246559 | Organismal Systems | Endocrine system |  |
| 5 | map04514 | Cell adhesion molecules (CAMs) | KEGG PATHWAY | 5/260 | 138/11255 | 0.214609483 | 0.563349893 | Environmental Information Processing | Signaling molecules and interaction |  |
| 5 | map04550 | Signaling pathways regulating pluripotency of stem cells | KEGG PATHWAY | 5/260 | 139/11255 | 0.218774973 | 0.567194376 | Cellular Processes | Cellular community - eukaryotes |  |
| 4 | map03320 | PPAR signaling pathway | KEGG PATHWAY | 4/260 | 106/11255 | 0.229550206 | 0.573875514 | Organismal Systems | Endocrine system |  |
| 2 | map00140 | Steroid hormone biosynthesis | KEGG PATHWAY | 2/260 | 41/11255 | 0.244504428 | 0.604069764 | Metabolism | Lipid metabolism |  |
| 1 | map00603 | Glycosphingolipid biosynthesis - globo and isoglobo series | KEGG PATHWAY | 1/260 | 13/11255 | 0.262137701 | 0.632746174 | Metabolism | Glycan biosynthesis and metabolism |  |
| 2 | map05030 | Cocaine addiction | KEGG PATHWAY | 2/260 | 43/11255 | 0.261722366 | 0.639089498 | Human Diseases | Substance dependence |  |
| 2 | map05219 | Bladder cancer | KEGG PATHWAY | 2/260 | 44/11255 | 0.270337021 | 0.645122437 | Human Diseases | Cancers: Specific types |  |
| 1 | map00730 | Thiamine metabolism | KEGG PATHWAY | 1/260 | 14/11255 | 0.279202653 | 0.658792777 | Metabolism | Metabolism of cofactors and vitamins |  |
| 3 | map04914 | Progesterone-mediated oocyte maturation | KEGG PATHWAY | 3/260 | 81/11255 | 0.28793022 | 0.67183718 | Organismal Systems | Endocrine system |  |
| 9 | map04080 | Neuroactive ligand-receptor interaction | KEGG PATHWAY | 9/260 | 311/11255 | 0.292353208 | 0.674661248 | Environmental Information Processing | Signaling molecules and interaction |  |
| 3 | map01521 | EGFR tyrosine kinase inhibitor resistance | KEGG PATHWAY | 3/260 | 83/11255 | 0.300587446 | 0.686123519 | Human Diseases | Drug resistance: Antineoplastic |  |
| 4 | map04261 | Adrenergic signaling in cardiomyocytes | KEGG PATHWAY | 4/260 | 123/11255 | 0.31663224 | 0.6999239 | Organismal Systems | Circulatory system |  |
| 2 | map05033 | Nicotine addiction | KEGG PATHWAY | 2/260 | 50/11255 | 0.321822849 | 0.703987482 | Human Diseases | Substance dependence |  |
| 7 | map04014 | Ras signaling pathway | KEGG PATHWAY | 7/260 | 239/11255 | 0.315521239 | 0.704887875 | Environmental Information Processing | Signal transduction |  |
| 3 | map04713 | Circadian entrainment | KEGG PATHWAY | 3/260 | 88/11255 | 0.332322538 | 0.704926595 | Organismal Systems | Environmental adaptation |  |
| 3 | map01522 | Endocrine resistance | KEGG PATHWAY | 3/260 | 85/11255 | 0.31327307 | 0.707390803 | Human Diseases | Drug resistance: Antineoplastic |  |
| 2 | map04962 | Vasopressin-regulated water reabsorption | KEGG PATHWAY | 2/260 | 51/11255 | 0.330333081 | 0.707856603 | Organismal Systems | Excretory system |  |
| 6 | map05166 | Human T-cell leukemia virus 1 infection | KEGG PATHWAY | 6/260 | 203/11255 | 0.328549031 | 0.711291717 | Human Diseases | Infectious diseases: Viral |  |
| 1 | map00670 | One carbon pool by folate | KEGG PATHWAY | 1/260 | 18/11255 | 0.343619811 | 0.721601603 | Metabolism | Metabolism of cofactors and vitamins |  |
| 1 | map00360 | Phenylalanine metabolism | KEGG PATHWAY | 1/260 | 18/11255 | 0.343619811 | 0.721601603 | Metabolism | Amino acid metabolism |  |
| 4 | map04611 | Platelet activation | KEGG PATHWAY | 4/260 | 130/11255 | 0.353433994 | 0.727658224 | Organismal Systems | Immune system |  |
| 3 | map04972 | Pancreatic secretion | KEGG PATHWAY | 3/260 | 93/11255 | 0.364013013 | 0.742162454 | Organismal Systems | Digestive system |  |
| 4 | map04217 | Necroptosis | KEGG PATHWAY | 4/260 | 133/11255 | 0.369231498 | 0.745563602 | Cellular Processes | Cell growth and death |  |
| 2 | map05214 | Glioma | KEGG PATHWAY | 2/260 | 65/11255 | 0.445123475 | 0.772528346 | Human Diseases | Cancers: Specific types |  |
| 4 | map04150 | mTOR signaling pathway | KEGG PATHWAY | 4/260 | 145/11255 | 0.431962202 | 0.775316772 | Environmental Information Processing | Signal transduction |  |
| 1 | map04614 | Renin-angiotensin system | KEGG PATHWAY | 1/260 | 24/11255 | 0.429650023 | 0.777814696 | Organismal Systems | Endocrine system |  |
| 2 | map04213 | Longevity regulating pathway - multiple species | KEGG PATHWAY | 2/260 | 58/11255 | 0.388907353 | 0.777814706 | Organismal Systems | Aging |  |
| 2 | map01524 | Platinum drug resistance | KEGG PATHWAY | 2/260 | 64/11255 | 0.437263503 | 0.778180811 | Human Diseases | Drug resistance: Antineoplastic |  |
| 3 | map04725 | Cholinergic synapse | KEGG PATHWAY | 3/260 | 103/11255 | 0.426489122 | 0.778806224 | Organismal Systems | Nervous system |  |
| 1 | map00515 | Mannose type O-glycan biosynthesis | KEGG PATHWAY | 1/260 | 23/11255 | 0.41613462 | 0.780252413 | Metabolism | Glycan biosynthesis and metabolism |  |
| 1 | map04320 | Dorso-ventral axis formation | KEGG PATHWAY | 1/260 | 25/11255 | 0.442853744 | 0.781506607 | Organismal Systems | Development |  |
| 1 | map00534 | Glycosaminoglycan biosynthesis - heparan sulfate / heparin | KEGG PATHWAY | 1/260 | 25/11255 | 0.442853744 | 0.781506607 | Metabolism | Glycan biosynthesis and metabolism |  |
| 2 | map04730 | Long-term depression | KEGG PATHWAY | 2/260 | 62/11255 | 0.421365817 | 0.783069218 | Organismal Systems | Nervous system |  |
| 2 | map00010 | Glycolysis / Gluconeogenesis | KEGG PATHWAY | 2/260 | 62/11255 | 0.421365817 | 0.783069218 | Metabolism | Carbohydrate metabolism |  |
| 1 | map00514 | Other types of O-glycan biosynthesis | KEGG PATHWAY | 1/260 | 26/11255 | 0.455752945 | 0.784492774 | Metabolism | Glycan biosynthesis and metabolism |  |
| 3 | map04114 | Oocyte meiosis | KEGG PATHWAY | 3/260 | 100/11255 | 0.407930725 | 0.785921581 | Cellular Processes | Cell growth and death |  |
| 1 | map00340 | Histidine metabolism | KEGG PATHWAY | 1/260 | 27/11255 | 0.468354622 | 0.786835765 | Metabolism | Amino acid metabolism |  |
| 2 | map04721 | Synaptic vesicle cycle | KEGG PATHWAY | 2/260 | 61/11255 | 0.413331356 | 0.789087134 | Organismal Systems | Nervous system |  |
| 2 | map00983 | Drug metabolism - other enzymes | KEGG PATHWAY | 2/260 | 61/11255 | 0.413331356 | 0.789087134 | Metabolism | Xenobiotics biodegradation and metabolism |  |
| 4 | map04921 | Oxytocin signaling pathway | KEGG PATHWAY | 4/260 | 151/11255 | 0.46274285 | 0.790048768 | Organismal Systems | Endocrine system |  |
| 5 | map05203 | Viral carcinogenesis | KEGG PATHWAY | 5/260 | 181/11255 | 0.407182117 | 0.791743005 | Human Diseases | Cancers: Overview |  |
| 2 | map04115 | p53 signaling pathway | KEGG PATHWAY | 2/260 | 68/11255 | 0.468330556 | 0.793140458 | Cellular Processes | Cell growth and death |  |
| 2 | map04924 | Renin secretion | KEGG PATHWAY | 2/260 | 60/11255 | 0.405242167 | 0.795335094 | Organismal Systems | Endocrine system |  |
| 5 | map05169 | Epstein-Barr virus infection | KEGG PATHWAY | 5/260 | 200/11255 | 0.492911434 | 0.796241547 | Human Diseases | Infectious diseases: Viral |  |
| 1 | map00531 | Glycosaminoglycan degradation | KEGG PATHWAY | 1/260 | 22/11255 | 0.402300209 | 0.797009847 | Metabolism | Glycan biosynthesis and metabolism |  |
| 3 | map04915 | Estrogen signaling pathway | KEGG PATHWAY | 3/260 | 115/11255 | 0.498400514 | 0.798962655 | Organismal Systems | Endocrine system |  |
| 1 | map04745 | Phototransduction - fly | KEGG PATHWAY | 1/260 | 28/11255 | 0.480665612 | 0.801109354 | Organismal Systems | Sensory system |  |
| 1 | map00030 | Pentose phosphate pathway | KEGG PATHWAY | 1/260 | 28/11255 | 0.480665612 | 0.801109354 | Metabolism | Carbohydrate metabolism |  |
| 4 | map04062 | Chemokine signaling pathway | KEGG PATHWAY | 4/260 | 160/11255 | 0.507758313 | 0.801723652 | Organismal Systems | Immune system |  |
| 1 | map00592 | alpha-Linolenic acid metabolism | KEGG PATHWAY | 1/260 | 29/11255 | 0.492692596 | 0.802057714 | Metabolism | Lipid metabolism |  |
| 1 | map01523 | Antifolate resistance | KEGG PATHWAY | 1/260 | 30/11255 | 0.504442099 | 0.802521521 | Human Diseases | Drug resistance: Antineoplastic |  |
| 3 | map04724 | Glutamatergic synapse | KEGG PATHWAY | 3/260 | 114/11255 | 0.492573892 | 0.808129041 | Organismal Systems | Nervous system |  |
| 1 | map00051 | Fructose and mannose metabolism | KEGG PATHWAY | 1/260 | 34/11255 | 0.548790698 | 0.853674419 | Metabolism | Carbohydrate metabolism |  |
| 1 | map04960 | Aldosterone-regulated sodium reabsorption | KEGG PATHWAY | 1/260 | 34/11255 | 0.548790698 | 0.853674419 | Organismal Systems | Excretory system |  |
| 2 | map05412 | Arrhythmogenic right ventricular cardiomyopathy (ARVC) | KEGG PATHWAY | 2/260 | 79/11255 | 0.548228947 | 0.859164768 | Human Diseases | Cardiovascular diseases |  |
| 4 | map00230 | Purine metabolism | KEGG PATHWAY | 4/260 | 173/11255 | 0.56960691 | 0.860557202 | Metabolism | Nucleotide metabolism |  |
| 2 | map04727 | GABAergic synapse | KEGG PATHWAY | 2/260 | 83/11255 | 0.575132957 | 0.862699435 | Organismal Systems | Nervous system |  |
| 1 | map04978 | Mineral absorption | KEGG PATHWAY | 1/260 | 36/11255 | 0.569459157 | 0.872893598 | Organismal Systems | Digestive system |  |
| 1 | map00350 | Tyrosine metabolism | KEGG PATHWAY | 1/260 | 36/11255 | 0.569459157 | 0.872893598 | Metabolism | Amino acid metabolism |  |
| 1 | map00410 | beta-Alanine metabolism | KEGG PATHWAY | 1/260 | 39/11255 | 0.598706673 | 0.891690789 | Metabolism | Metabolism of other amino acids |  |
| 2 | map04380 | Osteoclast differentiation | KEGG PATHWAY | 2/260 | 104/11255 | 0.697062677 | 0.89805621 | Organismal Systems | Development |  |
| 4 | map04714 | Thermogenesis | KEGG PATHWAY | 4/260 | 205/11255 | 0.701511652 | 0.898277115 | Organismal Systems | Environmental adaptation |  |
| 1 | map00260 | Glycine, serine and threonine metabolism | KEGG PATHWAY | 1/260 | 40/11255 | 0.608009122 | 0.899168419 | Metabolism | Amino acid metabolism |  |
| 4 | map05163 | Human cytomegalovirus infection | KEGG PATHWAY | 4/260 | 203/11255 | 0.694192694 | 0.899879418 | Human Diseases | Infectious diseases: Viral |  |
| 1 | map00380 | Tryptophan metabolism | KEGG PATHWAY | 1/260 | 44/11255 | 0.643119465 | 0.900367251 | Metabolism | Amino acid metabolism |  |
| 1 | map00520 | Amino sugar and nucleotide sugar metabolism | KEGG PATHWAY | 1/260 | 45/11255 | 0.651396063 | 0.905915054 | Metabolism | Carbohydrate metabolism |  |
| 1 | map00565 | Ether lipid metabolism | KEGG PATHWAY | 1/260 | 47/11255 | 0.667379983 | 0.910063614 | Metabolism | Lipid metabolism |  |
| 1 | map05014 | Amyotrophic lateral sclerosis (ALS) | KEGG PATHWAY | 1/260 | 47/11255 | 0.667379983 | 0.910063614 | Human Diseases | Neurodegenerative diseases |  |
| 3 | map05206 | MicroRNAs in cancer | KEGG PATHWAY | 3/260 | 150/11255 | 0.677701068 | 0.9122899 | Human Diseases | Cancers: Overview |  |
| 2 | map04928 | Parathyroid hormone synthesis, secretion and action | KEGG PATHWAY | 2/260 | 94/11255 | 0.64301572 | 0.912387171 | Organismal Systems | Endocrine system |  |
| 2 | map04066 | HIF-1 signaling pathway | KEGG PATHWAY | 2/260 | 94/11255 | 0.64301572 | 0.912387171 | Environmental Information Processing | Signal transduction |  |
| 1 | map05110 | Vibrio cholerae infection | KEGG PATHWAY | 1/260 | 49/11255 | 0.682633711 | 0.913076938 | Human Diseases | Infectious diseases: Bacterial |  |
| 1 | map04975 | Fat digestion and absorption | KEGG PATHWAY | 1/260 | 49/11255 | 0.682633711 | 0.913076938 | Organismal Systems | Digestive system |  |
| 1 | map00330 | Arginine and proline metabolism | KEGG PATHWAY | 1/260 | 49/11255 | 0.682633711 | 0.913076938 | Metabolism | Amino acid metabolism |  |
| 1 | map00630 | Glyoxylate and dicarboxylate metabolism | KEGG PATHWAY | 1/260 | 49/11255 | 0.682633711 | 0.913076938 | Metabolism | Carbohydrate metabolism |  |
| 1 | map04612 | Antigen processing and presentation | KEGG PATHWAY | 1/260 | 49/11255 | 0.682633711 | 0.913076938 | Organismal Systems | Immune system |  |
| 2 | map04750 | Inflammatory mediator regulation of TRP channels | KEGG PATHWAY | 2/260 | 98/11255 | 0.665494303 | 0.913423553 | Organismal Systems | Sensory system |  |
| 3 | map04068 | FoxO signaling pathway | KEGG PATHWAY | 3/260 | 142/11255 | 0.64126731 | 0.916096157 | Environmental Information Processing | Signal transduction |  |
| 2 | map05032 | Morphine addiction | KEGG PATHWAY | 2/260 | 93/11255 | 0.63721385 | 0.916540469 | Human Diseases | Substance dependence |  |
| 3 | map05161 | Hepatitis B | KEGG PATHWAY | 3/260 | 147/11255 | 0.664366663 | 0.917874995 | Human Diseases | Infectious diseases: Viral |  |
| 3 | map05418 | Fluid shear stress and atherosclerosis | KEGG PATHWAY | 3/260 | 139/11255 | 0.62688449 | 0.920599601 | Human Diseases | Cardiovascular diseases |  |
| 3 | map04630 | Jak-STAT signaling pathway | KEGG PATHWAY | 3/260 | 140/11255 | 0.631722166 | 0.921261492 | Environmental Information Processing | Signal transduction |  |
| 3 | map04723 | Retrograde endocannabinoid signaling | KEGG PATHWAY | 3/260 | 140/11255 | 0.631722166 | 0.921261492 | Organismal Systems | Nervous system |  |
| 2 | map04728 | Dopaminergic synapse | KEGG PATHWAY | 2/260 | 111/11255 | 0.730743123 | 0.924434071 | Organismal Systems | Nervous system |  |
| 1 | map05230 | Central carbon metabolism in cancer | KEGG PATHWAY | 1/260 | 57/11255 | 0.736989182 | 0.926752864 | Human Diseases | Cancers: Overview |  |
| 1 | map04742 | Taste transduction | KEGG PATHWAY | 1/260 | 58/11255 | 0.743095881 | 0.928869851 | Organismal Systems | Sensory system |  |
| 1 | map05321 | Inflammatory bowel disease (IBD) | KEGG PATHWAY | 1/260 | 56/11255 | 0.730737897 | 0.93003005 | Human Diseases | Immune diseases |  |
| 1 | map04720 | Long-term potentiation | KEGG PATHWAY | 1/260 | 60/11255 | 0.754888768 | 0.938027463 | Organismal Systems | Nervous system |  |
| 2 | map04931 | Insulin resistance | KEGG PATHWAY | 2/260 | 121/11255 | 0.773318846 | 0.938710738 | Human Diseases | Endocrine and metabolic diseases |  |
| 2 | map04670 | Leukocyte transendothelial migration | KEGG PATHWAY | 2/260 | 120/11255 | 0.769340514 | 0.939311093 | Organismal Systems | Immune system |  |
| 1 | map04370 | VEGF signaling pathway | KEGG PATHWAY | 1/260 | 61/11255 | 0.760581391 | 0.939541719 | Environmental Information Processing | Signal transduction |  |
| 1 | map04212 | Longevity regulating pathway - worm | KEGG PATHWAY | 1/260 | 61/11255 | 0.760581391 | 0.939541719 | Organismal Systems | Aging |  |
| 2 | map04110 | Cell cycle | KEGG PATHWAY | 2/260 | 126/11255 | 0.792326054 | 0.940047861 | Cellular Processes | Cell growth and death |  |
| 2 | map04142 | Lysosome | KEGG PATHWAY | 2/260 | 125/11255 | 0.788640435 | 0.940991428 | Cellular Processes | Transport and catabolism |  |
| 1 | map04971 | Gastric acid secretion | KEGG PATHWAY | 1/260 | 65/11255 | 0.782064773 | 0.943871278 | Organismal Systems | Digestive system |  |
| 1 | map04976 | Bile secretion | KEGG PATHWAY | 1/260 | 65/11255 | 0.782064773 | 0.943871278 | Organismal Systems | Digestive system |  |
| 3 | map05016 | Huntington disease | KEGG PATHWAY | 3/260 | 184/11255 | 0.802105772 | 0.946304563 | Human Diseases | Neurodegenerative diseases |  |
| 1 | map05212 | Pancreatic cancer | KEGG PATHWAY | 1/260 | 73/11255 | 0.819439738 | 0.961353883 | Human Diseases | Cancers: Specific types |  |
| 1 | map05211 | Renal cell carcinoma | KEGG PATHWAY | 1/260 | 75/11255 | 0.827739137 | 0.965695659 | Human Diseases | Cancers: Specific types |  |
| 1 | map05012 | Parkinson disease | KEGG PATHWAY | 1/260 | 123/11255 | 0.944458142 | 0.967493706 | Human Diseases | Neurodegenerative diseases |  |
| 1 | map04919 | Thyroid hormone signaling pathway | KEGG PATHWAY | 1/260 | 122/11255 | 0.94313 | 0.970869118 | Organismal Systems | Endocrine system |  |
| 1 | map05160 | Hepatitis C | KEGG PATHWAY | 1/260 | 119/11255 | 0.938952751 | 0.976138999 | Human Diseases | Infectious diseases: Viral |  |
| 1 | map04140 | Autophagy - animal | KEGG PATHWAY | 1/260 | 119/11255 | 0.938952751 | 0.976138999 | Cellular Processes | Transport and catabolism |  |
| 1 | map04620 | Toll-like receptor signaling pathway | KEGG PATHWAY | 1/260 | 92/11255 | 0.884565693 | 0.977677871 | Organismal Systems | Immune system |  |
| 3 | map04530 | Tight junction | KEGG PATHWAY | 3/260 | 201/11255 | 0.847539647 | 0.977930362 | Cellular Processes | Cellular community - eukaryotes |  |
| 1 | map05220 | Chronic myeloid leukemia | KEGG PATHWAY | 1/260 | 79/11255 | 0.843215037 | 0.978315789 | Human Diseases | Cancers: Specific types |  |
| 1 | map04922 | Glucagon signaling pathway | KEGG PATHWAY | 1/260 | 94/11255 | 0.889880521 | 0.978402667 | Organismal Systems | Endocrine system |  |
| 1 | map05167 | Kaposi sarcoma-associated herpesvirus infection | KEGG PATHWAY | 1/260 | 165/11255 | 0.979453521 | 0.979453521 | Human Diseases | Infectious diseases: Viral |  |
| 1 | map04071 | Sphingolipid signaling pathway | KEGG PATHWAY | 1/260 | 115/11255 | 0.932903963 | 0.979549161 | Environmental Information Processing | Signal transduction |  |
| 1 | map04722 | Neurotrophin signaling pathway | KEGG PATHWAY | 1/260 | 115/11255 | 0.932903963 | 0.979549161 | Organismal Systems | Nervous system |  |
| 1 | map04910 | Insulin signaling pathway | KEGG PATHWAY | 1/260 | 138/11255 | 0.961044745 | 0.979705808 | Organismal Systems | Endocrine system |  |
| 1 | map05210 | Colorectal cancer | KEGG PATHWAY | 1/260 | 91/11255 | 0.881813224 | 0.979792471 | Human Diseases | Cancers: Specific types |  |
| 1 | map05142 | Chagas disease (American trypanosomiasis) | KEGG PATHWAY | 1/260 | 113/11255 | 0.929659303 | 0.981047506 | Human Diseases | Infectious diseases: Parasitic |  |
| 2 | map05164 | Influenza A | KEGG PATHWAY | 2/260 | 152/11255 | 0.870125825 | 0.982400125 | Human Diseases | Infectious diseases: Viral |  |
| 1 | map04210 | Apoptosis | KEGG PATHWAY | 1/260 | 147/11255 | 0.968520165 | 0.98255669 | Cellular Processes | Cell growth and death |  |
| 1 | map05168 | Herpes simplex infection | KEGG PATHWAY | 1/260 | 163/11255 | 0.978455284 | 0.983136888 | Human Diseases | Infectious diseases: Viral |  |
| 1 | map04740 | Olfactory transduction | KEGG PATHWAY | 1/260 | 155/11255 | 0.973955463 | 0.98332042 | Organismal Systems | Sensory system |  |
| 2 | map04141 | Protein processing in endoplasmic reticulum | KEGG PATHWAY | 2/260 | 157/11255 | 0.881618161 | 0.984786244 | Genetic Information Processing | Folding, sorting and degradation |  |
| 1 | map00561 | Glycerolipid metabolism | KEGG PATHWAY | 1/260 | 110/11255 | 0.924496971 | 0.985504385 | Metabolism | Lipid metabolism |  |
| 3 | map04144 | Endocytosis | KEGG PATHWAY | 3/260 | 248/11255 | 0.929250982 | 0.985569224 | Cellular Processes | Transport and catabolism |  |
| 1 | map04520 | Adherens junction | KEGG PATHWAY | 1/260 | 85/11255 | 0.863870163 | 0.985938773 | Cellular Processes | Cellular community - eukaryotes |  |
| 1 | map04912 | GnRH signaling pathway | KEGG PATHWAY | 1/260 | 85/11255 | 0.863870163 | 0.985938773 | Organismal Systems | Endocrine system |  |
| 1 | map04668 | TNF signaling pathway | KEGG PATHWAY | 1/260 | 101/11255 | 0.906634319 | 0.9864933 | Environmental Information Processing | Signal transduction |  |
| 1 | map04657 | IL-17 signaling pathway | KEGG PATHWAY | 1/260 | 90/11255 | 0.878995383 | 0.987107114 | Organismal Systems | Immune system |  |
| 1 | map03040 | Spliceosome | KEGG PATHWAY | 1/260 | 109/11255 | 0.922693665 | 0.988600355 | Genetic Information Processing | Transcription |  |
| 1 | map00564 | Glycerophospholipid metabolism | KEGG PATHWAY | 1/260 | 100/11255 | 0.904406225 | 0.989194309 | Metabolism | Lipid metabolism |  |
| 1 | map04660 | T cell receptor signaling pathway | KEGG PATHWAY | 1/260 | 107/11255 | 0.918957336 | 0.989646361 | Organismal Systems | Immune system |  |
| 2 | map04218 | Cellular senescence | KEGG PATHWAY | 2/260 | 149/11255 | 0.862748434 | 0.990039186 | Cellular Processes | Cell growth and death |  |
| 1 | map05145 | Toxoplasmosis | KEGG PATHWAY | 1/260 | 105/11255 | 0.915041152 | 0.990508463 | Human Diseases | Infectious diseases: Parasitic |  |

Note: DTK/DAK represents the number of DEGs enriched in this KEGG pathway/the number of DEGS enriched in all KEGG pathway. BTK/BAK represents the number of all background genes enriched in this KEGG pathway/ the number of all background genes enriched in all KEGG pathway.
